# Supplementary material for: Novel Phosphorylated Penta-1,4-dien-3-one Derivatives: Design, Synthesis, and Biological Activity
Source: Molecules. 2019 Mar 7;24(5):925. doi: 10.3390/molecules24050925 (PMC6429309; doi:10.3390/molecules24050925)
Supplement: Supplementary file 1 [file molecules-24-00925-s001.pdf]

Supporting Information

Novel phosphorylated penta-1,4-dien-3-one derivatives: Design, synthesis, and biological activity

Lijuan Chen[†], Tao Guo[†], Rongjiao Xia, Xu Tang, Ying Chen, Cheng Zhang, Wei Xue*

State Key Laboratory Breeding Base of Green Pesticide and Agricultural Bioengineering, Key Laboratory of Green Pesticide and Agriculture Bioengineering, Ministry of Education,
Guizhou University, Huaxi District, Guiyang 550025, China; E-Mails: 13765645934@sina.com (L. C.); guotao9405@163.com (T. G.); xrjiao@163.com (R. X.); tx1173182020@163.com (X. T.); 15286114381@163.com (Y. C.); zhangcheng6954@163.com (C. Z.).

[†] These authors contributed equally to this work.

***** Corresponding author: Wei Xue; e-mail: wxue@gzu.edu.cn (W.X.); Tel/Fax: 0086-851-88292090

Content

1. The physical properties of compounds 3a-3s 2

2. The data of title compounds 3a–3s 2

3. Spectrogram of title compounds 3a-3s 11

1. The physical properties of compounds 3a-3s

Table 1 The physical properties of compounds **3a-3s**

Compd.	R_1	R_2	Appearance	Yield /%	m.p./°C	Values of R_f
**3a**	4-Cl-Ph	CH_3	Yellow solid	48	114-115	0.35
**3b**	4-Cl-Ph	CH_2CH_3	Yellow oil	44	-	0.55
**3c**	4-OCH_3-Ph	CH_3	Yellow solid	52	62-63	0.35
**3d**	4-OCH_3-Ph	CH_2CH_3	Yellow solid	56	72-73	0.55
**3e**	2-Cl-Ph	CH_3	Yellow oil	58	-	0.35
**3f**	2-Cl-Ph	CH_2CH_3	Yellow oil	60	-	0.55
**3g**	3-NO_2-Ph	CH_3	Yellow oil	69	-	0.35
**3h**	3-NO_2-Ph	CH_2CH_3	Yellow solid	65	86-87	0.55
**3i**	4-NO_2-Ph	CH_3	Yellow solid	53	119-120	0.35
**3j**	4-NO_2-Ph	CH_2CH_3	Yellow solid	58	100-101	0.55
**3k**	4-CH_3-Ph	CH_2CH_3	Yellow oil	50	-	0.55
**3l**	Ph	CH_3	Yellow oil	42	-	0.35
**3m**	Ph	CH_2CH_3	Yellow solid	65	70 -71	0.55
**3n**	4-Br-Ph	CH_3	Yellow oil	71	-	0.35
**3o**	4-Br-Ph	CH_2CH_3	Yellow solid	68	79-80	0.55
**3p**	3-CF_3-Ph	CH_3	Yellow solid	63	65-66	0.35
**3o**	3-CF_3-Ph	CH_2CH_3	Yellow oil	72	-	0.55
**3r**	3-CF_3-Ph	CH_3	Yellow oil	69	-	0.35
**3s**	3-CH_3-Ph	CH_2CH_3	Yellow oil	65	-	0.55

2. The data of title compounds 3a–3s

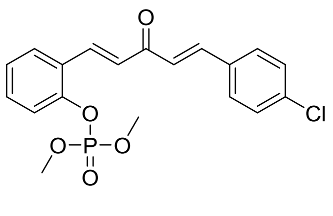

Data for

2-((1E,4E)-5-(4-chlorophenyl)-3-oxopenta-1,4-dien-1-yl)phenyl dimethyl phosphate (**3a**): Yellow solid, m.p. 114-115 ℃, yield 48%. ^{1}H NMR (500 MHz, $CDCl_3$) δ 8.00 (d, *J* = 16.1 Hz, 1H, Ar(2-O)-CH=), 7.67 (s, 1H, Ar-H), 7.64 (d, *J* = 9.8 Hz, 1H, Ar-H), 7.51 (s, 1H, Ar-H),

7.49 (s, 1H, Ar-H), 7.38 – 7.35 (m, 2H, Ar-H, 4-Cl)-CH=), 7.35 (d, *J* = 2.0 Hz, 1H, Ar-H), 7.33 (d, *J* = 1.9 Hz, 1H, Ar-H), 7.22 – 7.17 (m, 1H, Ar-H), 7.08 (d, *J* = 16.0 Hz, 1H, Ar(4-Cl)-C=CH), 7.00 (d, *J* = 16.0 Hz, 1H, Ar(4-Cl)-C=CH), 3.87 (s, 3H, CH_3), 3.85 (s, 3H, CH_3). ^{13}C NMR (126 MHz, $CDCl_3$) δ 188.71, 149.63, 149.58, 142.22, 136.95, 136.53, 133.28, 131.86, 129.65, 129.34, 128.14, 127.07, 126.59, 126.53, 125.93, 125.63, 120.77, 55.32, 55.28. ^{31}P NMR (202 MHz, $CDCl_3$) δ -3.75. HRMS calcd for $C_{19}H_{19}ClO_5P$ $[M+H]^+$ 393.0653, found 393.0645.

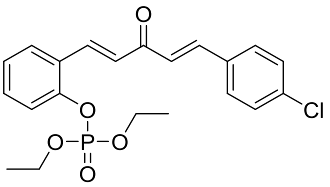

Data for 4-((1E,4E)-5-(4-chlorophenyl)-3-oxopenta-1,4-dien-1-yl)phenyl diethyl phosphate (**3b**): Yellow oil, yield 44%. ^{1}H NMR (400 MHz, $CDCl_3$) δ 8.04 (d, *J* = 16.1 Hz, 1H, Ar(2-O)-CH=), 7.73 – 7.64 (m, 2H, Ar-2H), 7.54 (d, *J* = 8.5 Hz, 2H, Ar-2H), 7.43 (d, *J* = 8.2 Hz, 1H, Ar-H), 7.40 (d, *J* = 9.0 Hz, 2H, Ar(4-Cl)-CH=, Ar-H), 7.36 (s, 1H, Ar-H), 7.22 (t, *J* = 7.4 Hz, 1H, Ar-H), 7.10 (d, *J* = 9.0 Hz, 1H, Ar(2-O)-C=CH), 7.08 – 7.03 (m, 1H, Ar(4-Cl)-C=CH), 4.30 – 4.21 (m, 4H, $2CH_2$), 1.36 (td, *J* = 7.1, 0.8 Hz, 6H, $2CH_3$). ^{13}C NMR (101 MHz, $CDCl_3$) δ 188.69, 149.76, 149.69, 142.00, 137.12, 136.39, 133.27, 131.69, 129.53, 129.23, 127.99, 127.03, 126.54, 126.47, 125.72, 125.36, 120.80, 64.98, 64.92, 16.15, 16.08. ^{31}P NMR (162 MHz, $CDCl_3$) δ -6.43. HRMS calcd for $C_{21}H_{23}ClO_5P$ $[M+H]^+$ 421.0966, found 421.0958.

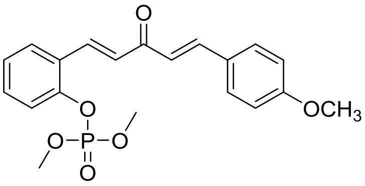

Data for 2-((1E,4E)-5-(4-methoxyphenyl)-3-oxopenta-1,4-dien-1-yl) phenyl dimethyl phosphate (**3c**): Yellow solid, m.p. 62-63 ℃, yield 52%. ^{1}H NMR (500 MHz, $CDCl_3$) δ 8.01 (d, *J* = 16.0 Hz, 1H, Ar(2-O)-CH=), 7.71 (t, *J* = 12.7, 2H, Ar-2H), 7.50 (d, *J* = 8.0 Hz, 2H, Ar-H, Ar(4-OCH_3)-CH=), 7.43 – 7.36 (m, 2H, Ar-2H), 7.22(t, *J* = 8.5 Hz, 3H, Ar-3H), 7.12 (d, *J* = 16.0 Hz, 1H, Ar(2-O)-C=CH), 7.02 (d, *J* = 15.9 Hz, 1H, Ar(4-OCH_3)-C=CH), 3.91 (s, 3H, CH_3), 3.88 (s, 3H, CH_3), 2.38 (s, 3H, CH_3). ^{13}C NMR (126 MHz, $CDCl_3$) δ 189.04, 149.62, 143.86, 141.30, 136.52, 132.04, 131.70, 129.85, 128.65, 128.54, 128.13, 127.79, 127.26,

126.74, 126.69, 125.59, 124.67, 120.75, 55.33, 21.66. ^{31}P NMR (202 MHz, $CDCl_3$) δ -3.78. HRMS calcd for $C_{20}H_{22}O_6P$ $[M+H]^+$ 389.1148, found 389.1124.

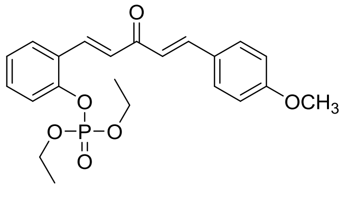

Data for diethyl(2-((1E,4E)-5-(4-methoxyphenyl)-3-oxopenta-1,4-dien-1-yl)phenyl) phosphate (**3d**): Yellow solid, m.p. 72-73 ℃, yield 56%. ^{1}H NMR (400 MHz, $CDCl_3$) δ 8.02 (d, *J* = 16.1 Hz, 1H, Ar(2-O)-CH=), 7.71 (t, *J* = 11.6 Hz, 2H, Ar-2H), 7.57 (d, *J* = 8.7 Hz, 2H, Ar-H, Ar(4-OCH_3)-CH=), 7.47 – 7.35 (m, 2H, Ar-2H), 7.22 (t, *J* = 7.5 Hz, 1H, Ar(2-O)-C=CH), 7.11 (d, *J* = 16.1 Hz, 1H, Ar(4-OCH_3)-C=CH), 6.94 (dd, *J* = 13.0, 10.0, 8.9 Hz, 3H, CH_3), 4.31 – 4.20 (m, 4H, $2CH_2$), 3.85 (s, 3H, CH_3), 1.39 – 1.32 (m, 6H, $2CH_3$). ^{13}C NMR (101 MHz, $CDCl_3$) δ 188.90, 161.72, 149.72, 149.65, 143.42, 136.42, 131.45, 130.17, 127.97, 127.46, 127.35, 126.75, 126.69, 125.30, 123.22, 120.79, 114.47, 64.97, 64.91, 55.43, 16.16, 16.09.^{31}P NMR (162 MHz, $CDCl_3$) δ -6.13. HRMS calcd for $C_{22}H_{26}O_6P$ $[M+H]^+$ 417.1461, found 417.1450.

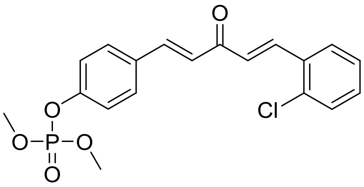

Data for 2-((1E,4E)-5-(2-chlorophenyl)-3-oxopenta-1,4-dien-1-yl)phenyl dimethyl phosphate (**3e**): Yellow oil, yield 58%. ^{1}H NMR (400 MHz, $CDCl_3$) δ 8.14 (d, *J* = 16.1 Hz, 1H, Ar(2-Cl)-CH=), 8.06 (d, *J* = 16.0 Hz, 1H, Ar(2-O)-CH=), 7.72 (d, *J* = 7.2 Hz, 2H, Ar-2H), 7.46 – 7.40 (m, 3H, Ar-3H), 7.37 – 7.31 (m, 2H, Ar-2H), 7.27 – 7.22 (m, 1H, Ar-H), 7.17 (d, *J* = 16.0 Hz, 1H, Ar(2-O)-C=CH), 7.05 (d, *J* = 16.1 Hz, 1H, Ar(2-Cl)-C=CH), 3.92 (s, 3H, CH_3), 3.89 (s, 3H, CH_3). ^{13}C NMR (126 MHz, $CDCl_3$) δ 188.90, 161.81, 149.59, 149.54, 143.63, 136.24, 131.62, 130.30, 128.12, 127.47, 127.37, 126.80, 125.58, 123.45, 120.73, 114.55, 55.52, 55.32, 55.27.^{31}P NMR (162 MHz, $CDCl_3$) δ -3.74. HRMS calcd for $C_{19}H_{19}ClO_5P$ $[M+H]^+$ 393.0653, found 393.0646.

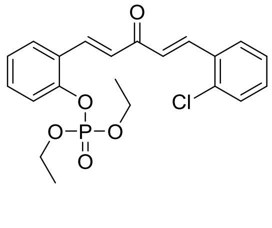

Data for

2-((1E,4E)-5-(2-chlorophenyl)-3-oxopenta-1,4-dien-1-yl)phenyl diethyl phosphate (**3f**): Yellow oil, yield 60%. ^{1}H NMR (500 MHz, $CDCl_3$) δ 8.14 – 7.97 (m, 2H, Ar(2-Cl)-CH=, Ar(2-O)-CH=), 7.67 (dt, *J* = 8.7, 4.4 Hz, 2H, Ar-2H), 7.44 – 7.33 (m, 3H, Ar-3H), 7.28 (ddd, *J* = 6.9, 6.3, 3.2 Hz, 2H, Ar-2H), 7.18 (dd, *J* = 17.3, 9.5 Hz, 1H, Ar-H), 7.13 – 6.97 (m, 2H, Ar(2-O)-C=CH, Ar(2-Cl)-C=CH), 4.39 – 4.05 (m, 4H, $2CH_2$), 1.51 – 1.09 (m, 6H, $2CH_3$). ^{13}C NMR (126 MHz, $CDCl_3$) δ 188.81, 149.82, 149.77, 139.19, 137.39, 135.44, 133.04, 131.84, 131.36, 130.35, 128.02, 127.93, 127.76, 127.24, 126.68, 126.54, 126.49, 125.45, 120.86, 65.10, 16.18. ^{31}P NMR (202 MHz, $CDCl_3$) δ -5.97. HRMS calcd for $C_{21}H_{23}ClO_5P$ $[M+H]^+$ 421.096, found 421.0959.

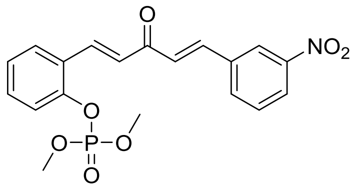

Data for

dimethyl (2-((1E,4E)-5-(2-nitrophenyl)-3-oxopenta-1,4-dien-1-yl)phenyl) phosphate (**3g**): Yellow oil, yield 69%. ^{1}H NMR (400 MHz, $CDCl_3$) δ 8.27 (d, *J* = 8.7 Hz, 2H, Ar-2H), 8.07 (d, *J* = 15.8, 5.9 Hz, 1H, Ar(2-OH)-CH=), 7.79 – 7.70 (m, 4H, Ar-3H, Ar(2-NO_2)-CH=), 7.45 – 7.40 (m, 2H, Ar-2H), 7.25 (ddd, *J* = 9.2, 6.7, 2.6 Hz, 1H, Ar-2H), 7.23 – 7.17 (m, 1H, Ar(2-NO_2)-C=CH), 7.16 – 7.11 (m, 1H, Ar(2-O)-C=CH), 3.93 (s, 3H, CH_3), 3.90 (d, *J* = 3.2 Hz, 3H, CH_3).^{13}C NMR (101 MHz, $CDCl_3$) δ 188.30, 149.66, 149.59, 148.54, 140.97, 140.41, 137.78, 128.95, 128.87, 128.16, 126.85, 126.37, 126.30, 125.66, 124.23, 120.80, 120.78, 55.29, 55.23. ^{31}P NMR (162 MHz, $CDCl_3$) δ -5.01. HRMS calcd for $C_{19}H_{19}NO_7P$ $[M+H]^+$ 404.0893, found 404.0887.

Data for

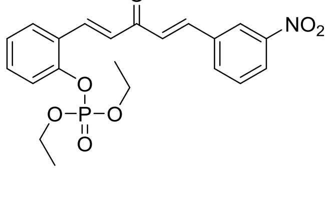

diethyl(2-((1E,4E)-5-(2-nitrophenyl)-3-oxopenta-1,4-dien-1-yl) phenyl) phosphate (**3h**): Yellow solid, m.p. 86-87 ℃, yield 65%. ^{1}H NMR (400 MHz, $CDCl_3$) δ 8.26 (d, *J* = 8.8 Hz, 2H, Ar-2H), 8.07 (d, *J* = 16.2 Hz, 1H, Ar(2-OH)-CH=), 7.80 – 7.72 (m, 3H, Ar-2H, Ar(2-NO_2)-CH=), 7.70 (d, *J* = 7.8 Hz, 1H, Ar-H), 7.46 – 7.37 (m, 2H, Ar-2H), 7.26 – 7.17 (m, 2H, Ar-2H, Ar(2-NO_2)-C=CH), 7.12 (d, *J* = 16.1 Hz, 1H, Ar(2-O)-C=CH), 4.32 –

4.19 (m, 4H, 2CH_2), 1.36 (td, J = 7.1, 1.0 Hz, 6H, 2CH_3).^{13}C NMR (101 MHz, $CDCl_3$) δ 188.33, 149.89, 149.81, 148.55, 141.04, 140.26, 138.05, 131.96, 128.90, 128.76, 128.09, 126.87, 126.39, 126.33, 125.42, 124.20, 120.94, 65.01, 64.94, 16.16, 16.10. ^{31}P NMR (162 MHz, $CDCl_3$) δ -6.40. HRMS calcd for $C_{21}H_{23}NO_7P$ $[M+H]^+$ 432.1206, found 432.1199.

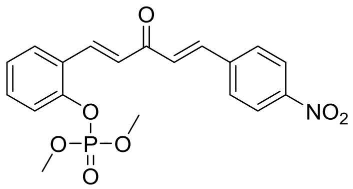

Data for

dimethyl(2-((1E,4E)-5-(4-nitrophenyl)-3-oxopenta-1,4-dien-1-yl)phenyl) phosphate (**3i**): Yellow solid, m.p. 119-120 ℃, yield 53%. ^{1}H NMR (500 MHz, $CDCl_3$) δ 8.48 – 8.40 (m, 1H, Ar-H), 8.23 – 8.18 (m, 1H, Ar-H), 8.04 (d, J = 16.1 Hz, 1H, Ar-H), 7.87 (d, J = 7.0 Hz, 1H, Ar-H), 7.71 (dd, J = 24.1, 12.2 Hz, 2H, Ar-2H), 7.58 (t, J = 8.0 Hz, 1H, Ar(2-OH)-CH=), 7.38 (d, J = 3.6 Hz, 2H, Ar-H, Ar(4-NO2)-CH=), 7.18 (d, J = 15.9 Hz, 2H, Ar(4-NO2)-C=CH), 7.09 (d, J = 16.1 Hz, 1H, Ar(2-O)-C=CH), 3.90 (s, 3H, CH_3), 3.87 (s, 3H, CH_3).^{13}C NMR (126 MHz, $CDCl_3$) δ 188.35, 149.64, 148.74, 140.55, 137.62, 136.61, 134.33, 132.11, 130.18, 128.11, 127.69, 127.02, 126.41, 125.69, 124.75, 122.44, 120.85, 55.36, 55.31. ^{31}P NMR (202 MHz, $CDCl_3$) δ -6.63. HRMS calcd for $C_{19}H_{19}NO_7P$ $[M+H]^+$ 404.0893, found 404.0887.

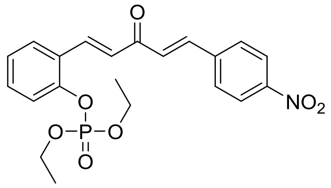

Data for

diethyl(2-((1E,4E)-5-(4-nitrophenyl)-3-oxopenta-1,4-dien-1-yl)phenyl) phosphate (**3j**): Yellow solid, m.p. 100-101 ℃, yield 58%. ^{1}H NMR (400 MHz, $CDCl_3$) δ 8.48 (s, 1H, Ar-H), 8.25 (d, J = 8.2 Hz, 1H, Ar-H), 8.08 (d, J = 16.2 Hz, 1H, Ar(2-OH)-CH=), 7.91 (d, J = 7.7 Hz, 1H, Ar-H), 7.77 (d, J = 16.0 Hz, 1H, Ar(4-NO2)-CH=), 7.71 (d, J = 7.8 Hz, 1H, Ar-H), 7.62 (t, J = 8.0 Hz, 1H, Ar-H), 7.48 – 7.38 (m, 2H, Ar-2H), 7.27 – 7.19 (m, 2H, Ar-H, Ar(2-O)-C=CH), 7.12 (d, J = 16.1 Hz, 1H, Ar(4-NO2)-C=CH), 4.34 – 4.20 (m, 4H, 2CH_2), 1.40 – 1.33 (m, 6H, 2CH_3). ^{13}C NMR (101 MHz, $CDCl_3$) δ 188.33, 149.85, 148.74, 140.38, 137.85, 136.62, 134.13, 131.91, 130.06, 128.03, 127.62, 126.96, 126.40, 125.41, 124.61, 122.39, 120.93, 65.02, 64.96, 16.15, 16.09. ^{31}P NMR (162 MHz, $CDCl_3$) δ -7.04. HRMS calcd for $C_{21}H_{23}NO_7P$ $[M+H]^+$ 432.1206, found 432.1198.

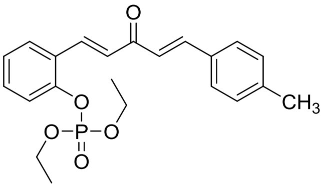

Data for

diethyl (2-((1E,4E)-3-oxo-5-(p-tolyl)penta-1,4-dien-1-yl)phenyl) phosphate (**3k**): Yellow oil, yield 50%. ^{1}H NMR (400 MHz, $CDCl_3$) δ 8.10 – 8.00 (m, 2H, Ar(2-OH)-CH=, Ar-H), 7.71 (d, *J* = 7.8 Hz, 1H, Ar-H), 7.60 (dd, *J* = 7.7, 1.5 Hz, 1H, Ar-H), 7.45 (d, *J* = 8.3 Hz, 1H, Ar(4-CH_3)-CH=), 7.40 – 7.34 (m, 2H, Ar-2H), 7.18 (td, *J* = 16.2, 10.6 Hz, 3H, Ar-3H), 7.01 – 6.91 (m, 2H, Ar(4-CH_3)-C=CH, Ar(2-O)-C=CH), 4.30 – 4.21 (m, 4H, 2CH_2), 3.92 (s, 3H, CH_3), 1.38 – 1.31 (m, 6H, 2CH_3). ^{13}C NMR (101 MHz, $CDCl_3$) δ 189.43, 158.69, 149.71, 149.64, 138.96, 136.43, 131.83, 131.43, 128.97, 127.88, 127.20, 126.79, 126.11, 125.27, 123.71, 120.80, 111.25, 64.97, 64.91, 55.52, 16.13, 16.06. ^{31}P NMR (162 MHz, $CDCl_3$) δ -6.41. HRMS calcd for $C_{22}H_{26}O_5P$ $[M+H]^+$401.1512, found 401.1497.

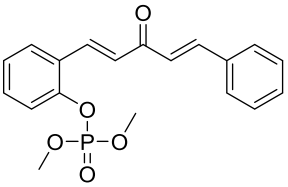

Data for

dimethyl (2-((1E,4E)-3-oxo-5-phenylpenta-1,4-dien-1-yl)phenyl) phosphate (**3l**): Yellow oil, yield 42%. ^{1}H NMR (400 MHz, $CDCl_3$) δ 8.03 (d, *J* = 16.1 Hz, 1H, Ar(2-OH)-CH=), 7.68 (t, *J* = 10.9 Hz, 2H, Ar-2H), 7.53 (d, *J* = 6.9 Hz, 2H, Ar-1H, Ar-CH=), 7.46 (d, *J* = 7.2 Hz, 2H, Ar-2H), 7.40 (d, *J* = 6.2 Hz, 2H, Ar-2H), 7.22 (m, 4.2 Hz, 1H, Ar-H), 7.11 (d, *J* = 16.1 Hz, 1H, Ar-C=CH), 7.05 (d, *J* = 16.0 Hz, 1H, Ar(2-OH)-C=CH), 3.91 (s, 3H, CH_3), 3.88 (s, 3H, CH_3).^{13}C NMR (101 MHz, $CDCl_3$) δ 188.88, 149.59, 149.52, 143.63, 136.65, 134.74, 131.65, 130.60, 129.00, 128.40, 128.09, 127.14, 126.65, 126.58, 125.54, 120.70, 120.68, 55.20, 55.14. ^{31}P NMR (162 MHz, $CDCl_3$) δ -5.03. HRMS calcd for $C_{19}H_{20}O_5P$ $[M+H]^+$ 359.1042, found 359.1030.

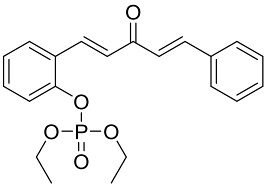

Data for

diethyl (2-((1E,4E)-3-oxo-5-phenylpenta-1,4-dien-1-yl)phenyl) phosphate (**3m**): Yellow solid, m.p. 70-71 ℃, yield 65%. ^{1}H NMR (400 MHz, $CDCl_3$) δ 8.04 (d, *J* = 16.1 Hz, 1H, Ar(2-OH)-CH=), 7.75 (d, *J* = 16.0 Hz, 1H, Ar-H), 7.70 (d, *J* = 7.8 Hz, 1H, Ar-H), 7.61 (dd, *J* = 9.5, 3.7 Hz, 2H, Ar-H,

Ar-CH=), 7.47 – 7.36 (m, 5H, Ar-5H), 7.25 – 7.20 (m, 1H, Ar-H), 7.13 (d, *J* = 10.8 Hz, 1H, Ar-C=CH), 7.09 (d, *J* = 10.7 Hz, 1H, Ar(2-O)-C=CH), 4.31 – 4.19 (m, 4H, $2CH_2$), 1.39 – 1.31 (m, 6H, $2CH_3$).^{13}C NMR (101 MHz, $CDCl_3$) δ 188.99, 149.78, 149.71, 143.54, 136.95, 134.79, 131.59, 130.56, 128.99, 128.39, 128.00, 127.18, 126.65, 126.59, 125.33, 120.84, 120.82, 64.97, 64.91, 16.15, 16.09. ^{31}P NMR (162 MHz, $CDCl_3$) δ -6.18. HRMS calcd for $C_{21}H_{24}O_5P$ $[M+H]^+$ 387.1355, found 387.1346.

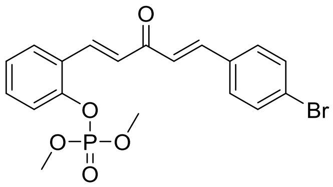

Data for 2-((1E,4E)-5-(4-bromophenyl)-3-oxopenta-1,4-dien-1-yl)phenyl dimethyl phosphate (**3n**): Yellow oil, yield 71%. ^{1}H NMR (400 MHz, $CDCl_3$) δ 8.03 (d, *J* = 16.1 Hz, 1H, Ar(2-OH)-CH=), 7.68 (t, *J* = 10.9 Hz, 2H, Ar-2H), 7.53 (d, *J* = 6.9 Hz, 2H, Ar-2H), 7.46 (d, *J* = 7.2 Hz, 2H, Ar-H, Ar-(4-Br)-CH=), 7.42 – 7.36 (m, 2H, Ar-2H), 7.22 (dd, *J* = 9.7, 4.2 Hz, 1H, Ar-H), 7.11 (d, *J* = 16.1 Hz, 1H, Ar-(4-Br)-C=CH), 7.05 (d, *J* = 16.0 Hz, 1H, Ar(2-O)-C=CH), 3.91 (s, 3H, CH_3), 3.88 (s, 3H, CH_3).^{13}C NMR (101 MHz, $CDCl_3$) δ 188.61, 149.60, 149.53, 142.15, 136.92, 133.69, 132.23, 131.74, 129.74, 128.09, 127.06, 126.56, 126.50, 125.98, 125.53, 124.83, 120.70, 55.21, 55.15. ^{31}P NMR (162 MHz, $CDCl_3$) δ -4.50. HRMS calcd for $C_{19}H_{19}BrO_5P$ $[M+H]^+$ 437.0148, found 437.0134.

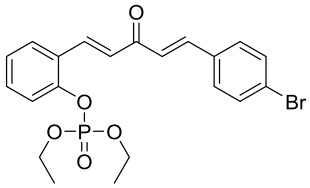

Data for 2-((1E,4E)-5-(4-bromophenyl)-3-oxopenta-1,4-dien-1-yl)phenyl diethyl phosphate (**3o**): Yellow solid, m.p.79-80 ℃, yield 68%. ^{1}H NMR (500 MHz, $CDCl_3$) δ 8.05 (d, *J* = 16.1 Hz, 1H, Ar(2-OH)-CH=), 7.74 – 7.66 (m, 2H, Ar-2H), 7.56 (d, *J* = 8.5 Hz, 2H, Ar-H, Ar-(4-Br)-CH=), 7.49 (d, *J* = 8.4 Hz, 2H, Ar-2H), 7.45 (d, *J* = 9.4 Hz, 1H, Ar-H), 7.43 – 7.39 (m, 1H, Ar-H), 7.24 (m, 1H, Ar-H), 7.12 (d, *J* = 12.1 Hz, 1H, Ar-(4-Br)-C=CH), 7.09 (d, *J* = 12.0 Hz, 1H, Ar(2-O)-C=CH), 4.31 – 4.23 (m, 4H, $2CH_2$), 1.40 – 1.34 (m, 6H, $2CH_3$). ^{13}C NMR (126 MHz, $CDCl_3$) δ 188.80, 149.87, 149.82, 142.15, 137.32, 133.81, 132.31, 131.76, 131.75, 128.10, 127.18, 126.64, 126.59, 125.86, 125.42, 124.88, 120.94, 65.04, 64.99, 16.22, 16.17. ^{31}P NMR

(202 MHz, $CDCl_3$) δ -3.42. HRMS calcd for $C_{21}H_{23}BrO_5P$ $[M+H]^+$ 465.0461, found 465.0451.

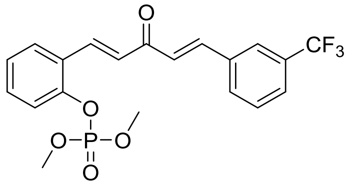

Data for dimethyl(2-((1E,4E)-3-oxo-5-(3-(trifluoromethyl)phenyl)penta-1,4-dien-1-yl)phenyl) phosphate (**3p**): Yellow solid, m.p. 65-66 ℃, yield 63%.^{1}H NMR (400 MHz, $CDCl_3$) δ 8.07 (d, *J* = 16.1 Hz, 1H, Ar(2-OH)-CH=), 7.87 (s, 1H, Ar-H), 7.77 (s, 1H, Ar-(3-CF_3)-CH=), 7.73 (d, *J* = 5.3 Hz, 1H, Ar-H), 7.66 (d, *J* = 7.6 Hz, 1H, Ar-H), 7.58 – 7.52 (m, 2H, Ar-2H), 7.42 (s, 2H, Ar-2H), 7.14 (dd, *J* = 16.0, 1.5 Hz, 3H, Ar-H, Ar(2-O)-C=CH, Ar-(3-CF_3)-C=CH), 3.92 (s, 3H, CH_3), 3.90 (s, 3H, CH_3).^{13}C NMR (101 MHz, $CDCl_3$) δ 188.54, 149.62, 149.56, 141.61, 137.28, 135.58, 131.90, 131.58, 129.59, 128.03, 127.02, 126.80, 125.58, 125.17, 124.66, 124.63, 120.78, 120.76, 55.23, 55.17. ^{31}P NMR (162 MHz, $CDCl_3$) δ -4.78. HRMS calcd for $C_{20}H_{19}F_3O_5P$ $[M+H]^+$ 427.0916, found 427.0914.

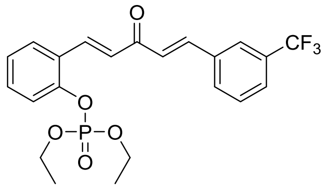

Data for diethyl(2-((1E,4E)-3-oxo-5-(3-(trifluoromethyl)phenyl)penta-1,4-dien-1-yl)phenyl) phosphate (**3q**): Yellow oil, yield 72%. ^{1}H NMR (400 MHz, $CDCl_3$) δ 8.07 (d, *J* = 16.2 Hz, 1H, Ar(2-OH)-CH=), 7.87 (s, 1H, Ar-H), 7.77 (dd, *J* = 11.8, 8.7 Hz, 2H, Ar-(3-CF_3)-CH=, Ar-H), 7.71 (d, *J* = 7.8 Hz, 1H, Ar-H), 7.66 (d, *J* = 7.8 Hz, 1H, Ar-2=H), 7.56 (d, *J* = 8.0 Hz, 1H, Ar-H), 7.47 – 7.40 (m, 2H, Ar-2H), 7.24 (t, *J* = 7.2 Hz, 1H, Ar-H), 7.17 (d, *J* = 16.0 Hz, 1H, Ar(2-O)-C=CH), 7.11 (d, *J* = 16.1 Hz, 1H, Ar-(3-CF_3)-C=CH), 4.30 – 4.22 (m, 4H, $2CH_2$), 1.39 – 1.34 (m, 6H, $2CH_3$).^{13}C NMR (101 MHz, $CDCl_3$) δ 188.63, 149.82, 149.75, 141.55, 137.57, 135.62, 131.84, 131.57, 129.57, 128.00, 127.03, 126.67, 126.40, 125.39, 124.66, 124.63, 120.92, 120.90, 65.01, 64.95, 16.15, 16.09. ^{31}P NMR (162 MHz, $CDCl_3$) δ -7.27. HRMS calcd for $C_{22}H_{23}F_3O_5P$ $[M+H]^+$ 455.1229, found 455.1223.

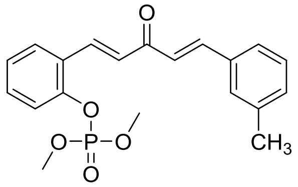

Data for

dimethyl (2-((1E,4E)-3-oxo-5-(p-tolyl)penta-1,4-dien-1-yl)phenyl) phosphate (**3r**): Yellow oil, yield 69%. ^{1}H NMR (400 MHz, $CDCl_3$) δ 8.03 (d, *J* = 16.1 Hz, 1H, Ar(2-OH)-CH=), 7.71 (dd, *J* = 11.9, 7.1 Hz, 2H, Ar-2H), 7.44 – 7.36 (m, 4H, Ar-(3-CH_3)-CH=, Ar-3H), 7.30 (dd, *J* = 12.4, 4.3 Hz, 1H, Ar-H), 7.22 (d, *J* = 7.6 Hz, 2H, Ar-2H), 7.14 (d, *J* = 16.0 Hz, 1H, Ar(2-O)-C=CH), 7.06 (d, *J* = 16.0 Hz, 1H, Ar-(3-CH_3)-C=CH), 3.92 (s, 3H, CH_3), 3.89 (s, 3H, CH_3), 2.39 (s, 3H, CH_3). ^{13}C NMR (101 MHz, $CDCl_3$) δ 188.91, 149.49, 143.87, 138.66, 136.54, 134.66, 131.65, 131.50, 129.06, 128.90, 128.05, 127.12, 126.65, 126.58, 125.65, 125.52, 125.37, 120.69, 55.23, 21.36. ^{31}P NMR (162 MHz, $CDCl_3$) δ -4.15. HRMS calcd for $C_{20}H_{22}O_5P$ $[M+H]^+$ 373.1199, found 373.1195.

Data for

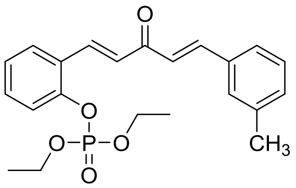

diethyl(2-((1E,4E)-3-oxo-5-(m-tolyl)penta-1,4-dien-1-yl)phenyl) phosphate (**3s**): Yellow oil, yield 65%. ^{1}H NMR (400 MHz, $CDCl_3$) δ 8.04 (d, *J* = 16.1 Hz, 1H, Ar(2-OH)-CH=), 7.71 (dd, *J* = 11.8, 9.8 Hz, 2H, Ar-H, Ar-(3-CH_3)-CH=), 7.46 – 7.36 (m, 4H, Ar-4H), 7.32 – 7.27 (m, 1H, Ar-H), 7.22 (ddd, *J* = 7.1, 5.4, 1.4 Hz, 2H, Ar-2H), 7.10 (m, 12.4 Hz, 2H, Ar(2-O)-C=CH, Ar-(3-CH_3)-C=CH), 4.30 – 4.22 (m, 4H, $2CH_2$), 2.39 (s, 3H, CH_3), 1.38 – 1.32 (m, 6H, $2CH_3$).^{13}C NMR (101 MHz, $CDCl_3$) δ 189.00, 149.74, 143.77, 138.64, 136.81, 134.70, 131.59, 131.45, 129.04, 128.88, 127.97, 127.17, 126.64, 125.64, 125.33, 125.16, 120.79, 64.99, 64.93, 21.36, 16.17, 16.10. ^{31}P NMR (162 MHz, $CDCl_3$) δ -7.30. HRMS calcd for $C_{22}H_{26}O_5P$ $[M+H]^+$ 401.1512, found 401.1510.

3. Spectrogram of title compounds 3a-3s

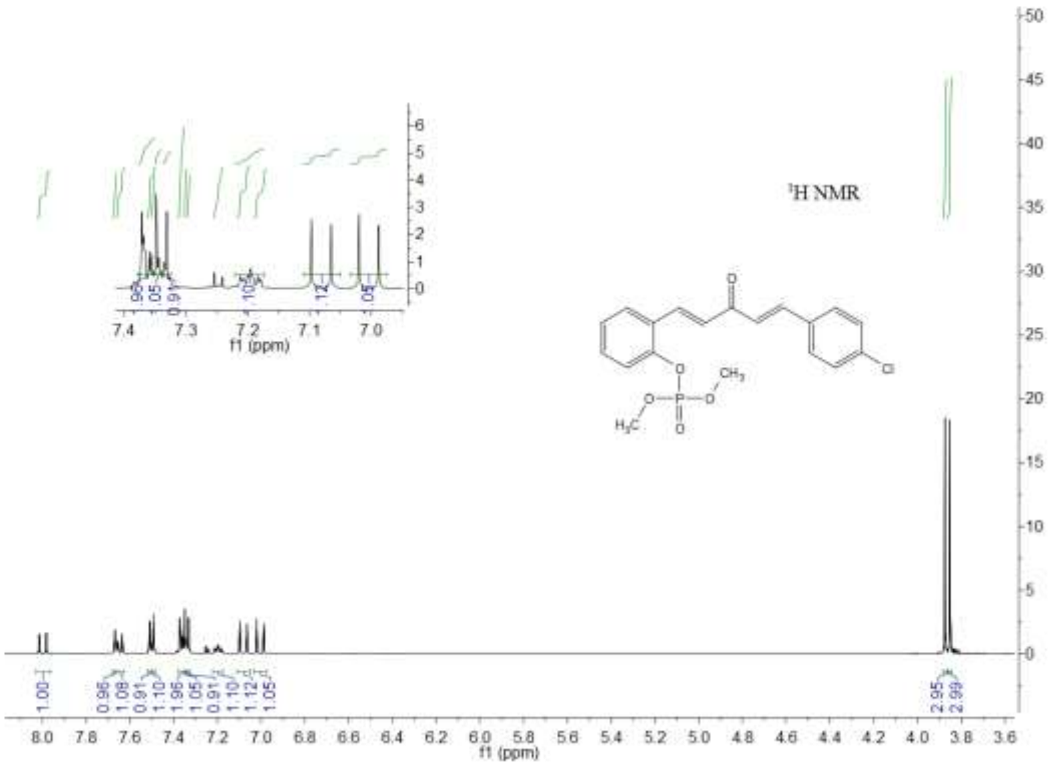

^{1}H NMR of compound **3a**

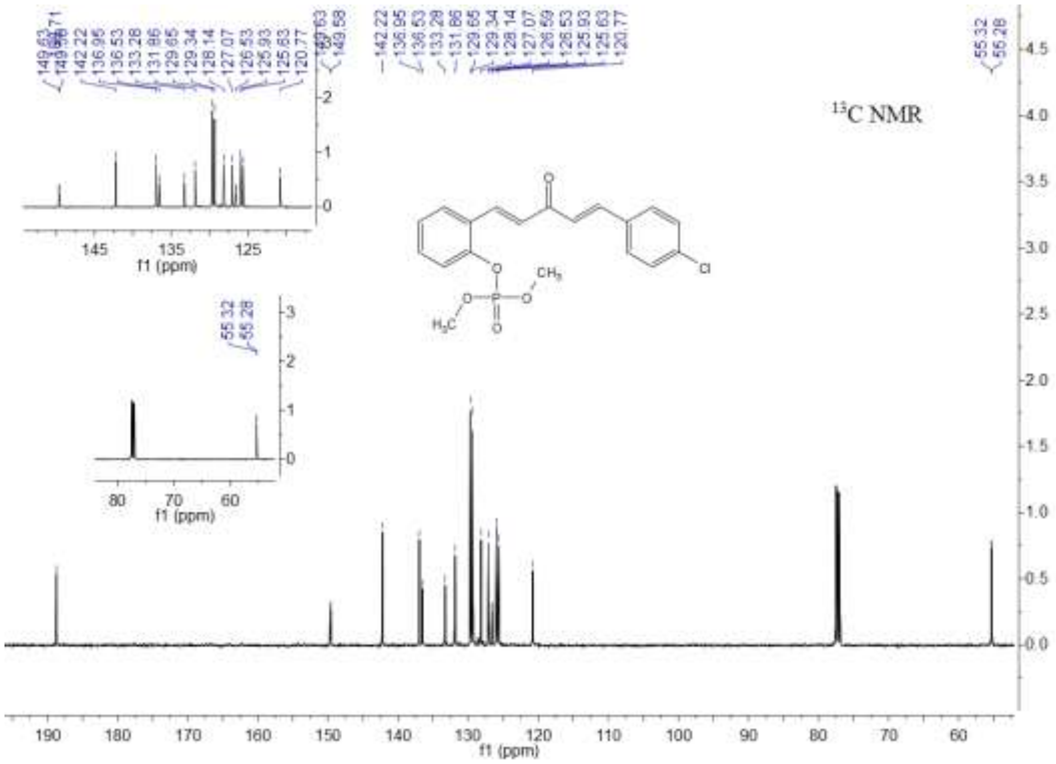

^{13}C NMR of compound **3a**

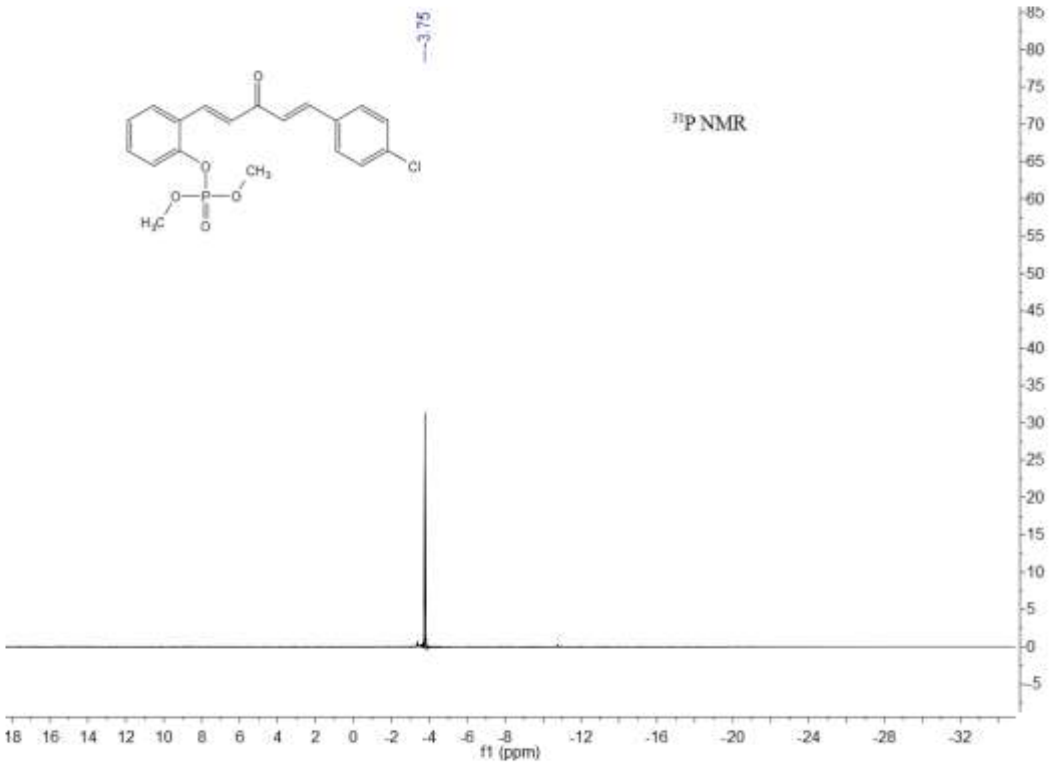

^{31}P NMR of compound **3a**

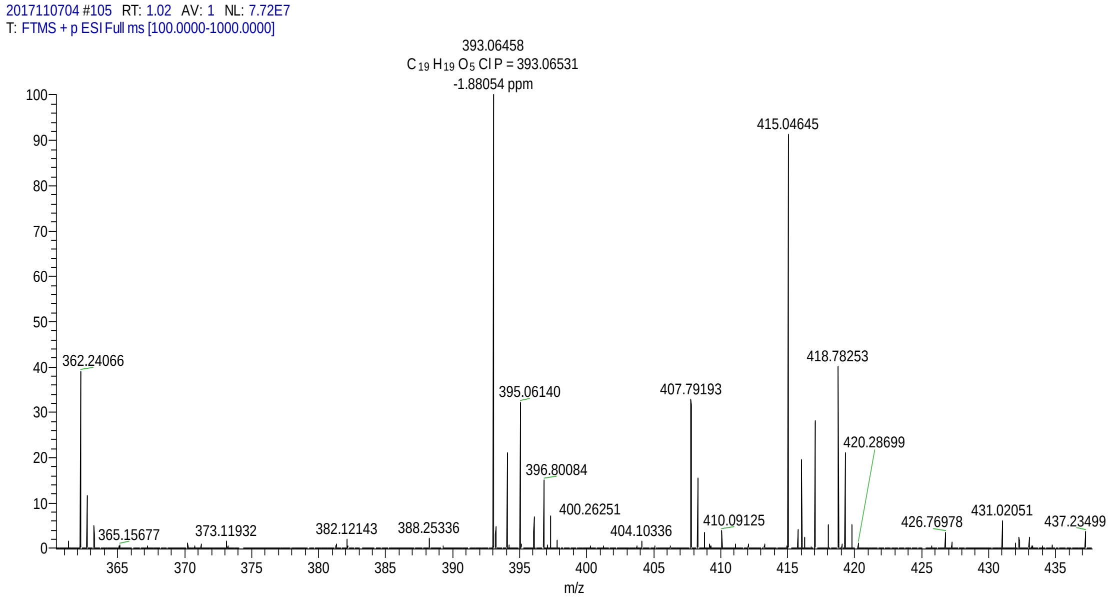

HRMS of compound **3a**

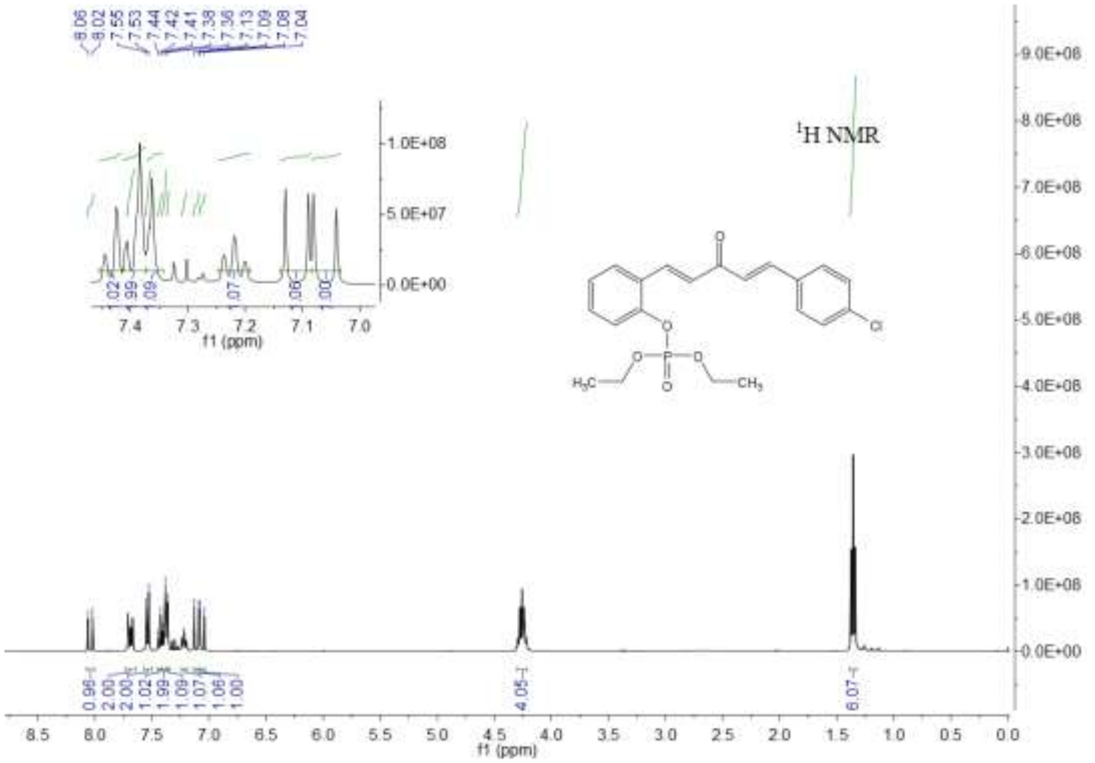

^{1}H NMR of compound **3b**

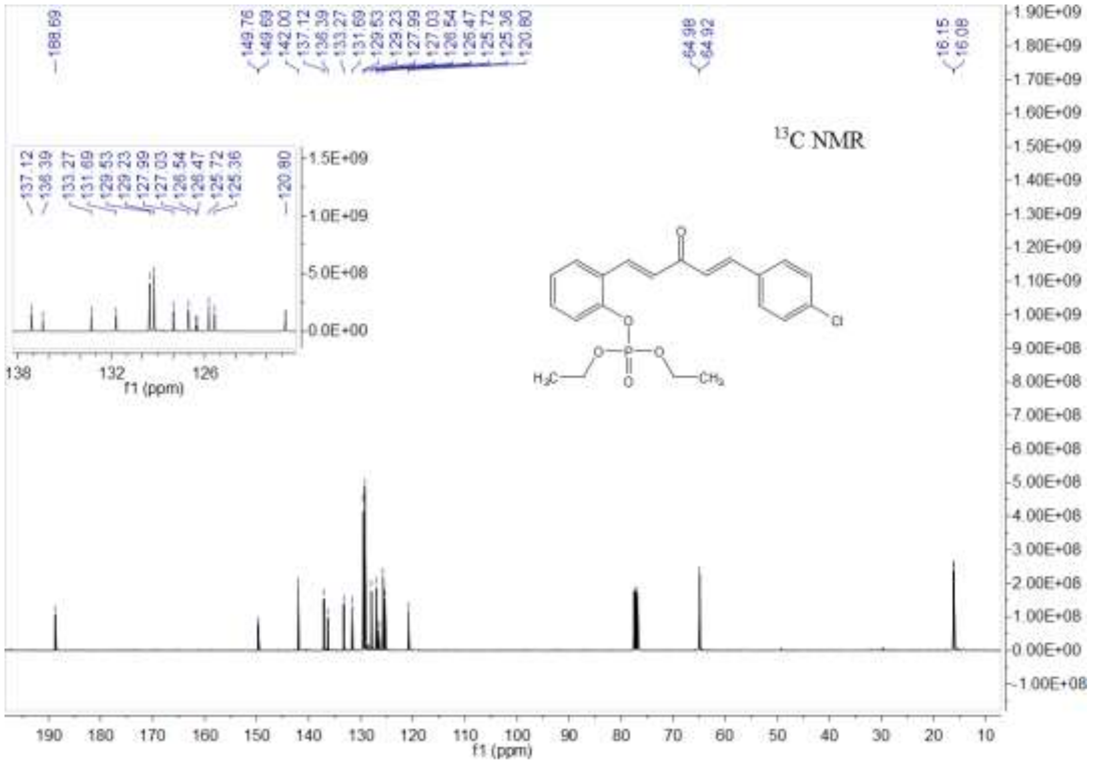

^{13}C NMR of compound **3b**

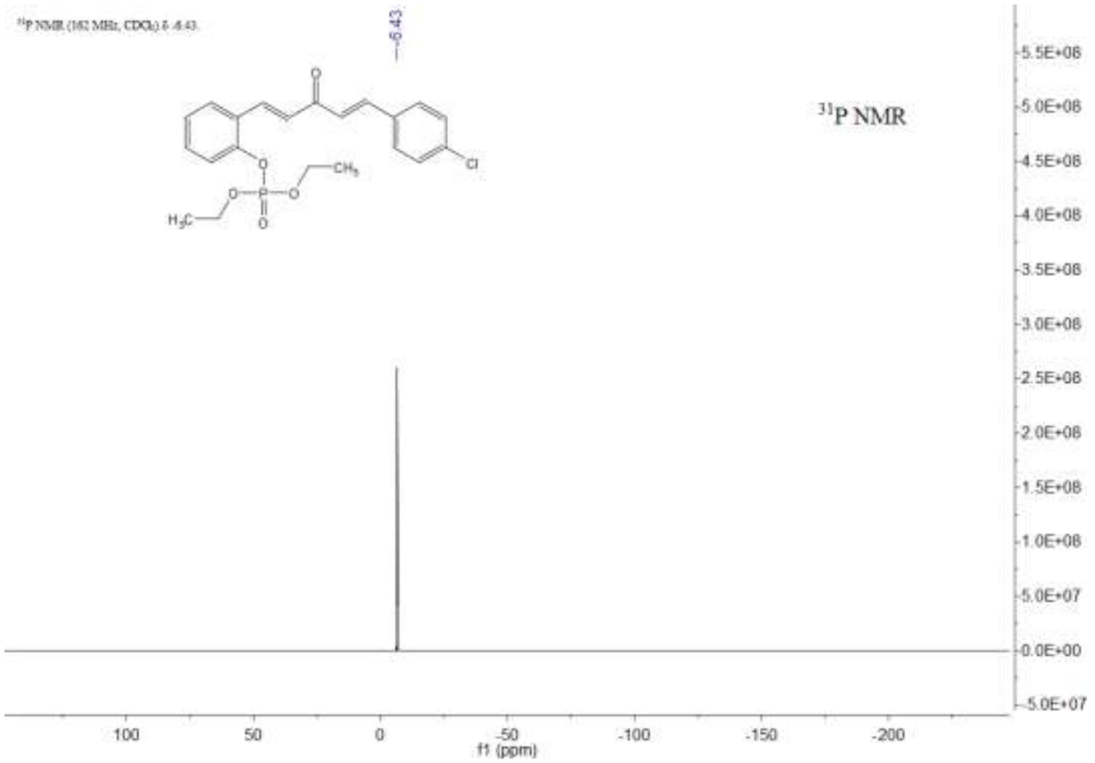

^{31}P NMR of compound **3b**

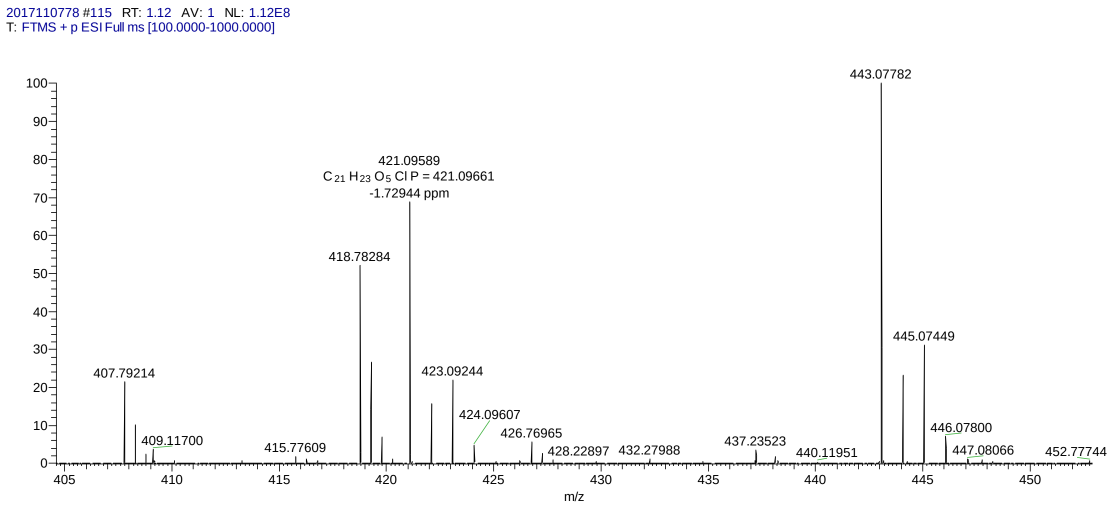

HRMS of compound **3b**

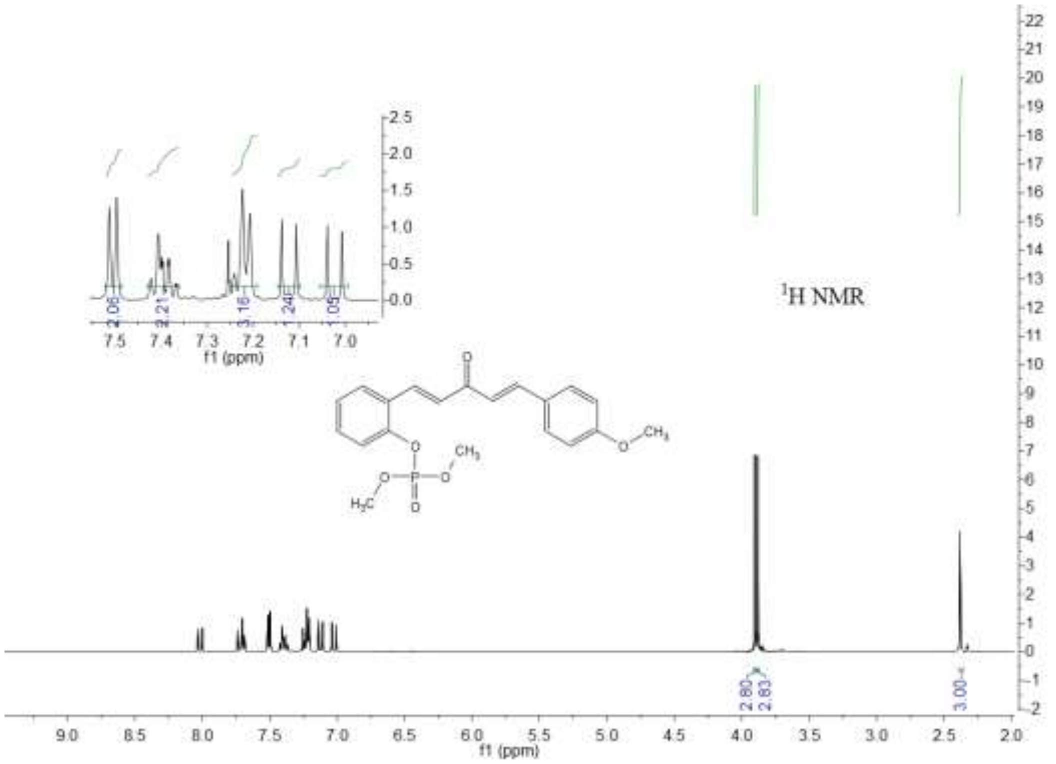

^{1}H NMR of compound **3c**

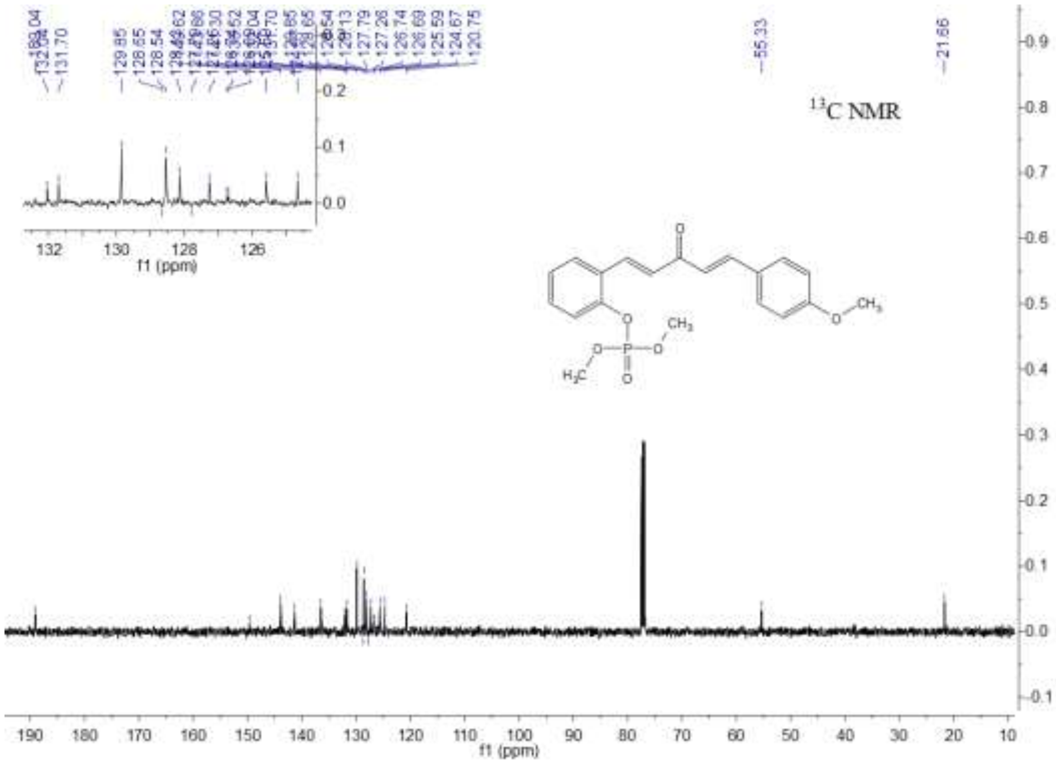

^{13}C NMR of compound **3c**

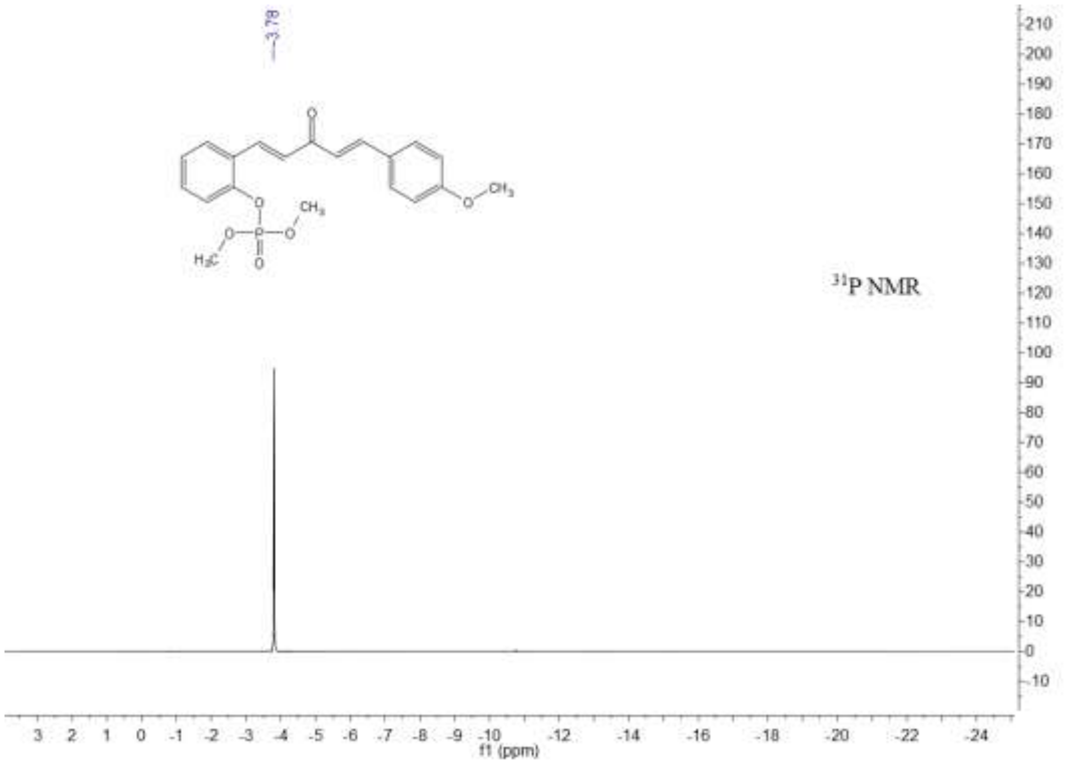

^{31}P NMR of compound **3c**

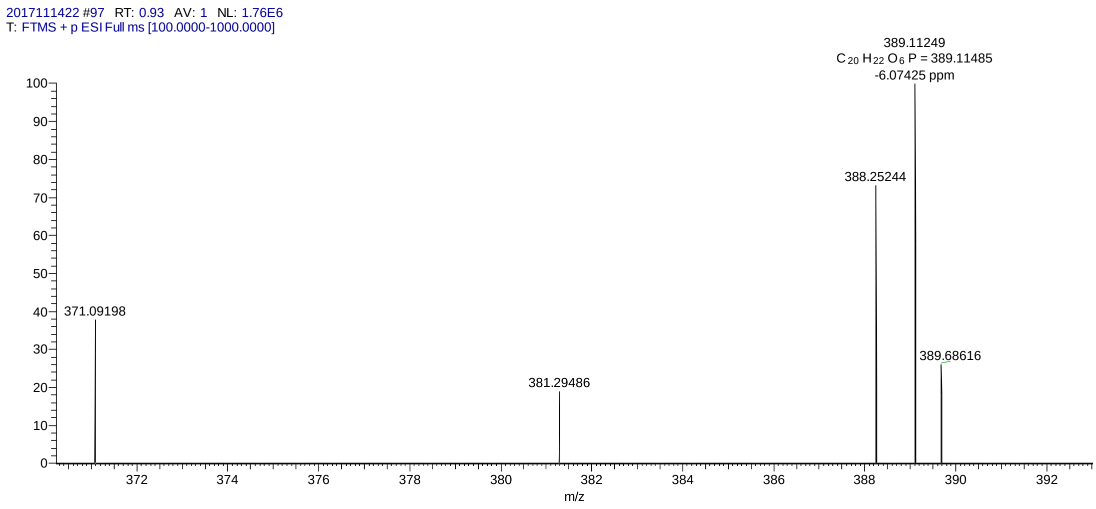

HRMS of compound **3c**

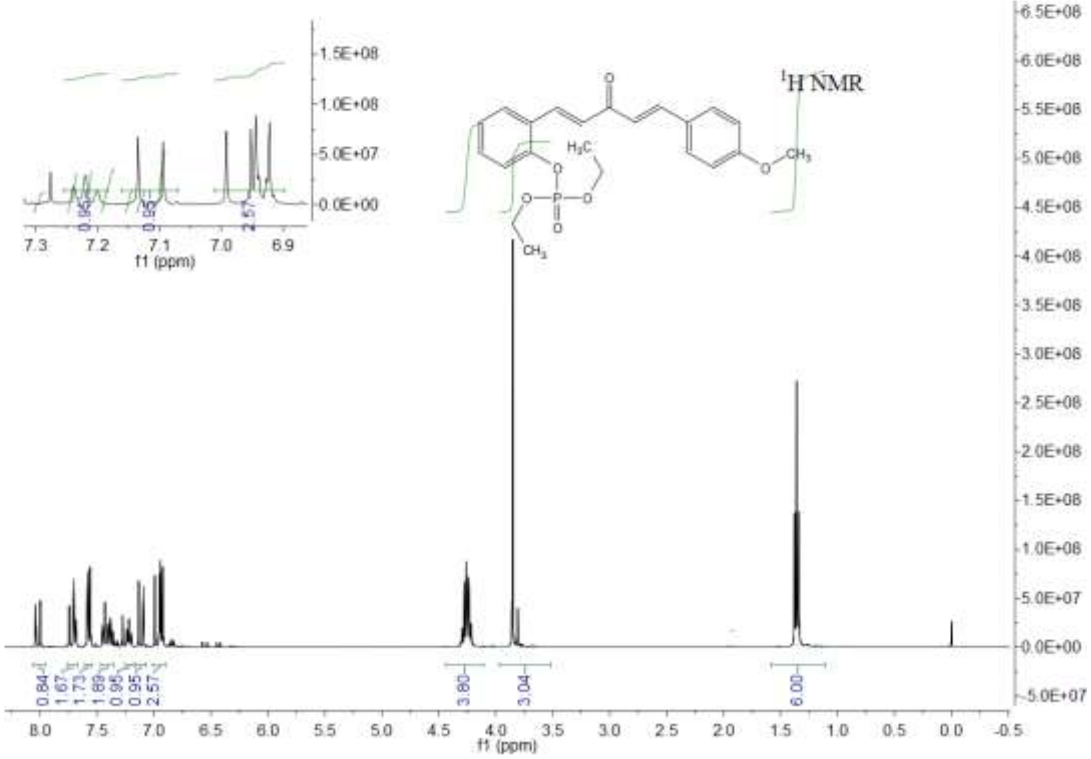

^{1}H NMR of compound **3d**

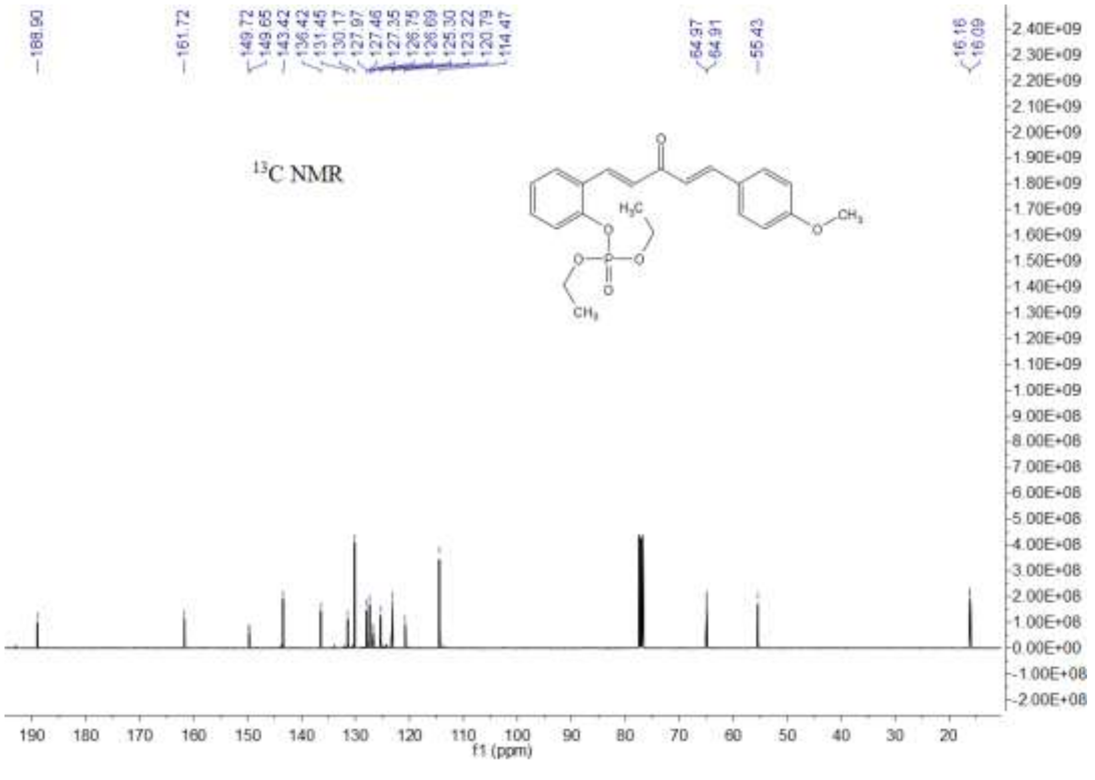

^{13}C NMR of compound **3d**

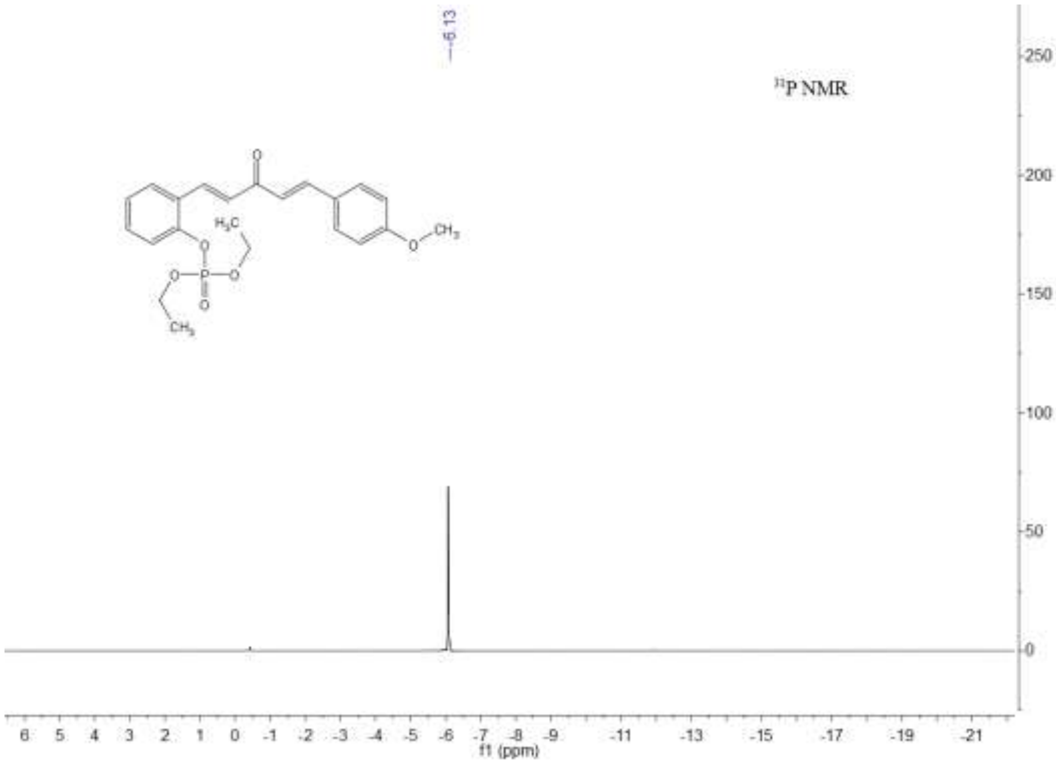

^{31}P NMR of compound **3d**

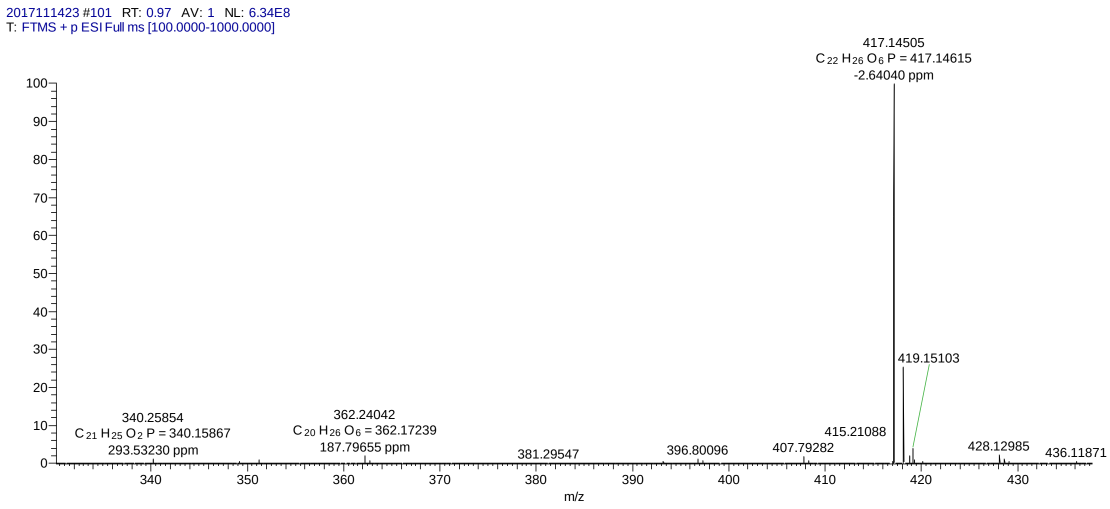

HRMS of compound **3d**

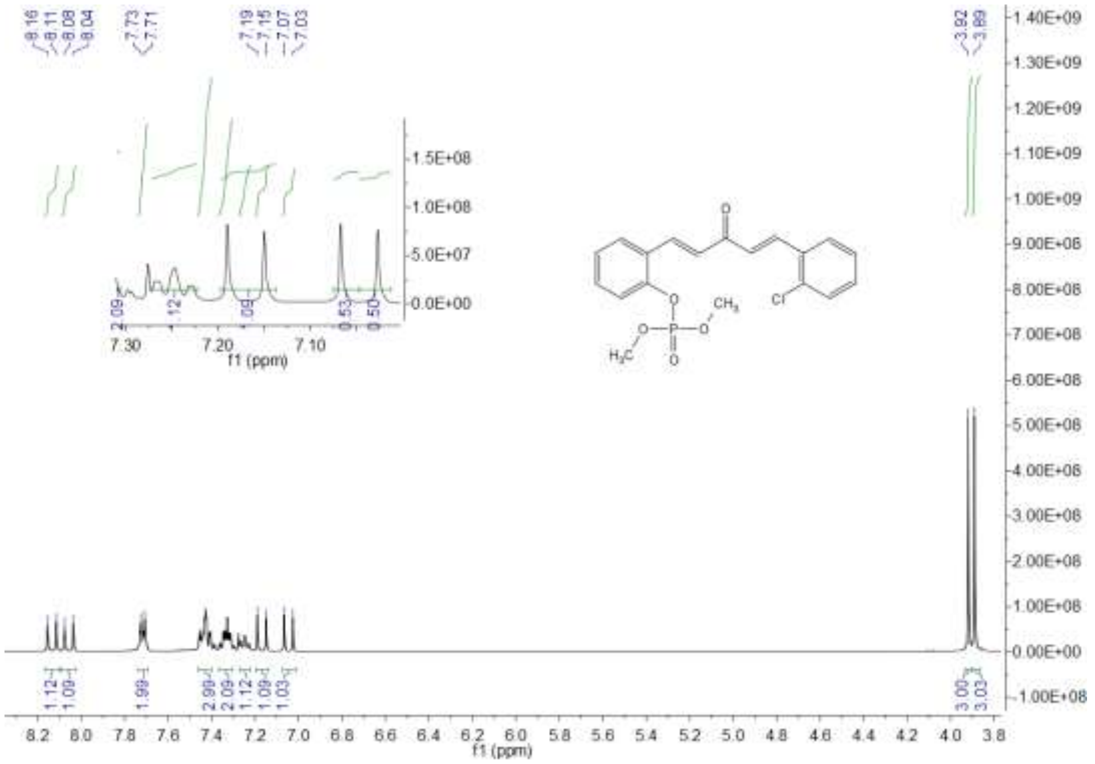

^{1}H NMR of compound **3e**

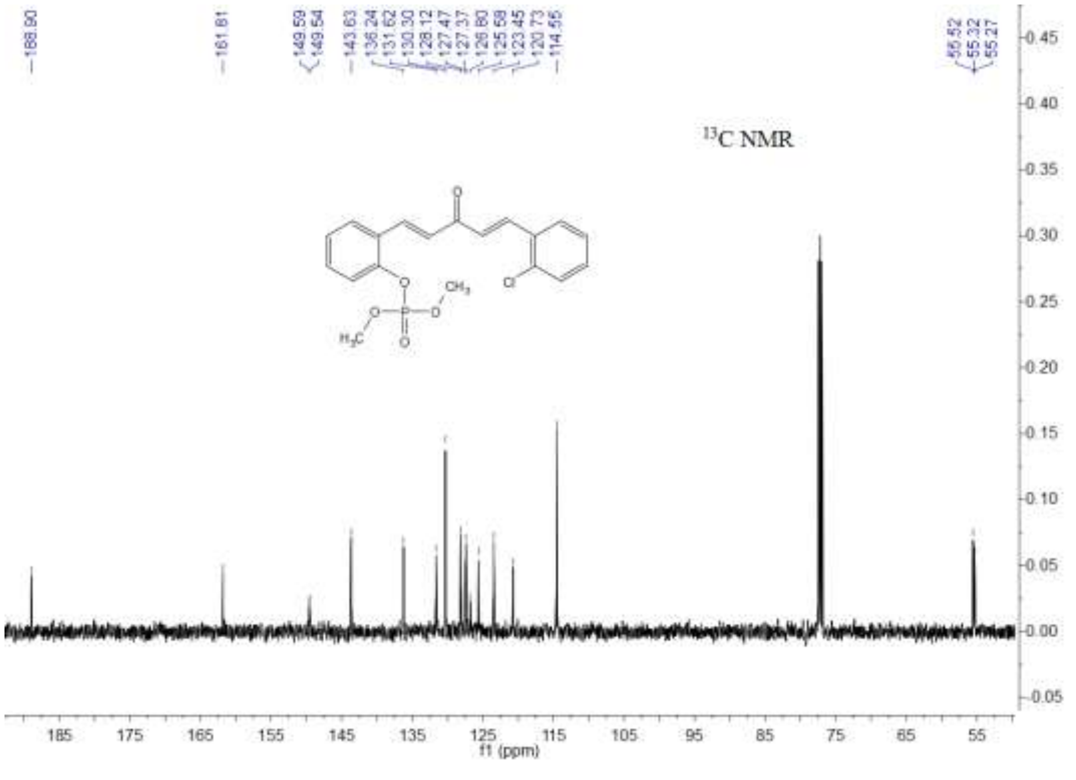

^{13}C NMR of compound **3e**

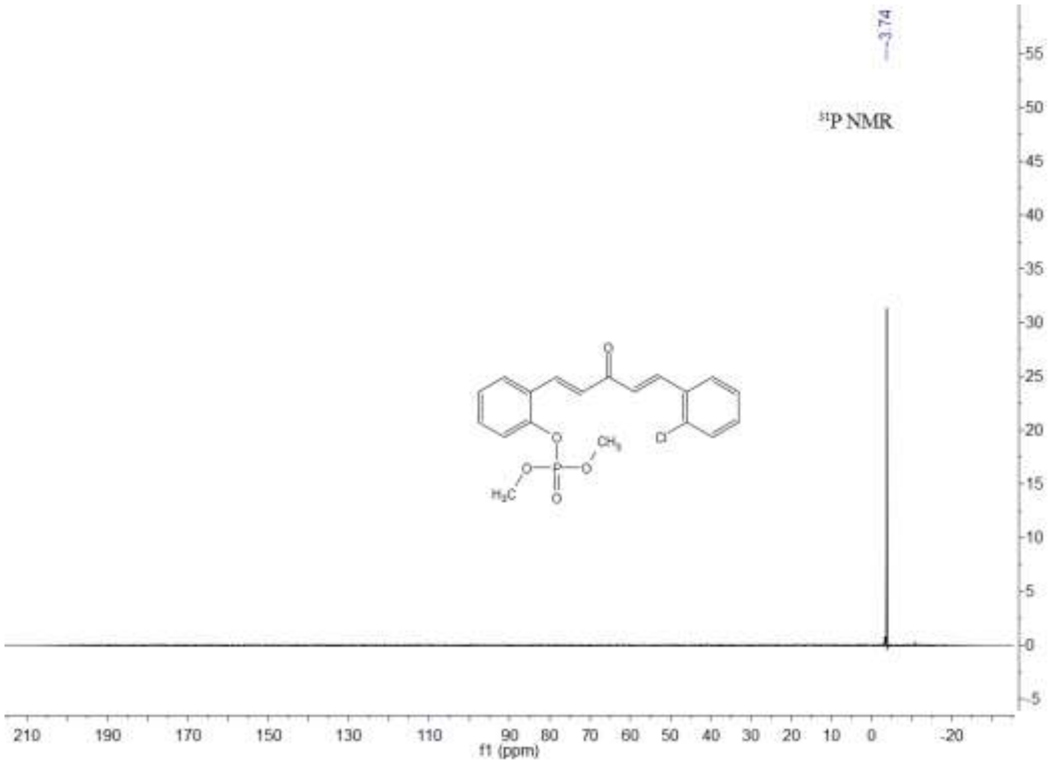

^{31}P NMR of compound **3e**

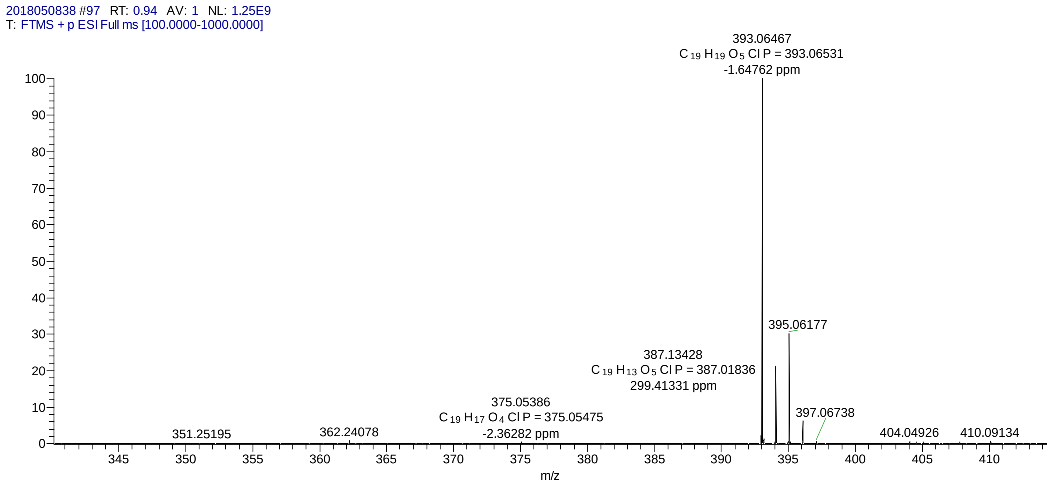

HRMS of compound **3e**

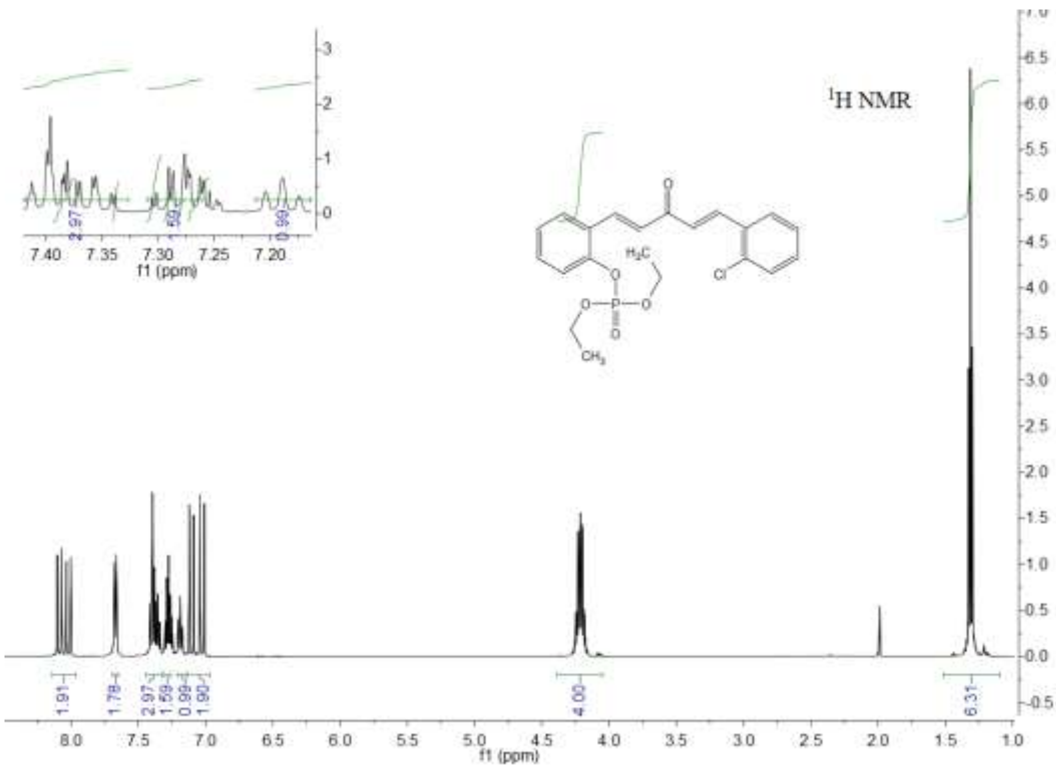

^{1}H NMR of compound **3f**

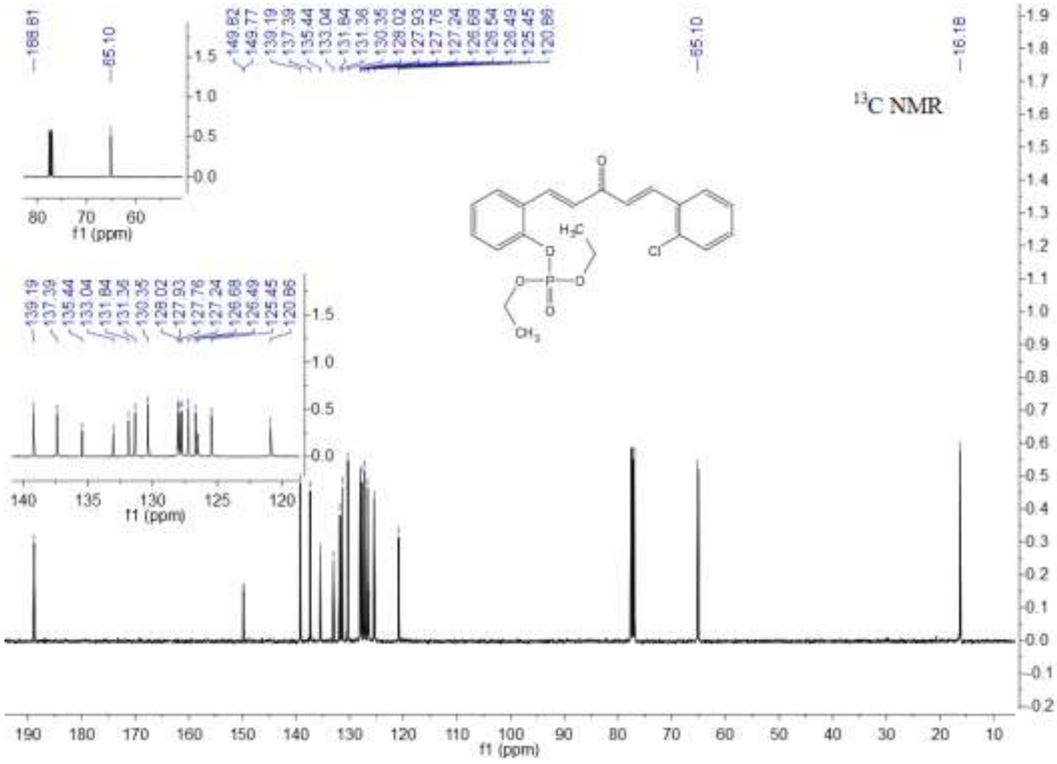

^{13}C NMR of compound **3f**

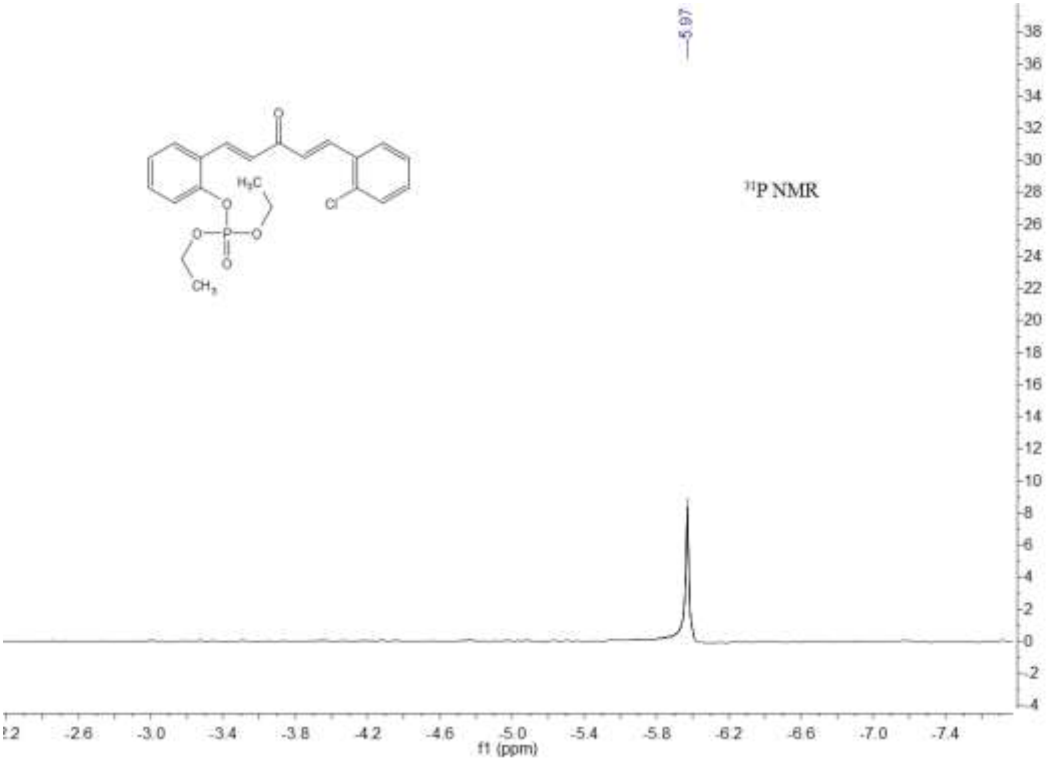

^{31}P NMR of compound **3f**

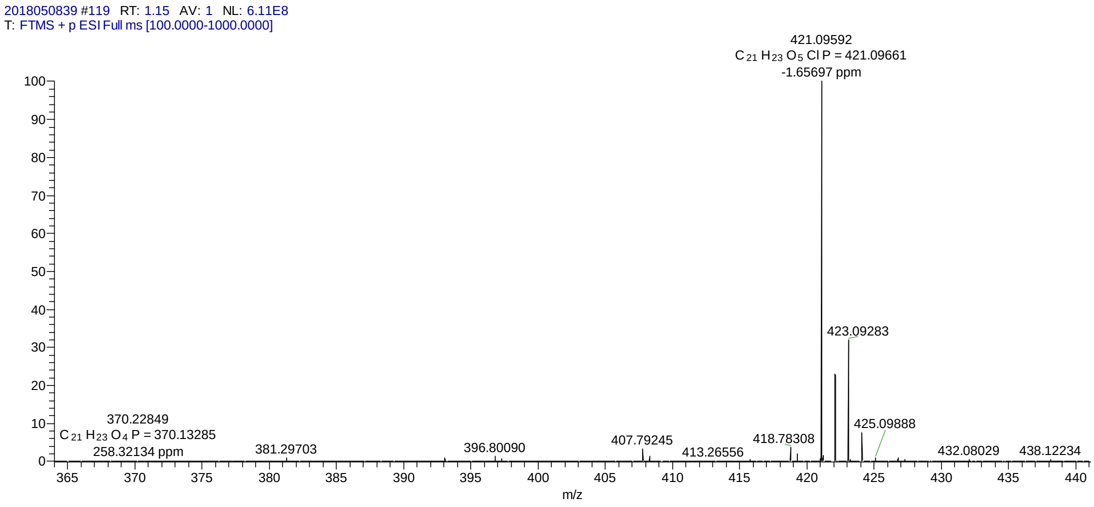

HRMS of compound **3f**

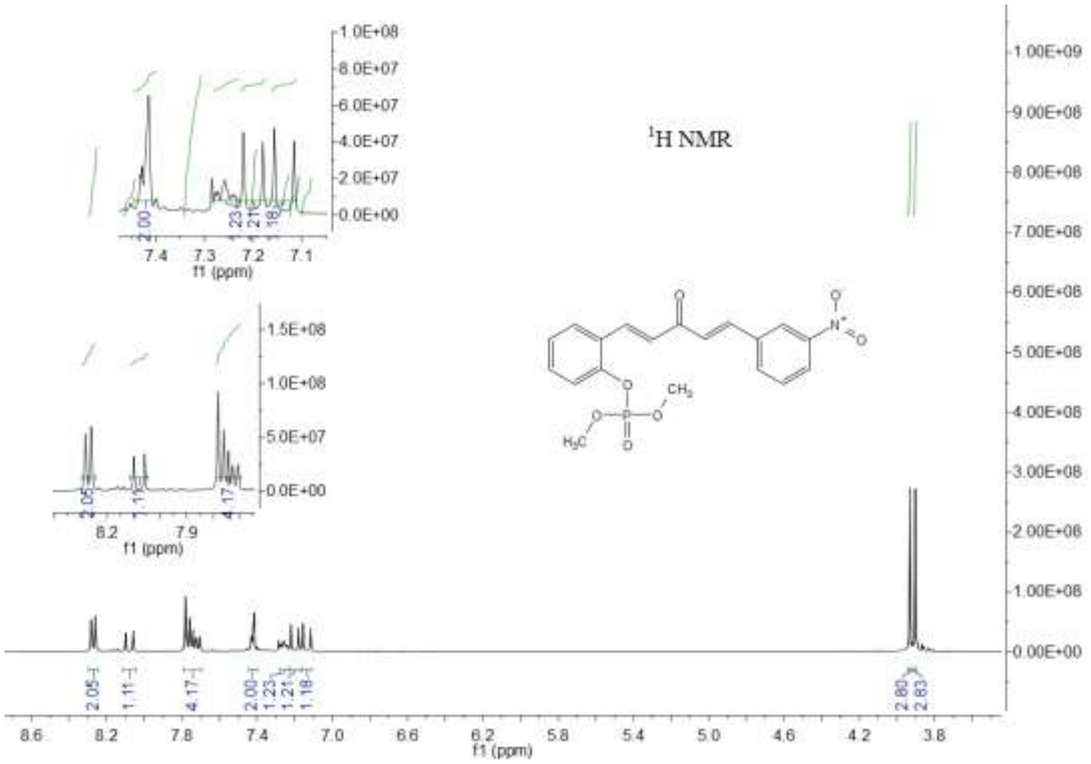

^{1}H NMR of compound **3g**

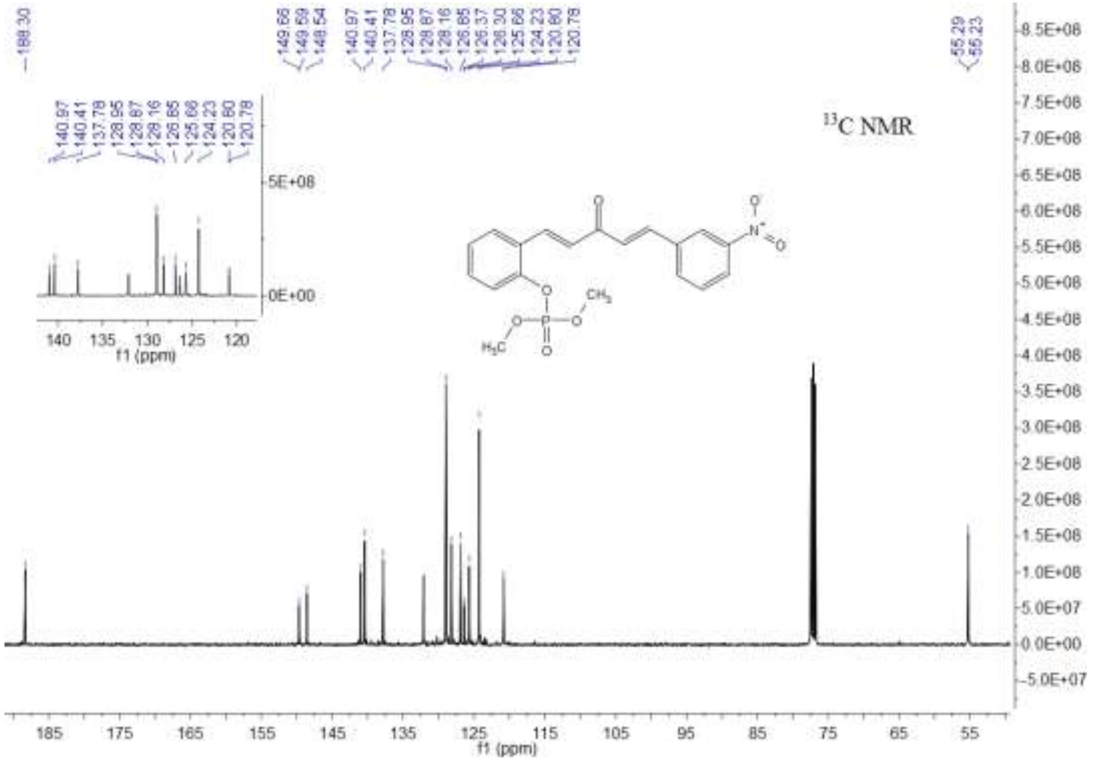

^{13}C NMR of compound **3g**

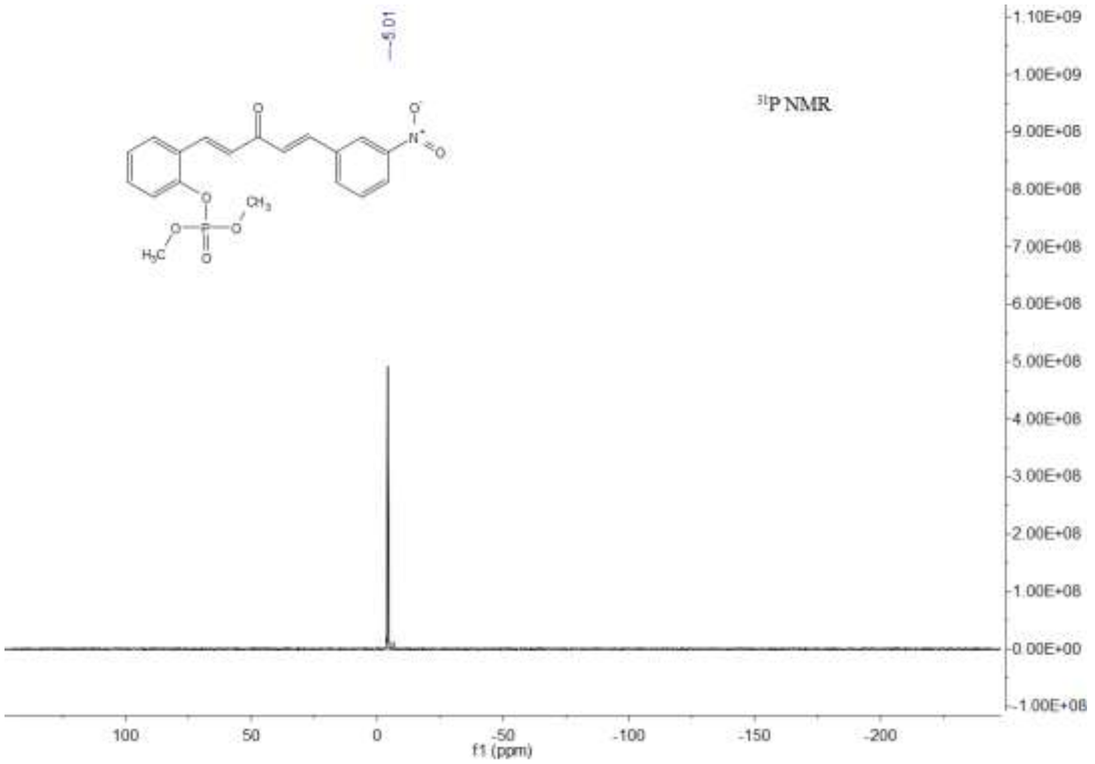

^{31}P NMR of compound **3g**

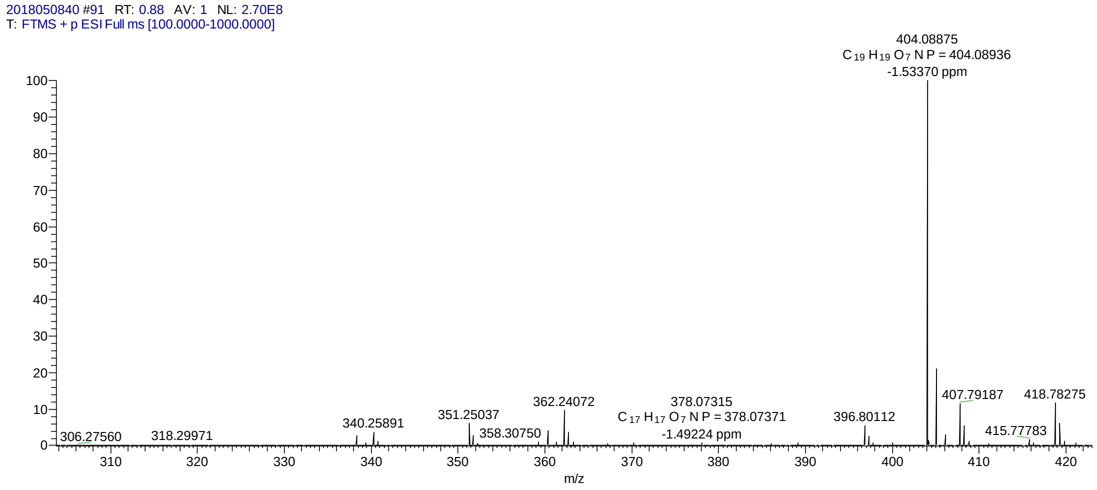

HRMS of compound **3g**

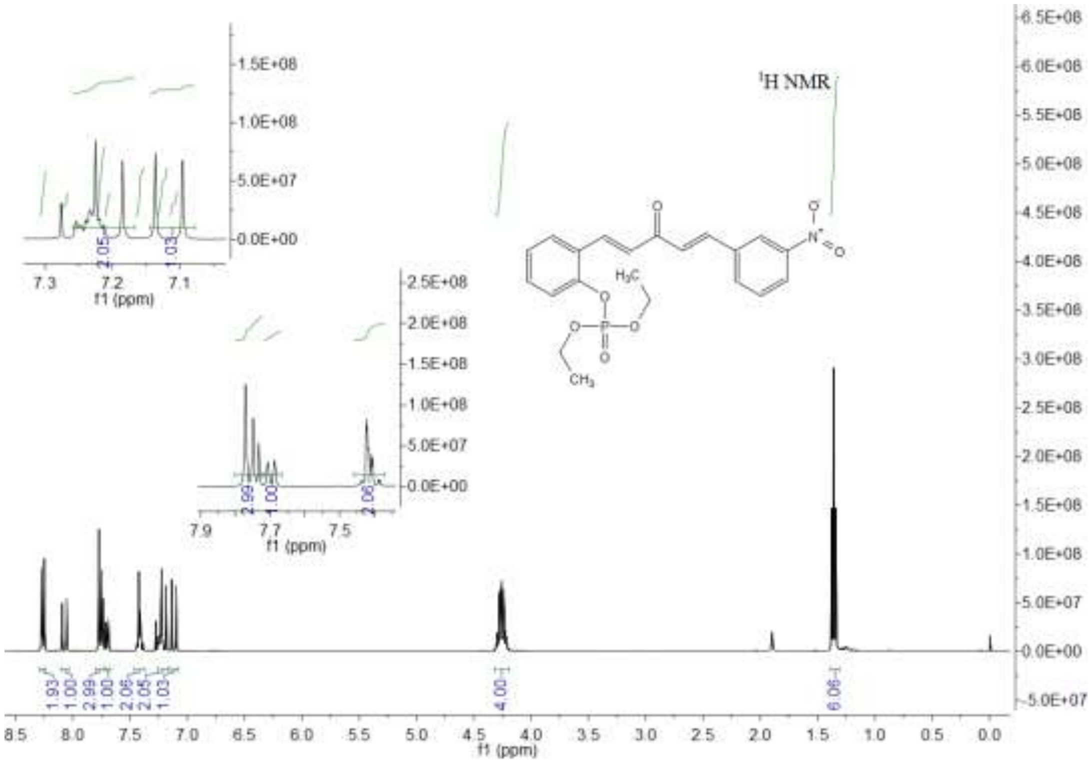

^{1}H NMR of compound **3h**

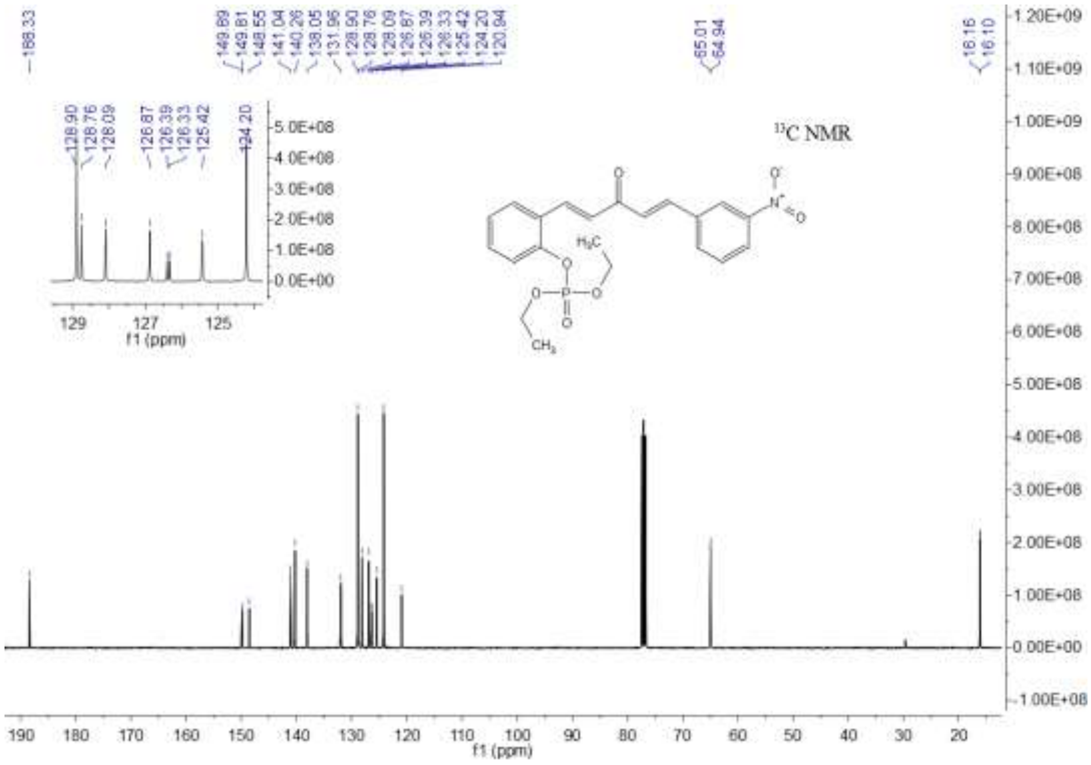

^{13}C NMR of compound **3h**

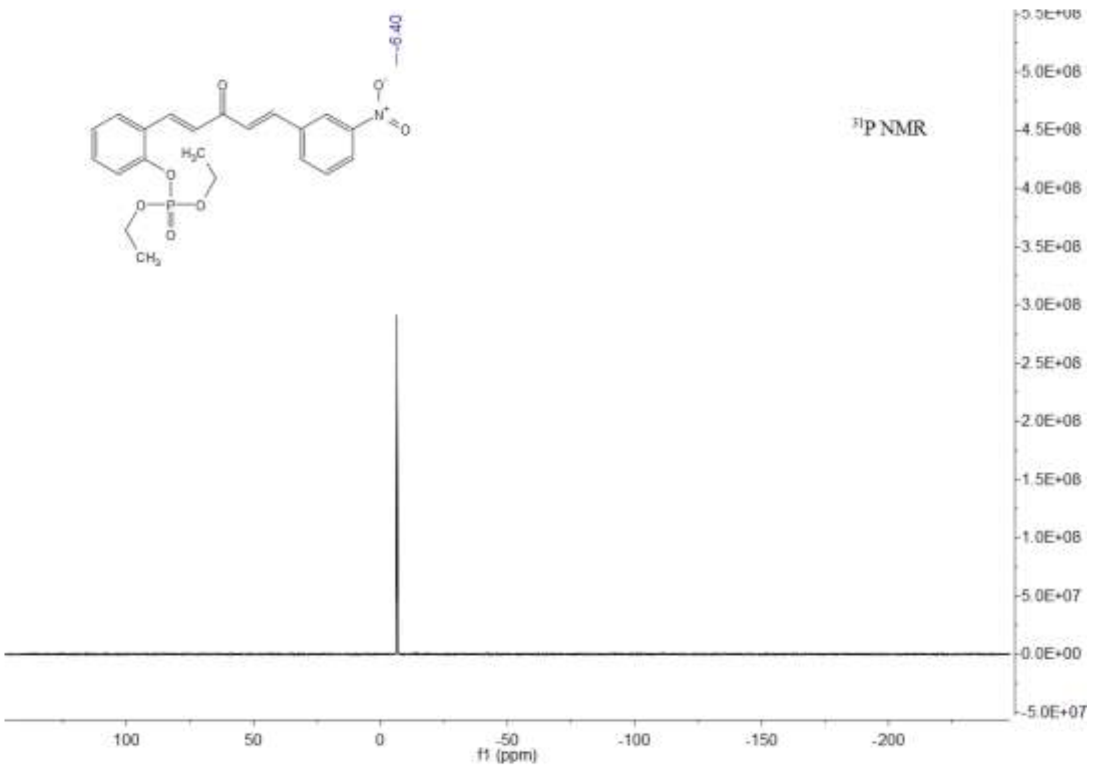

^{31}P NMR of compound **3h**

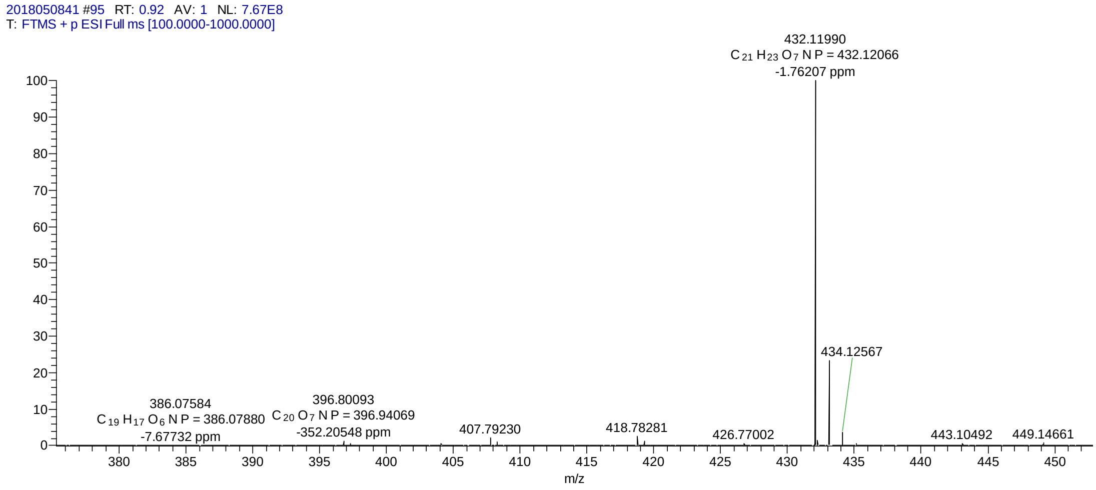

HRMS of compound **3h**

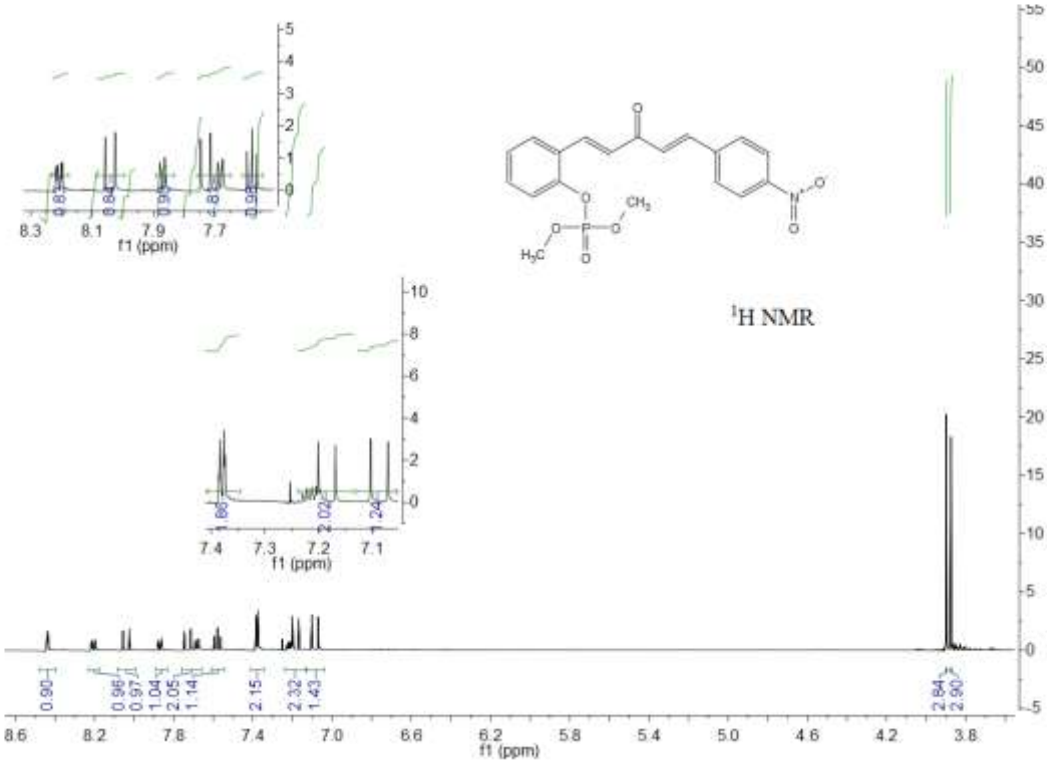

^{1}H NMR of compound **3i**

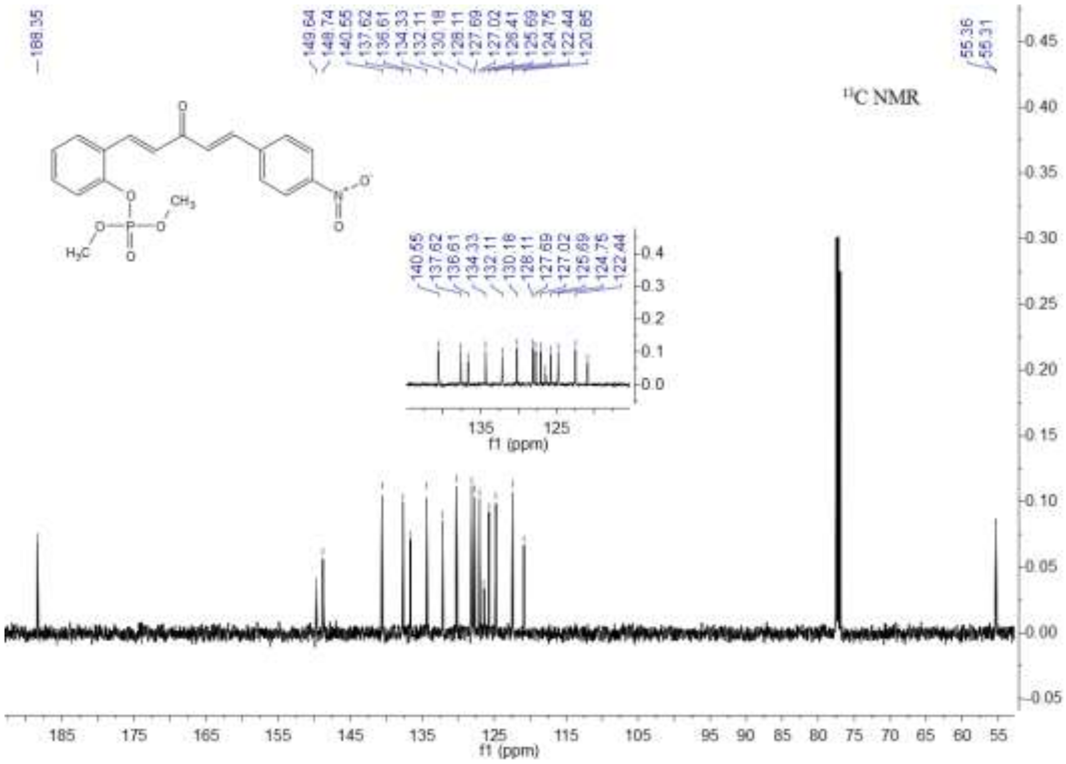

^{13}C NMR of compound **3i**

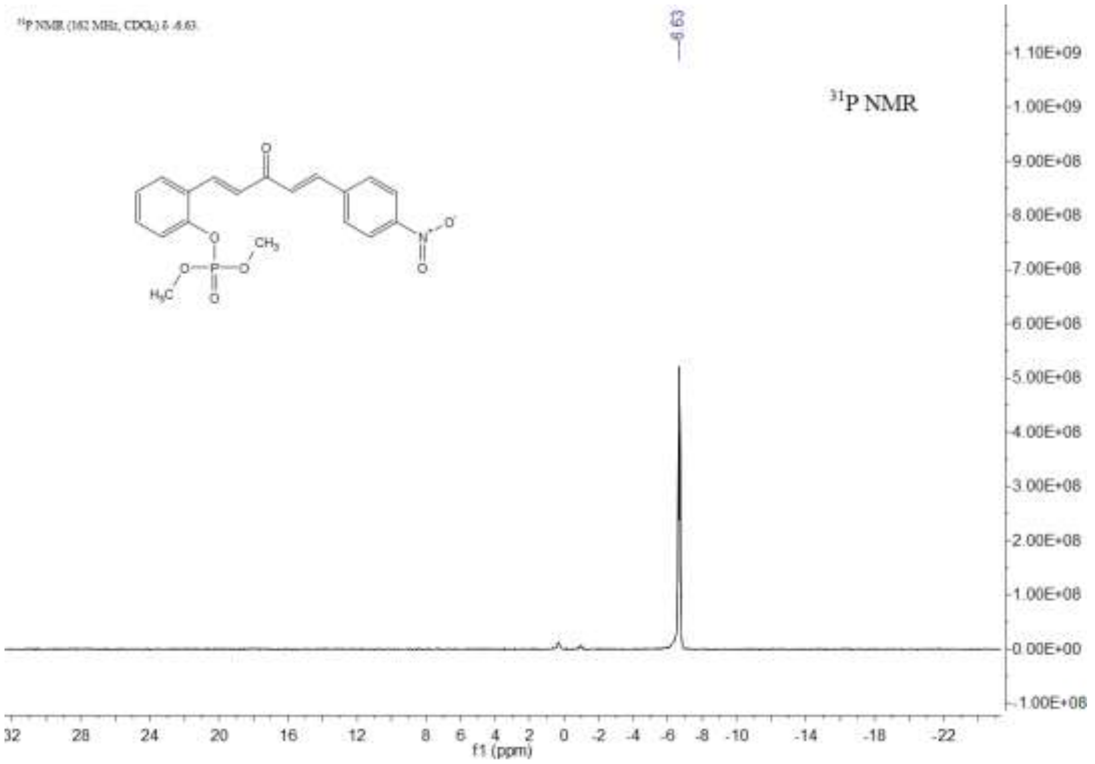

^{31}P NMR of compound **3i**

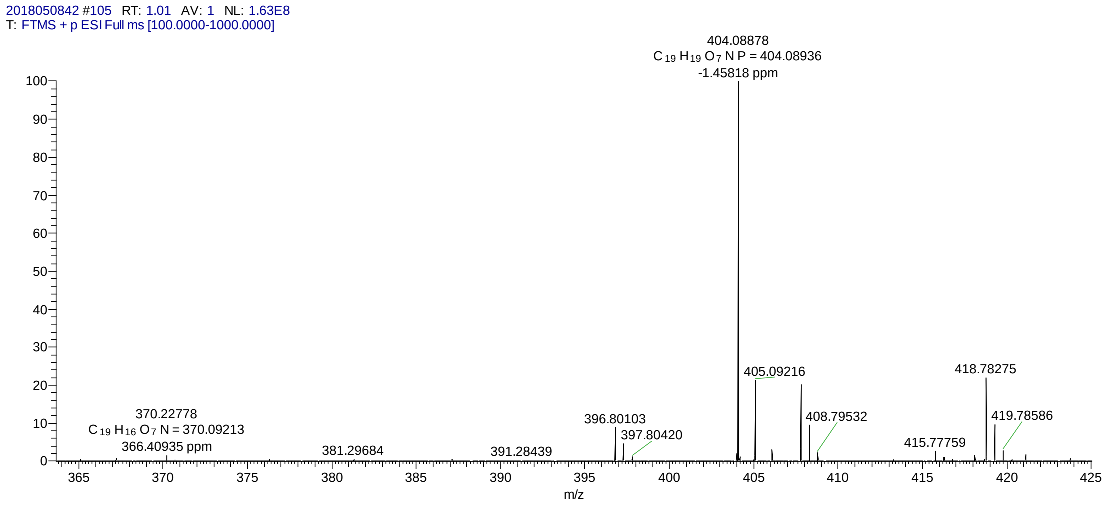

HRMS of compound **3i**

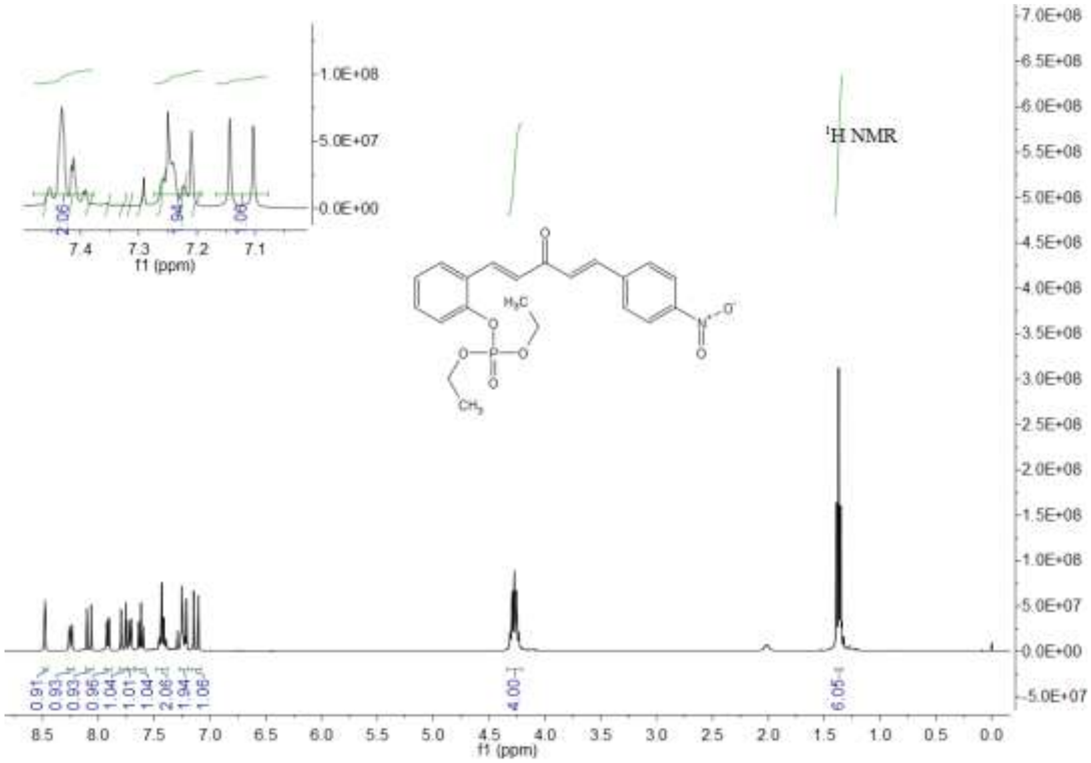

^{1}H NMR of compound **3j**

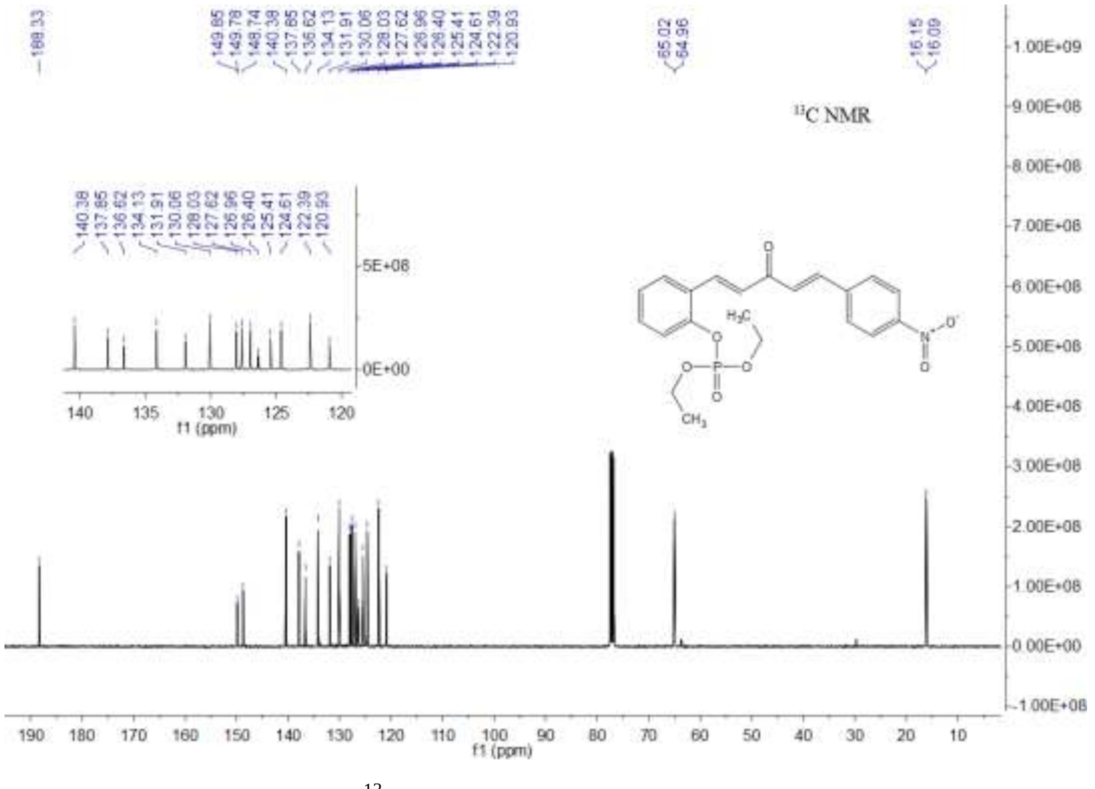

^{13}C NMR of compound **3j**

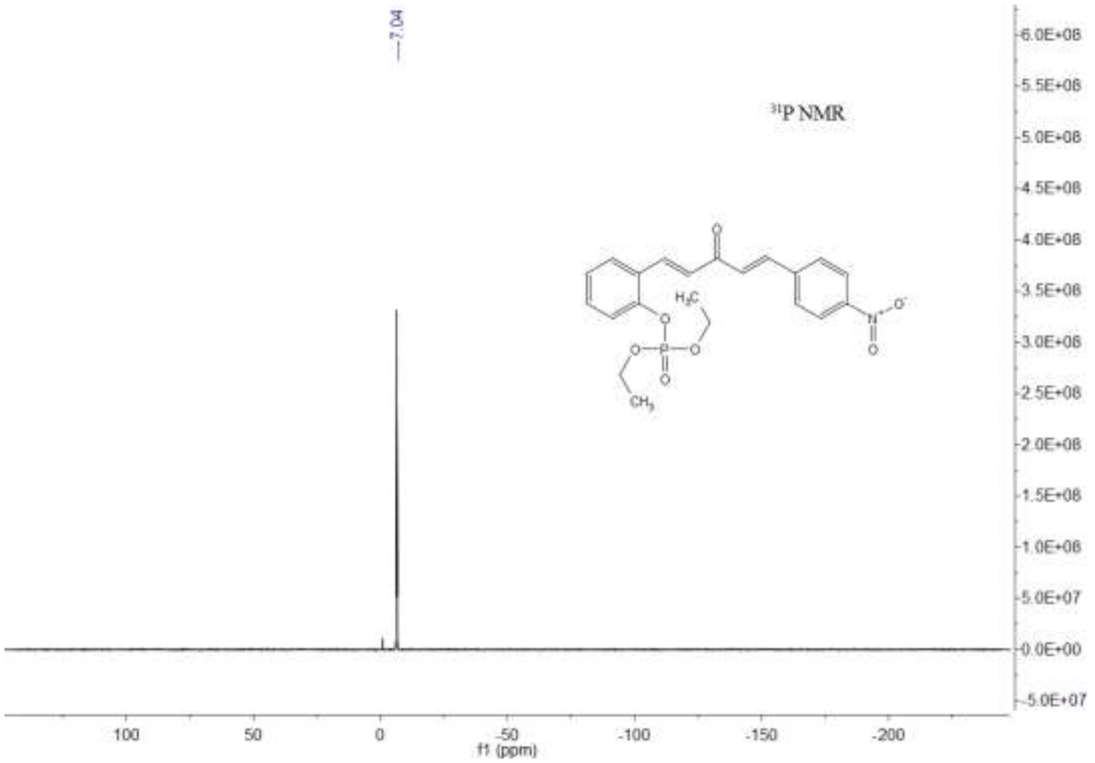

^{31}P NMR of compound **3j**

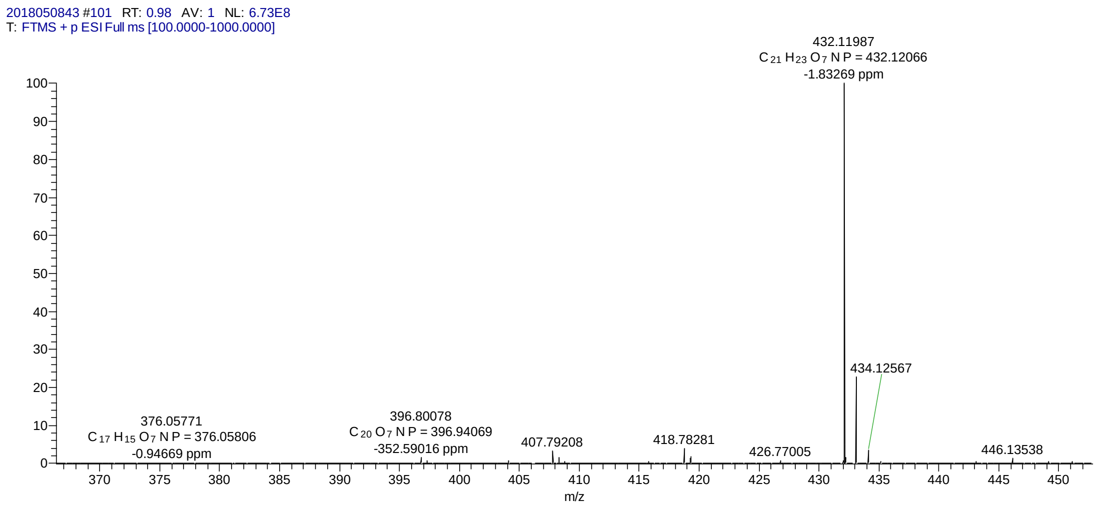

HRMS of compound **3j**

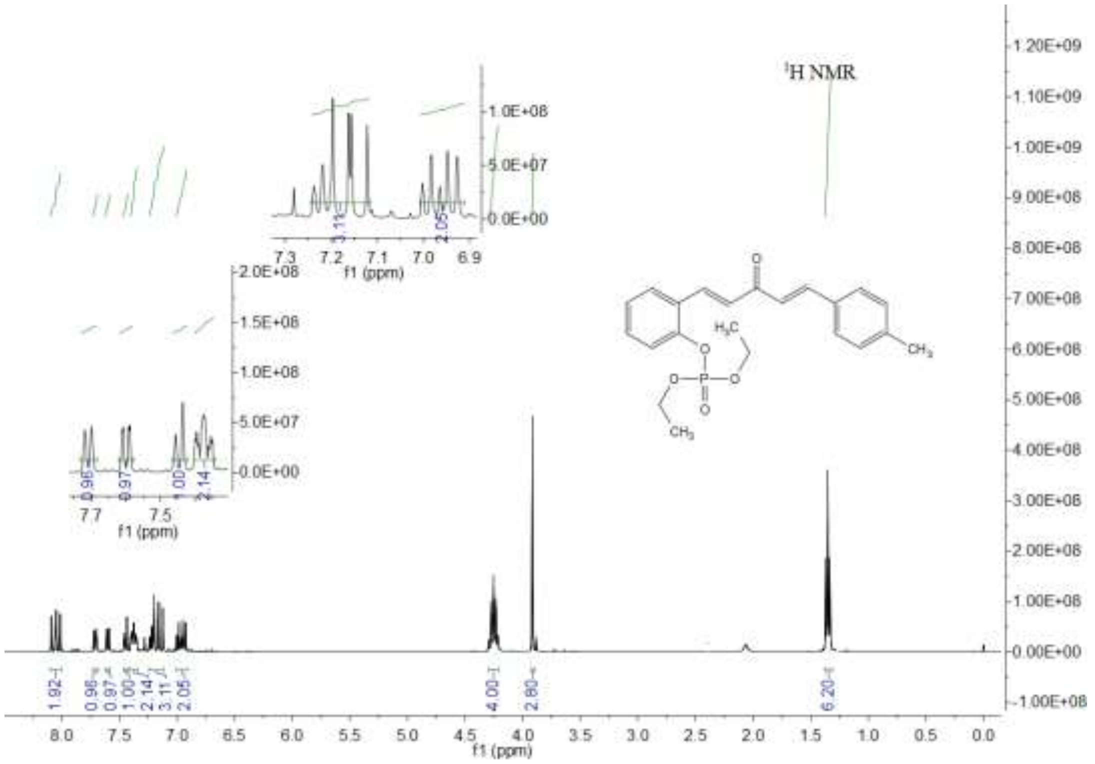

^{1}H NMR of compound **3k**

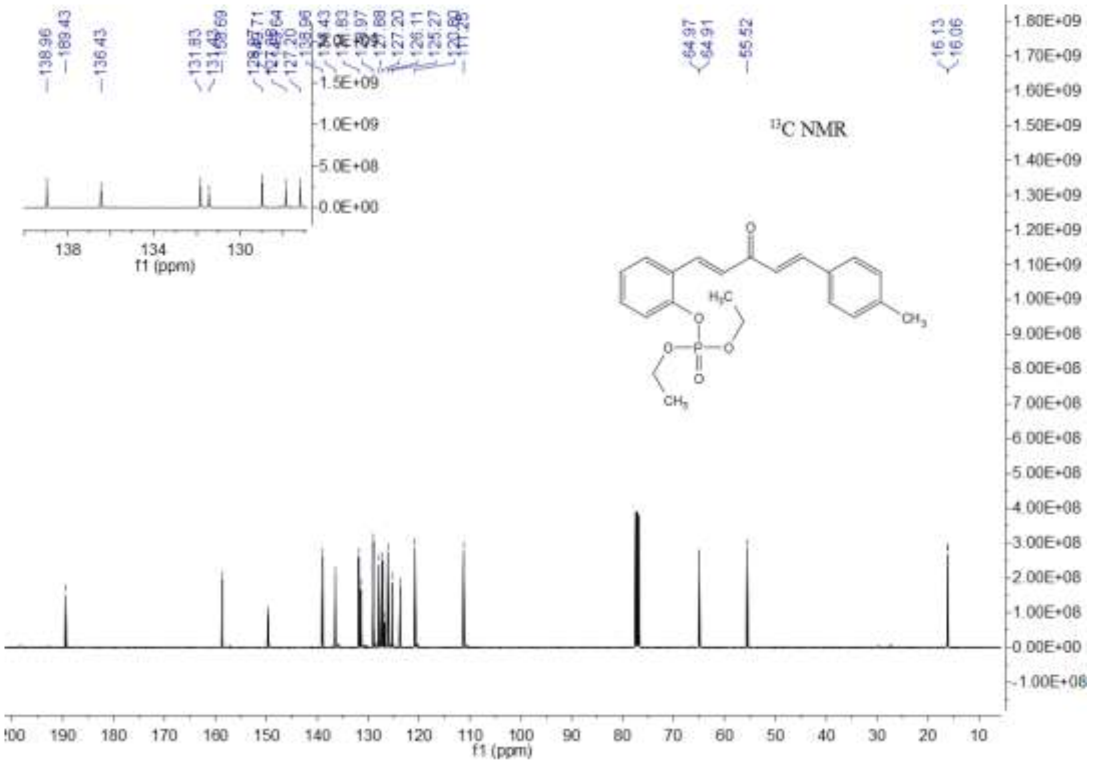

^{13}C NMR of compound **3k**

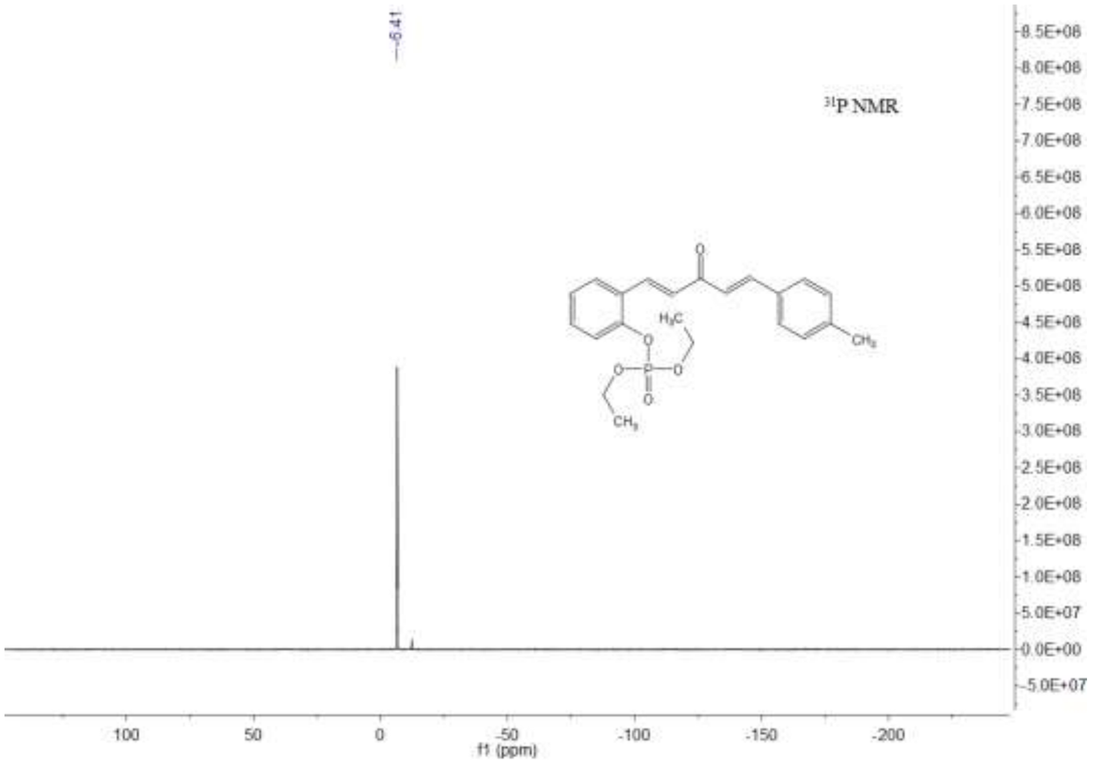

^{31}P NMR of compound **3k**

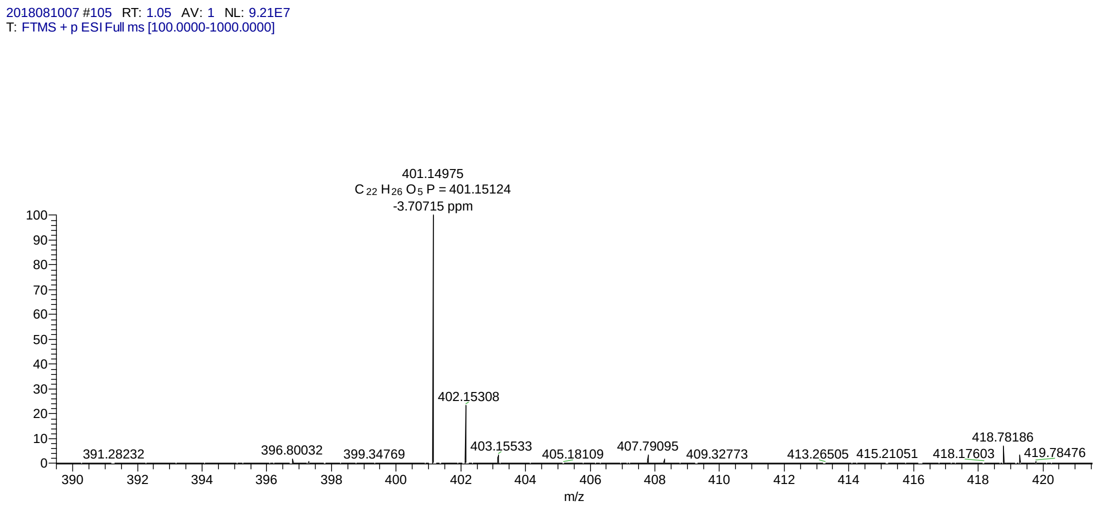

HRMS of compound **3k**

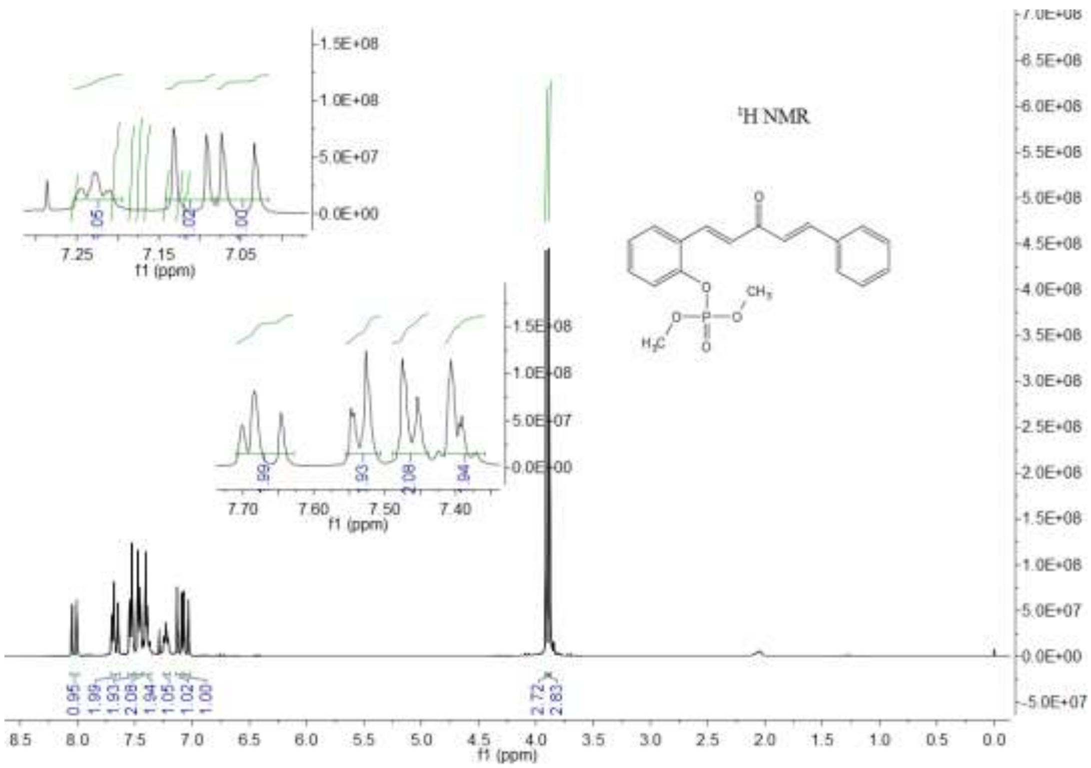

^{1}H NMR of compound **3l**

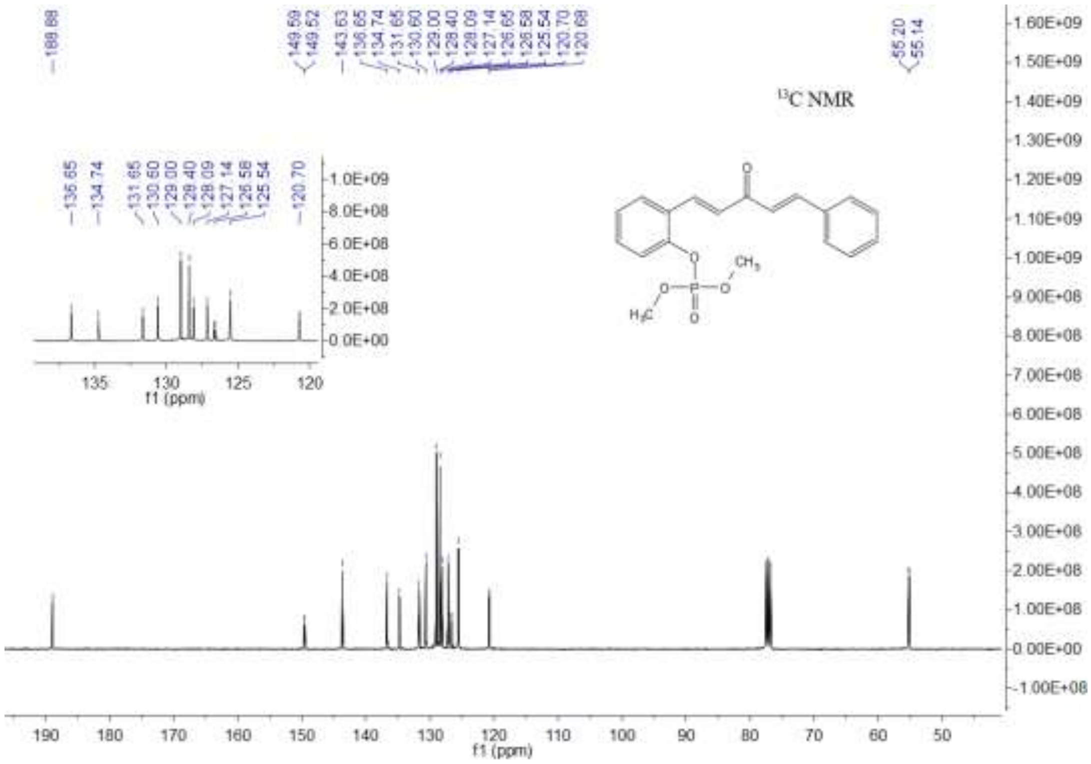

^{13}C NMR of compound **3l**

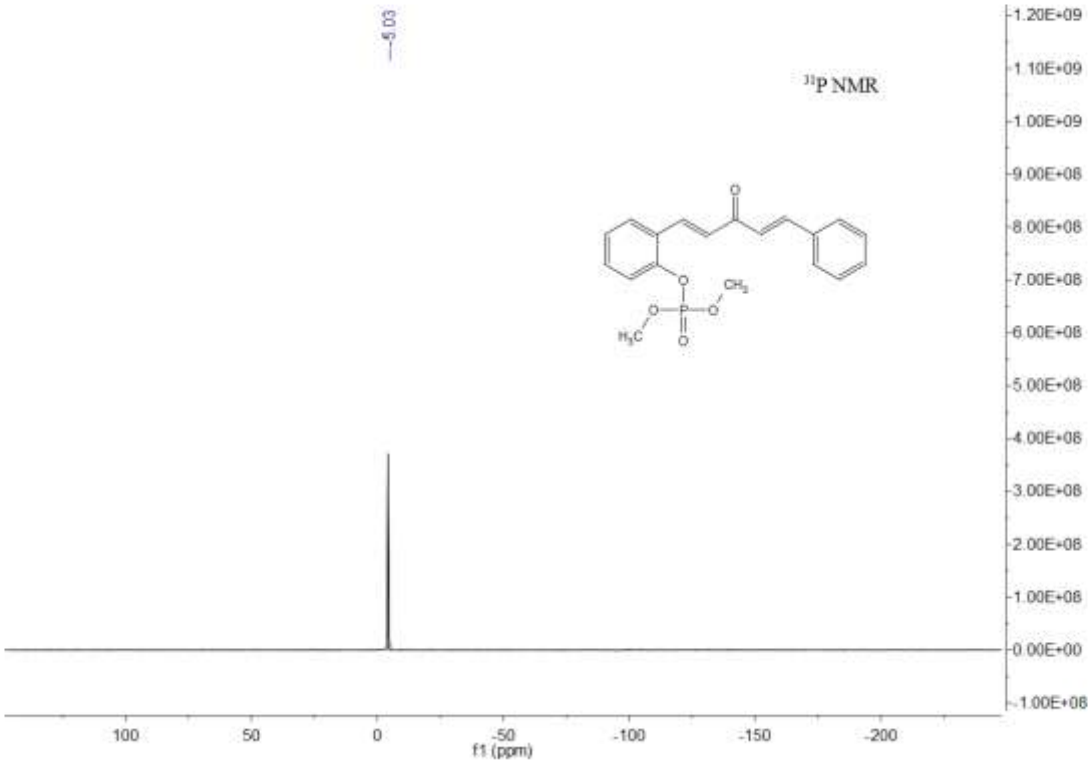

^{31}P NMR of compound **3l**

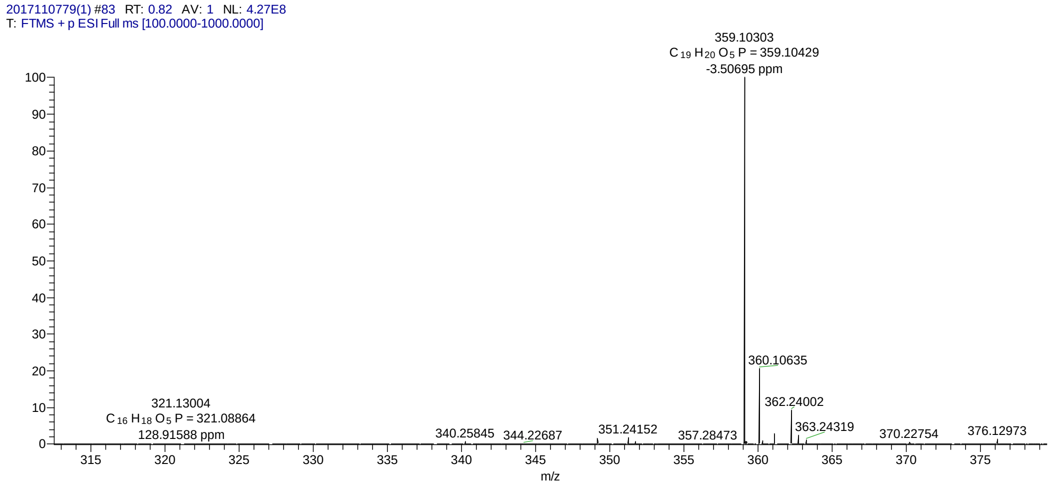

HRMS of compound **3l**

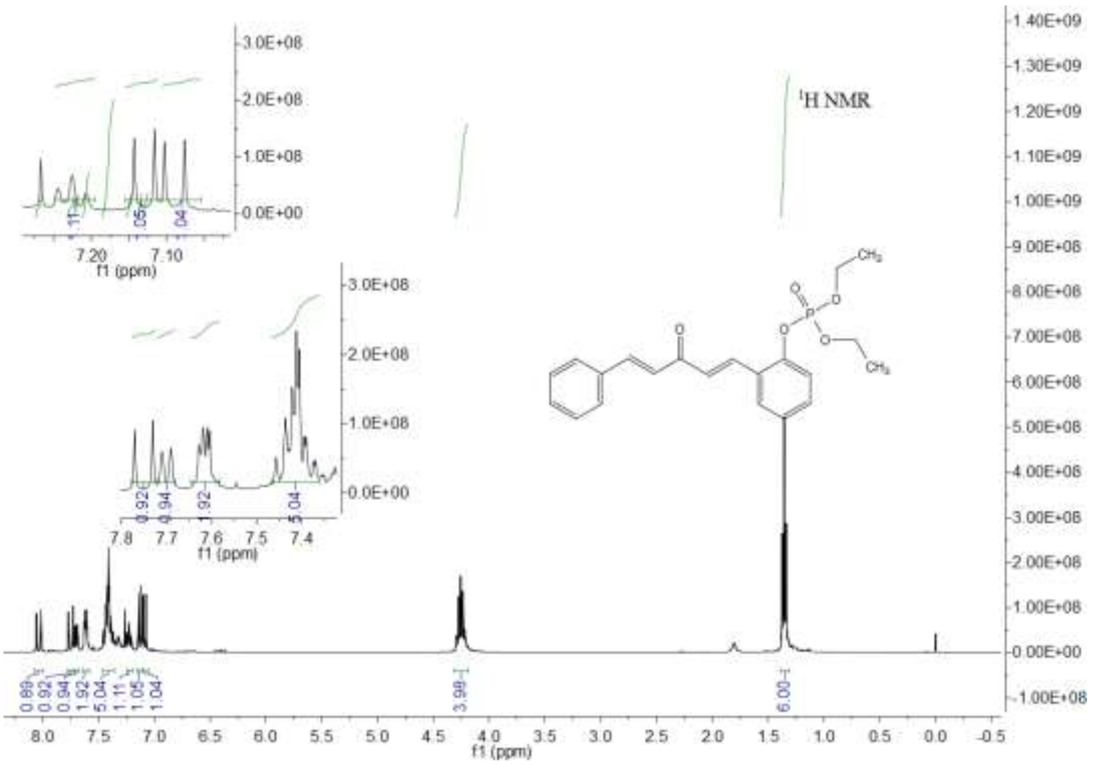

^{1}H NMR of compound **3m**

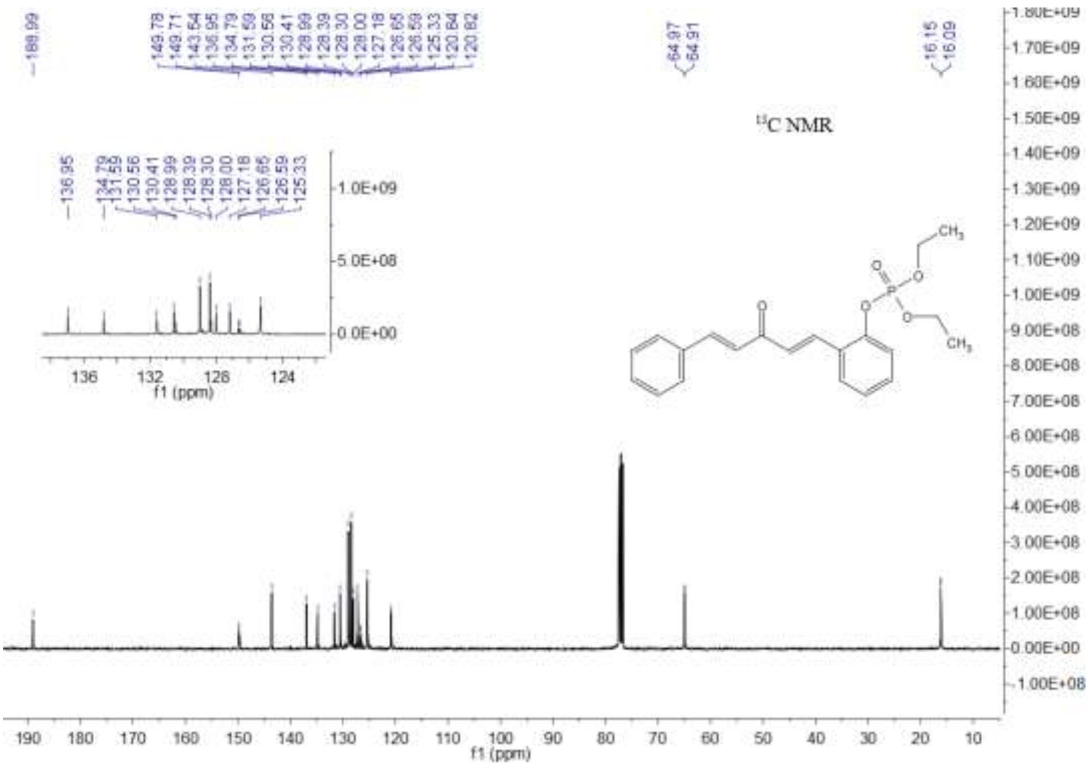

^{13}C NMR of compound **3m**

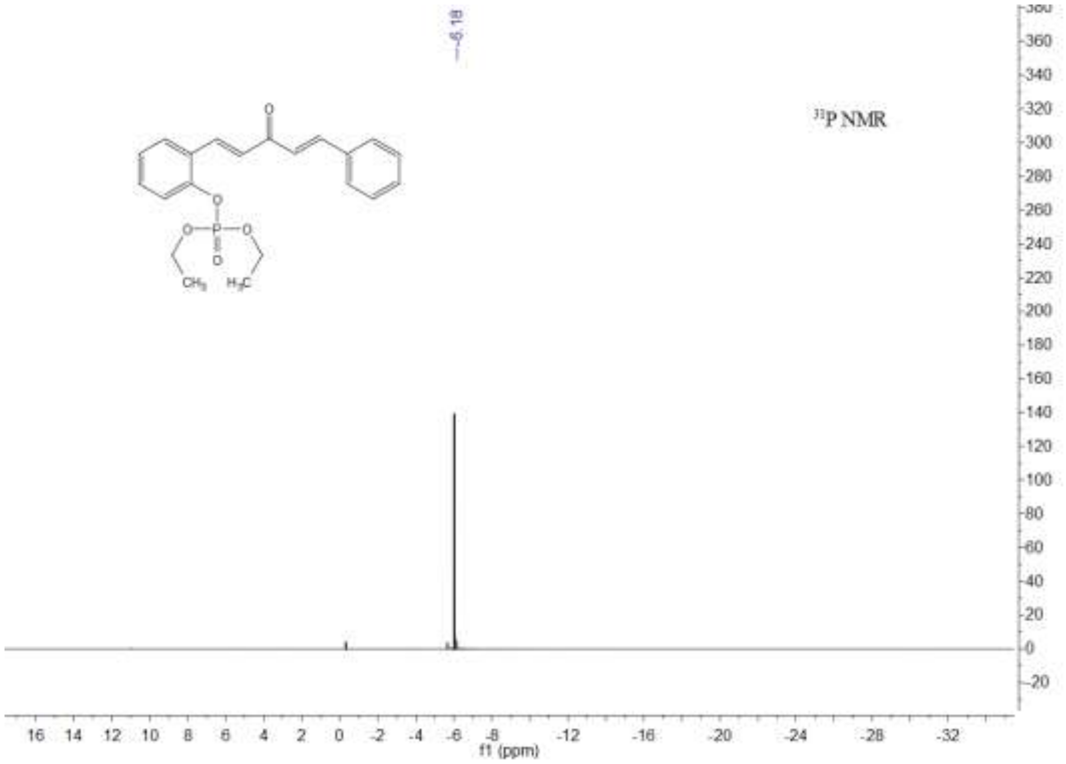

^{31}P NMR of compound **3m**

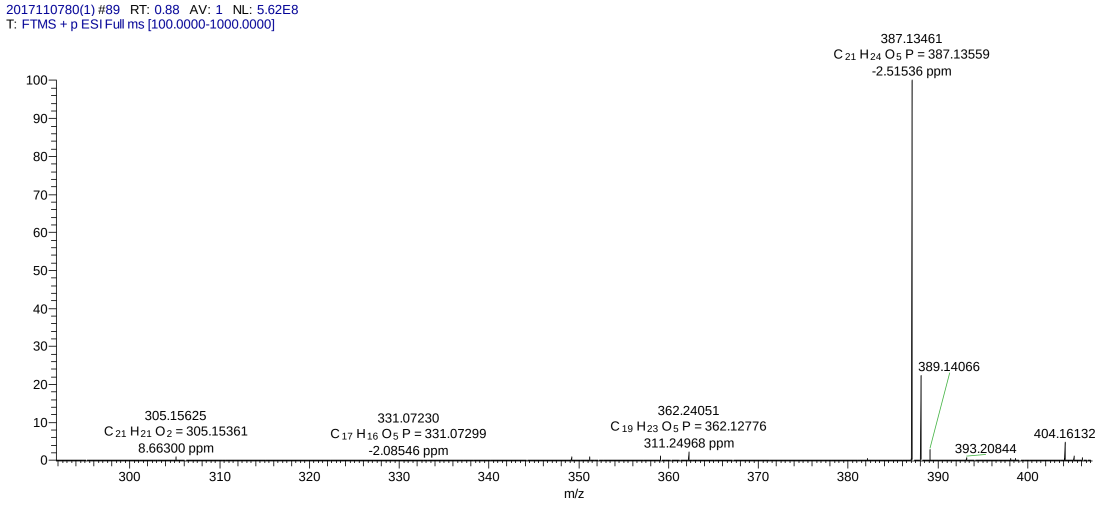

HRMS of compound **3m**

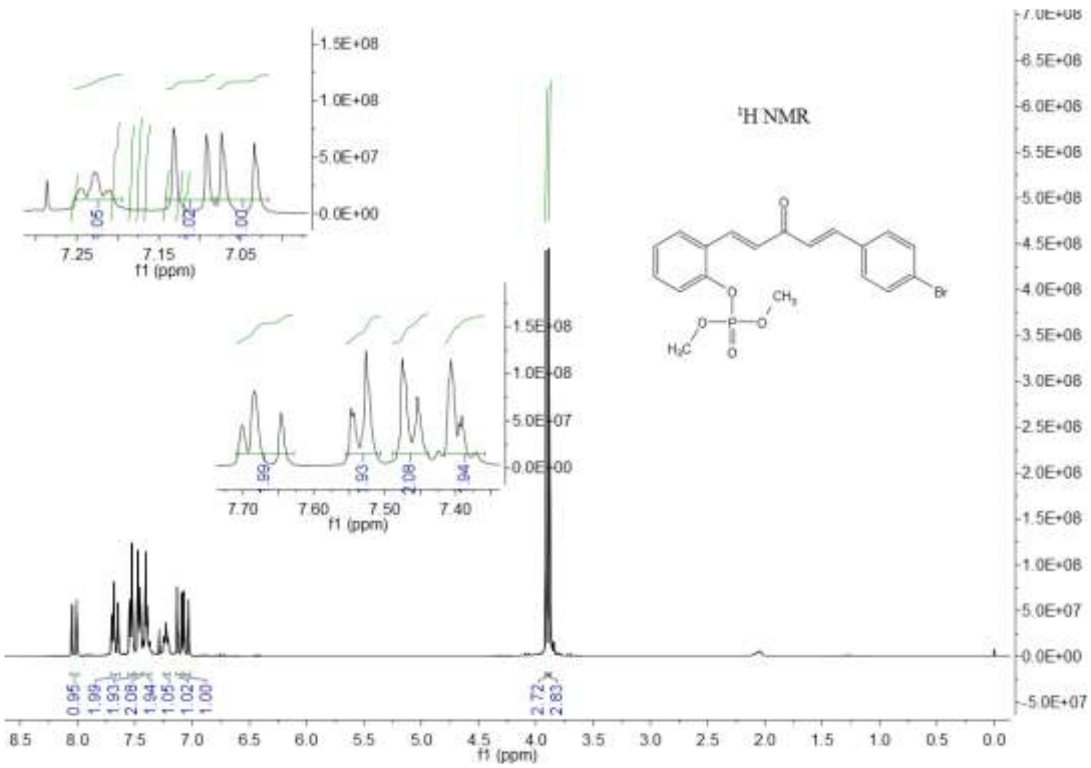

^{1}H NMR of compound **3n**

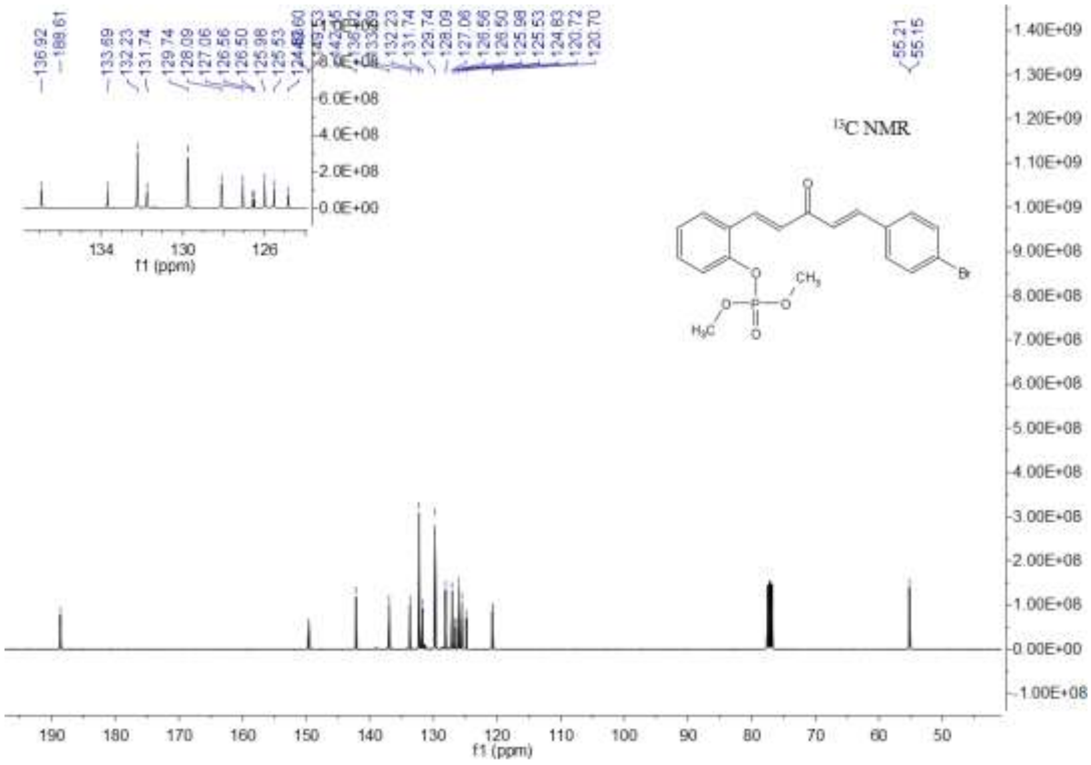

^{13}C NMR of compound **3n**

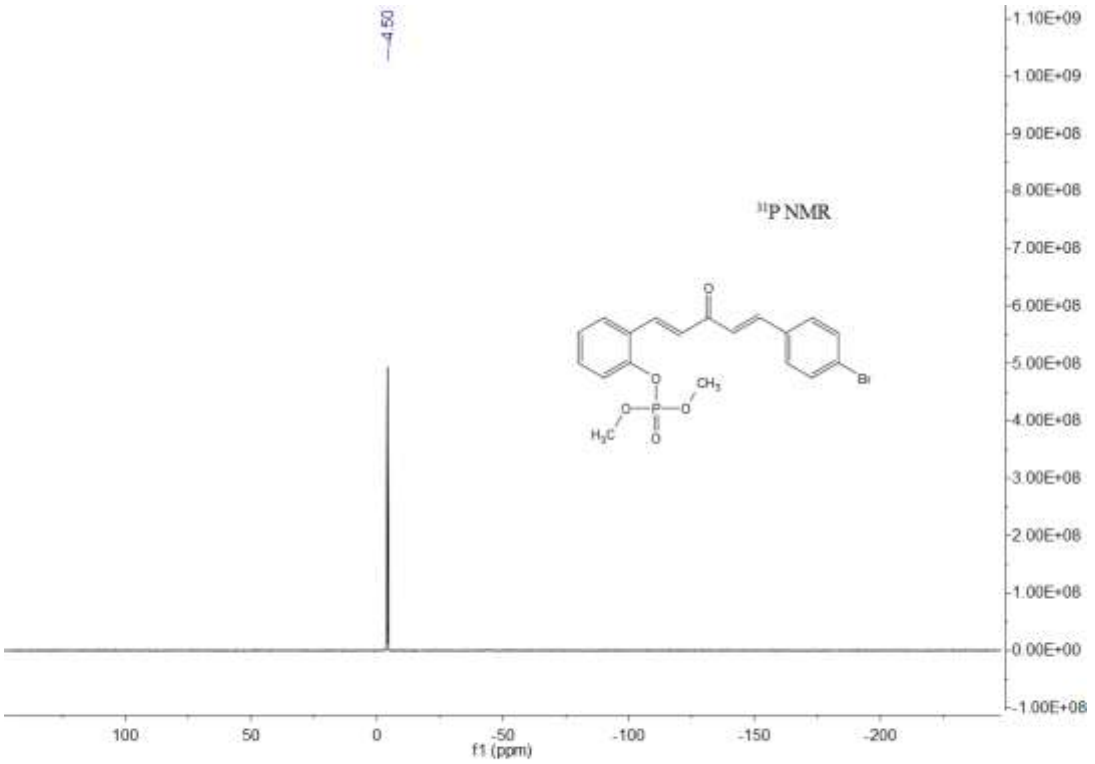

^{31}P NMR of compound **3n**

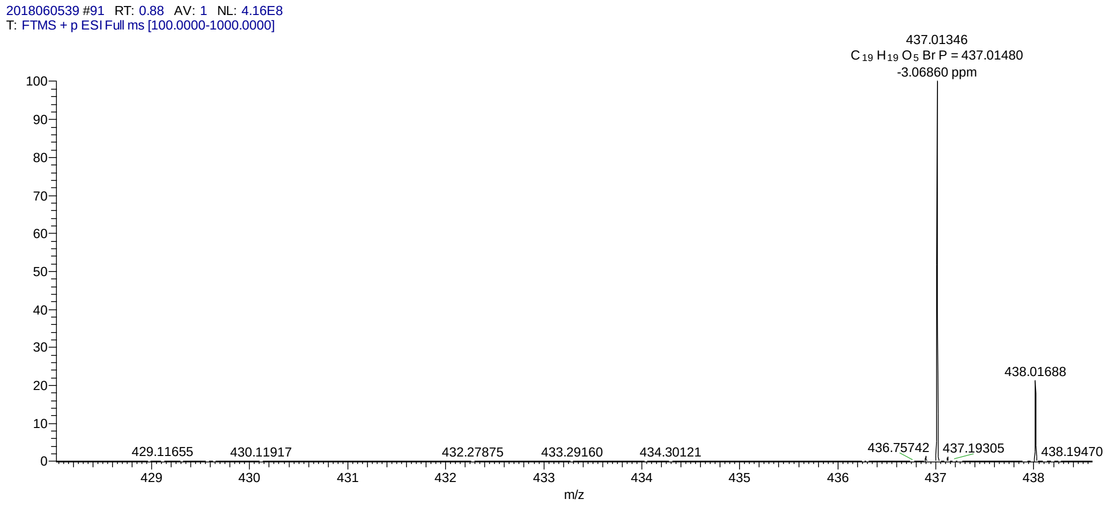

HRMS of compound **3n**

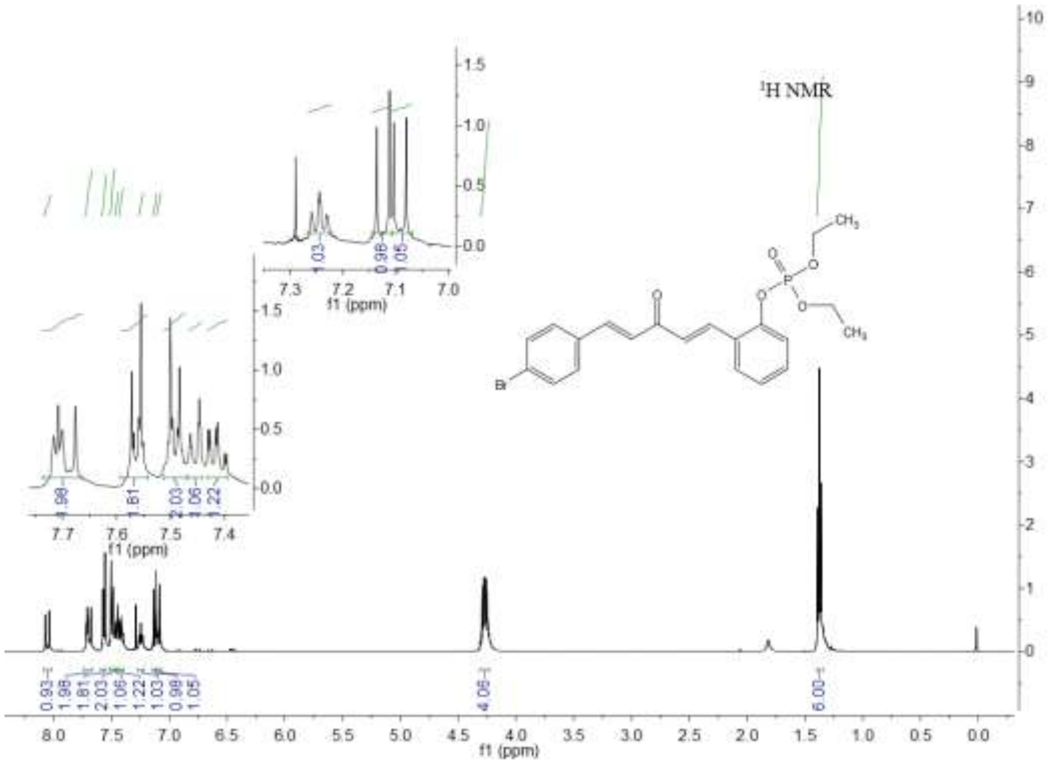

^{1}H NMR of compound **3o**

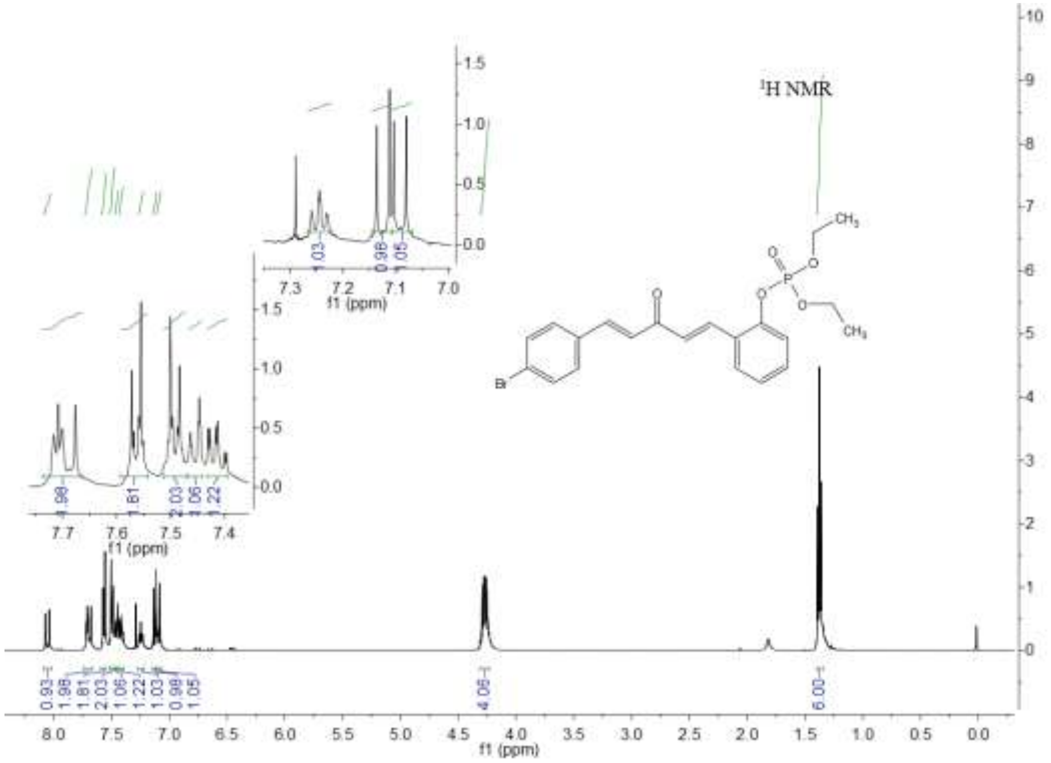

^{13}C NMR of compound **3o**

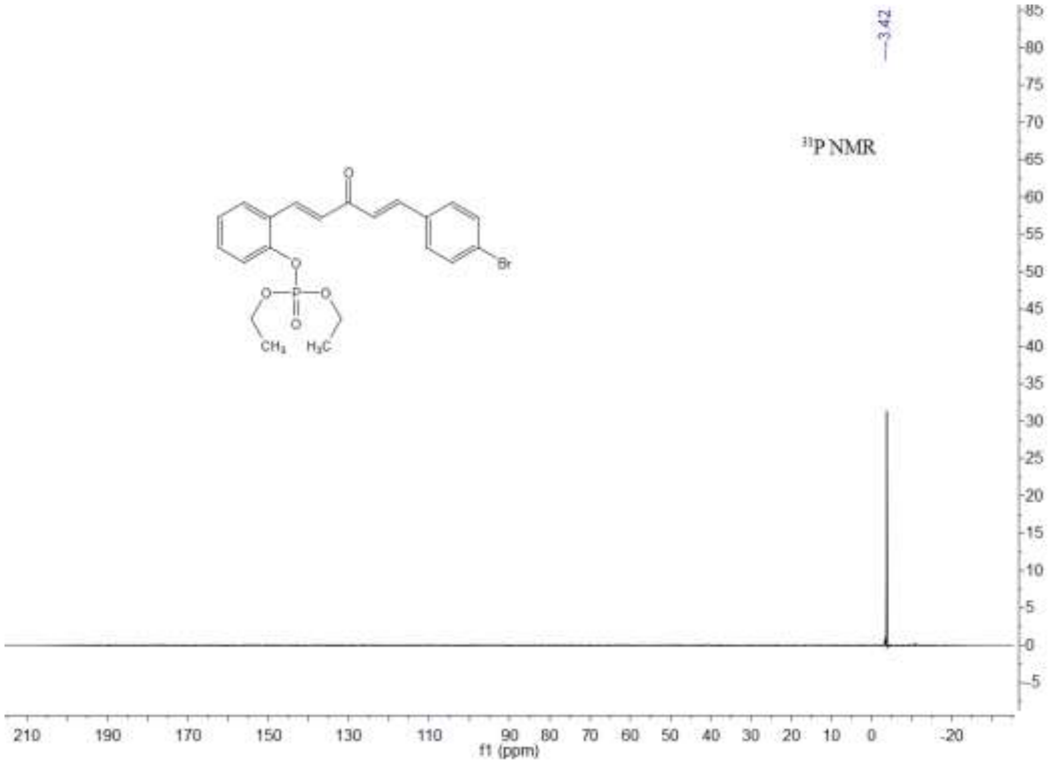

^{31}P NMR of compound **3o**

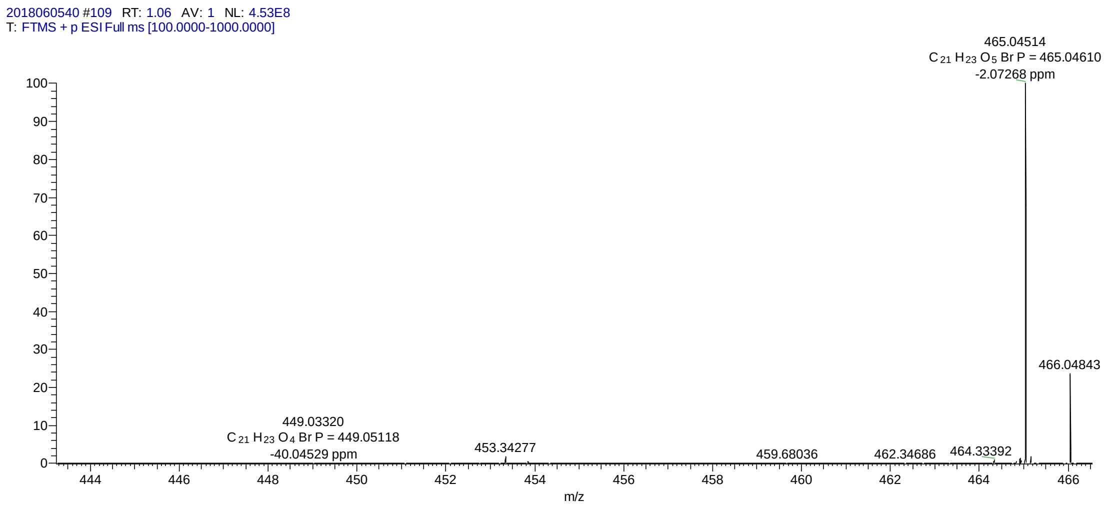

HRMS of compound **3o**

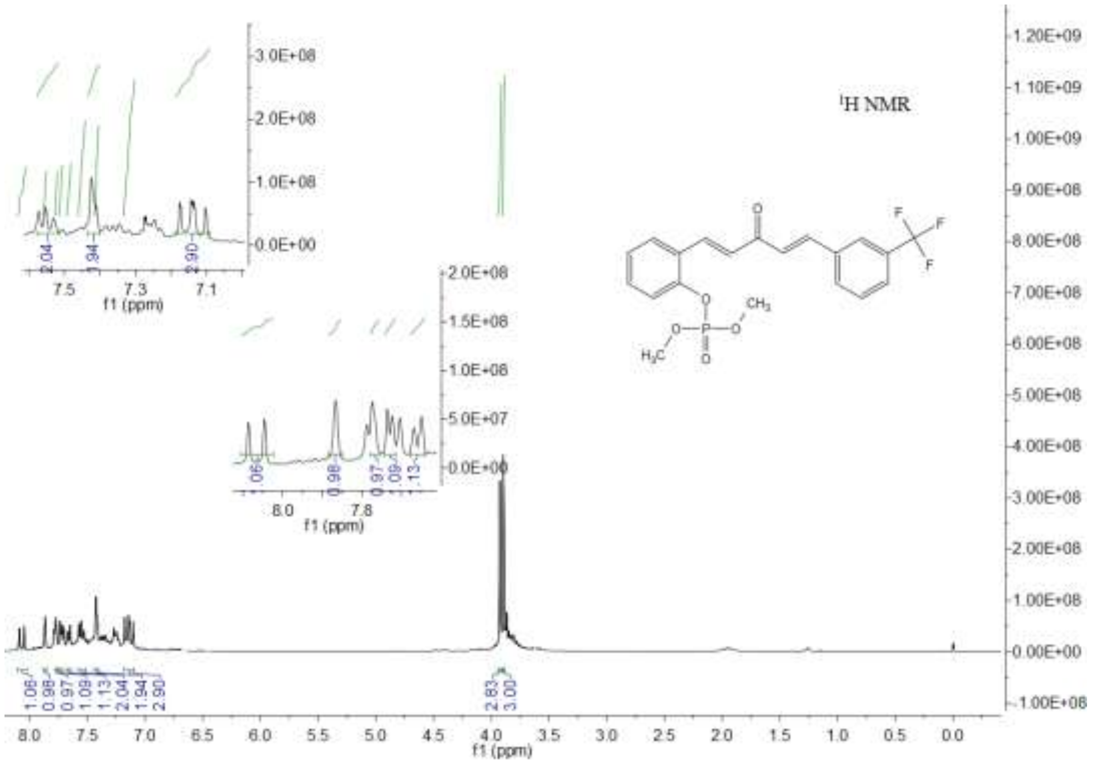

^{1}H NMR of compound **3p**

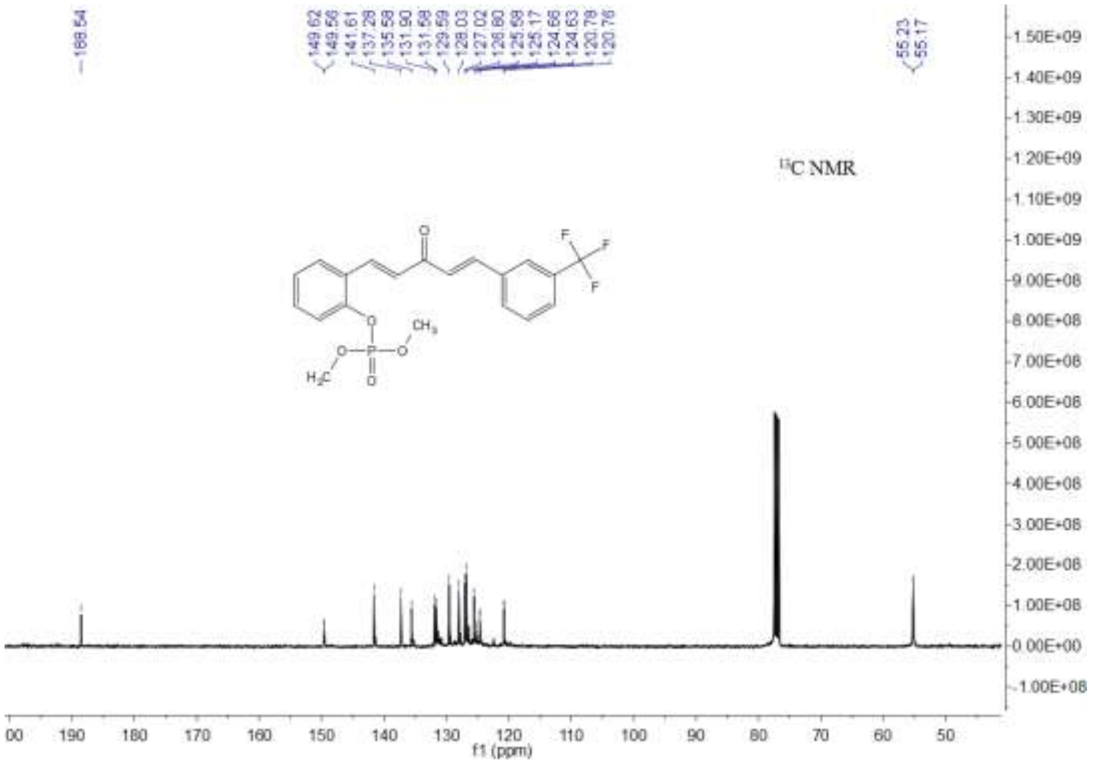

^{13}C NMR of compound **3p**

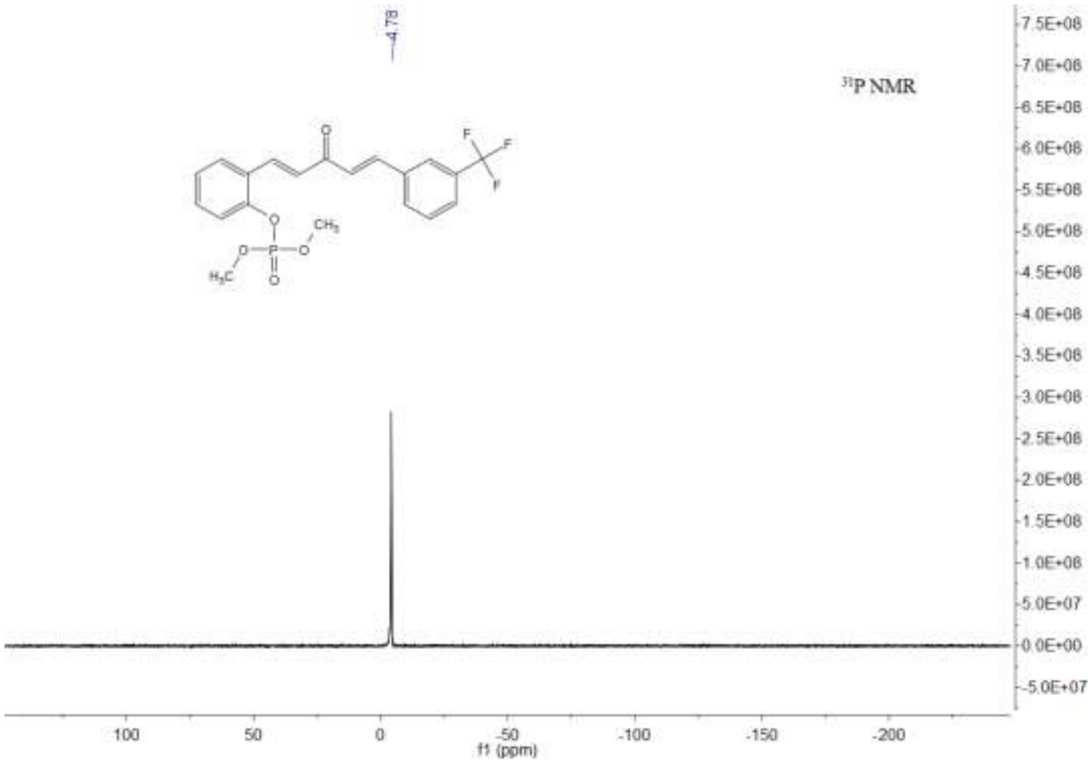

^{31}P NMR of compound **3p**

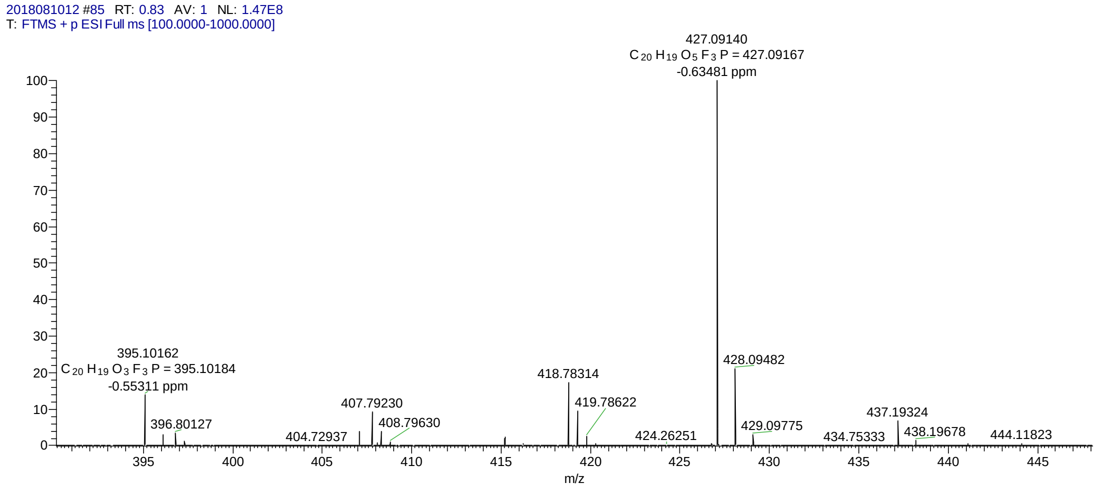

HRMS of compound **3p**

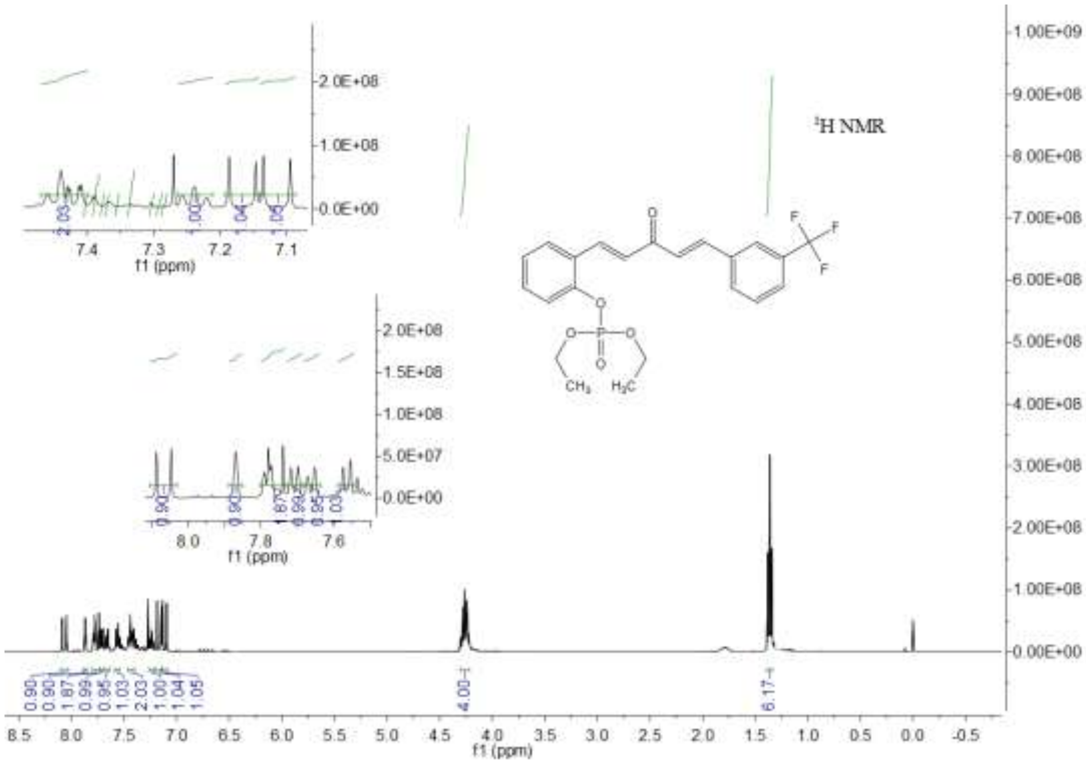

^{1}H NMR of compound **3q**

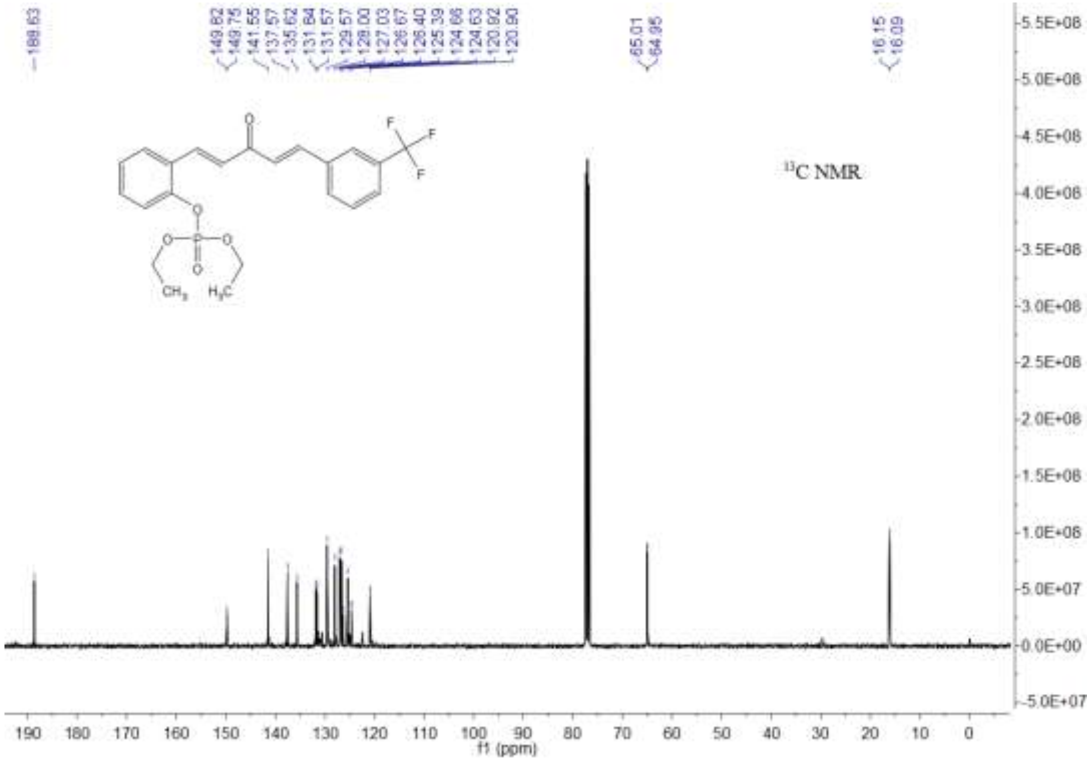

^{13}C NMR of compound **3q**

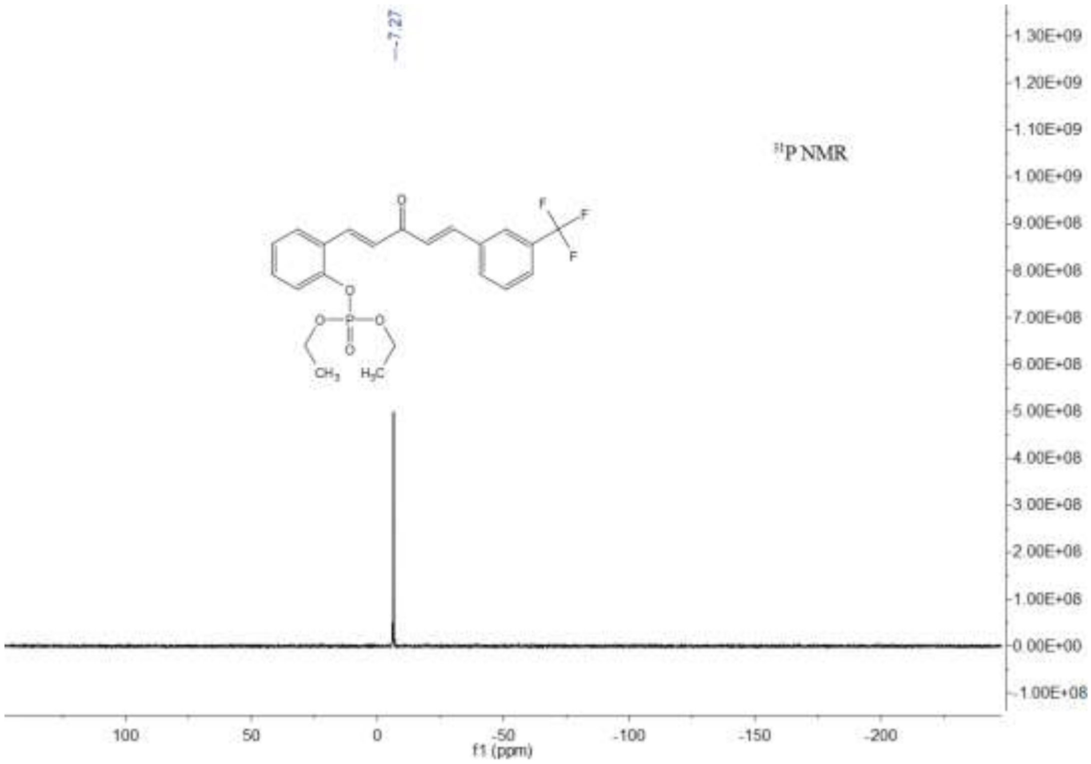

^{31}P NMR of compound **3q**

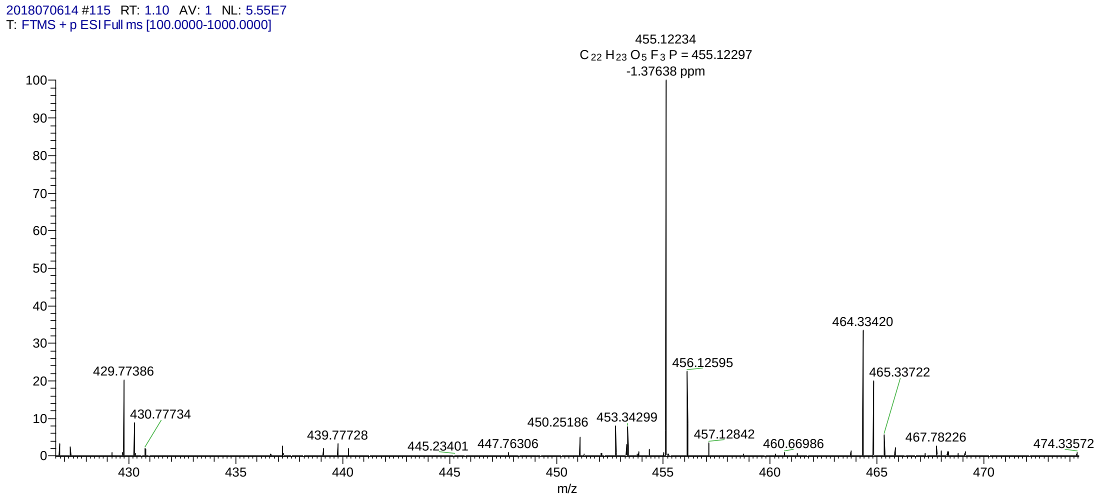

HRMS of compound **3q**

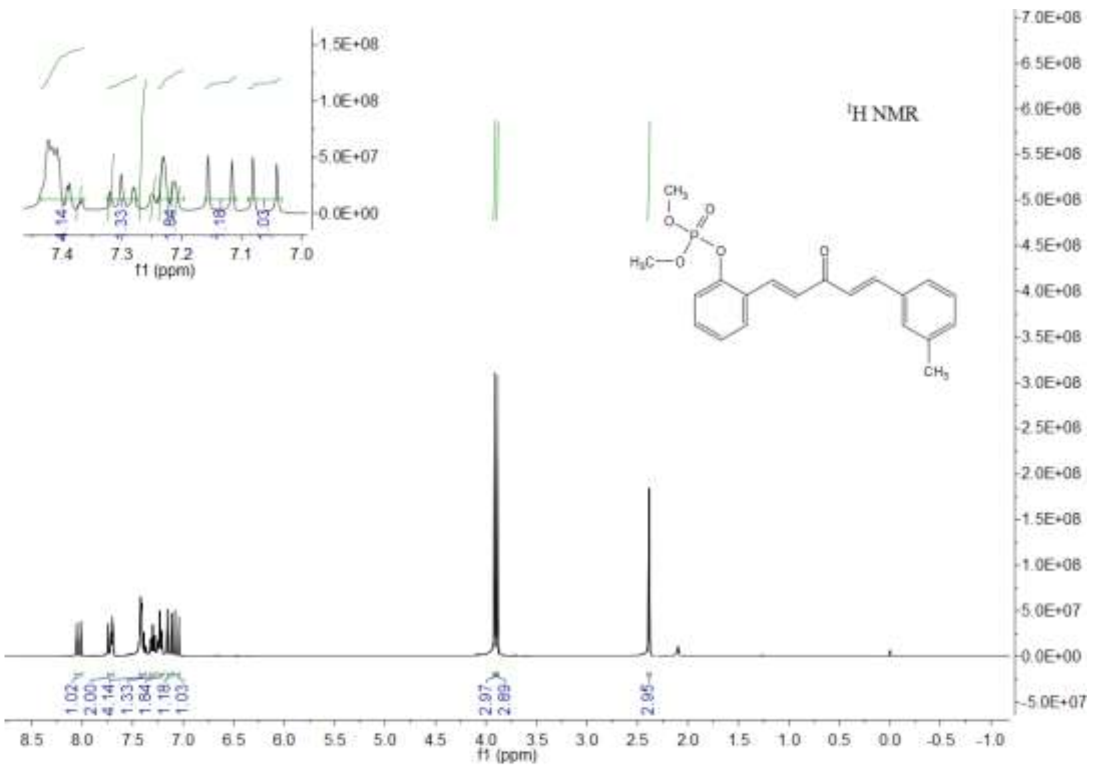

^{1}H NMR of compound **3r**

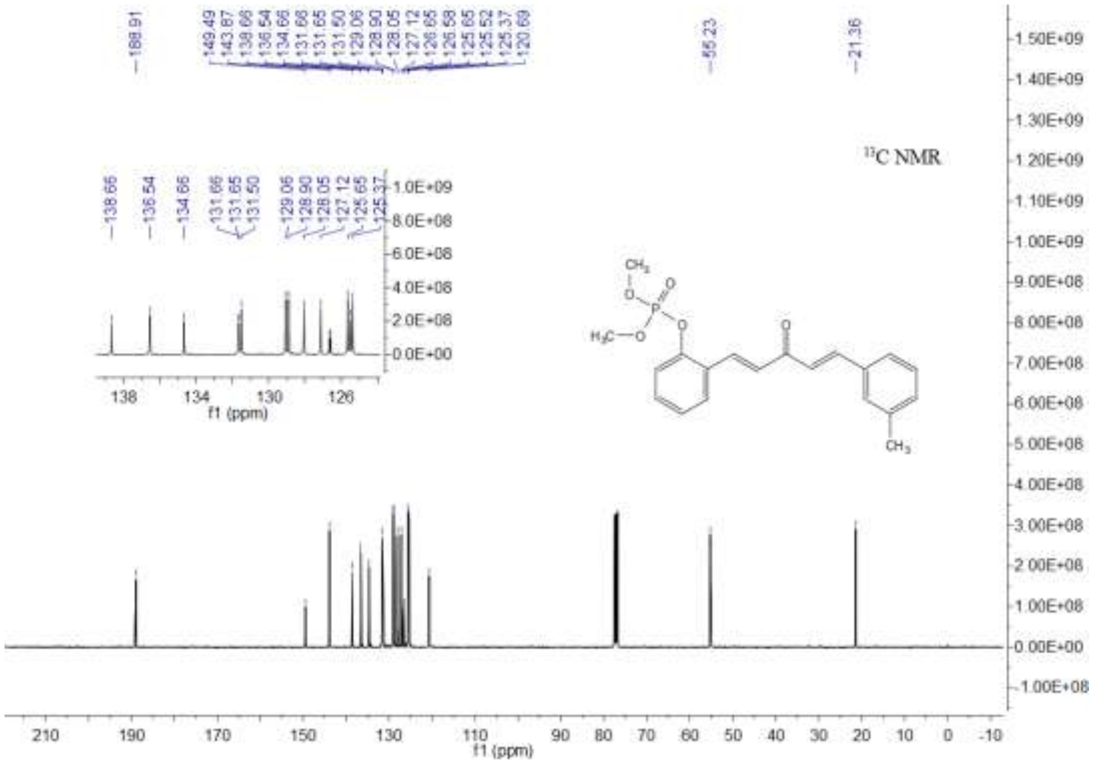

^{13}C NMR of compound **3r**

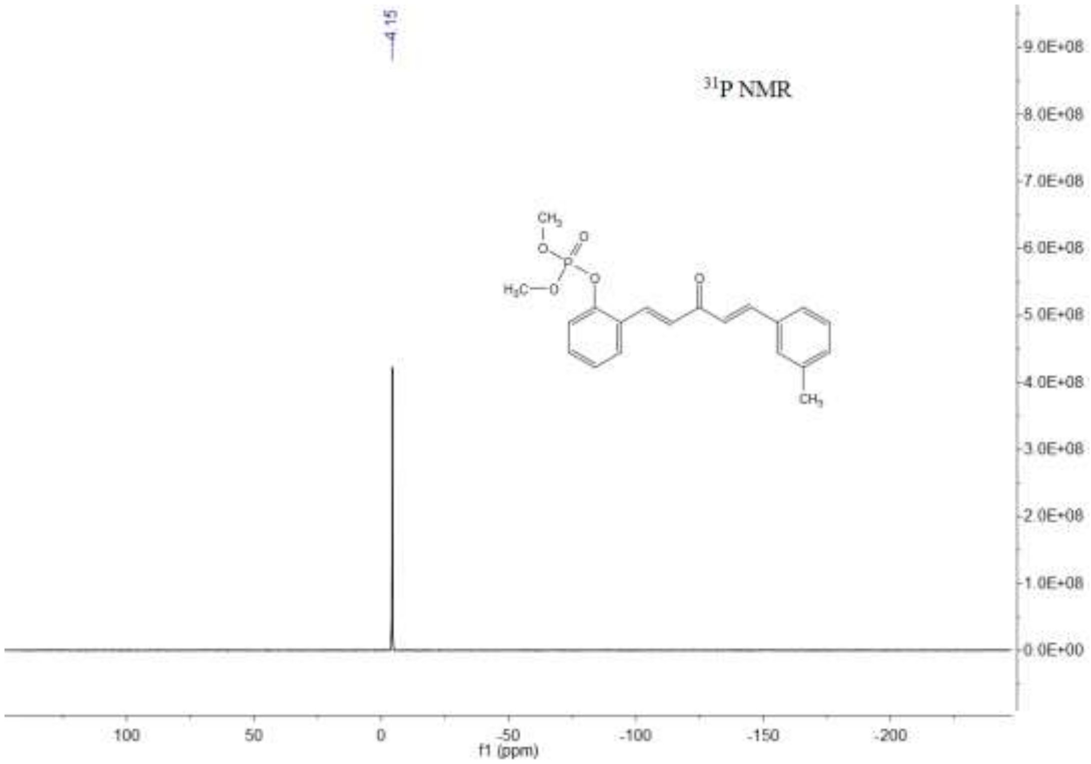

^{31}P NMR of compound **3r**

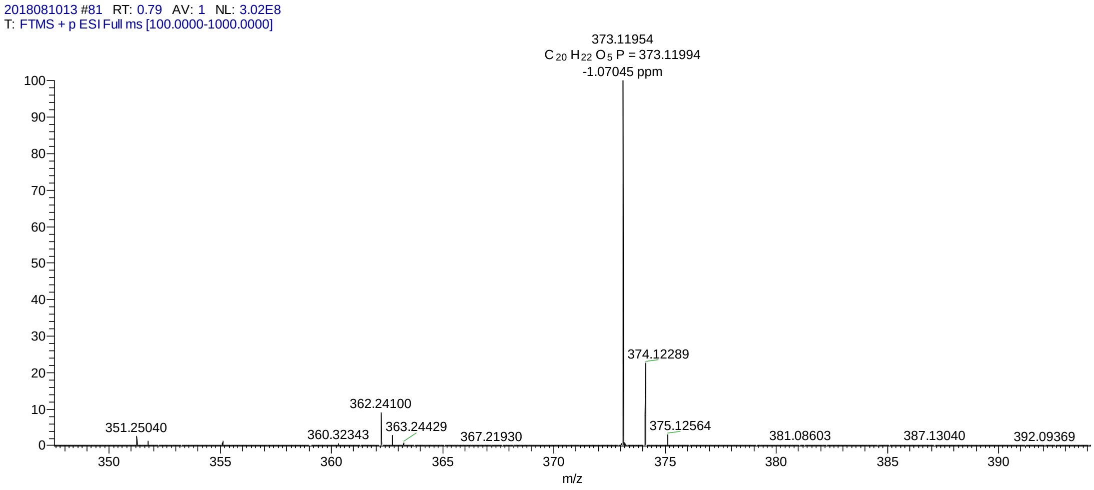

HRMS of compound **3r**

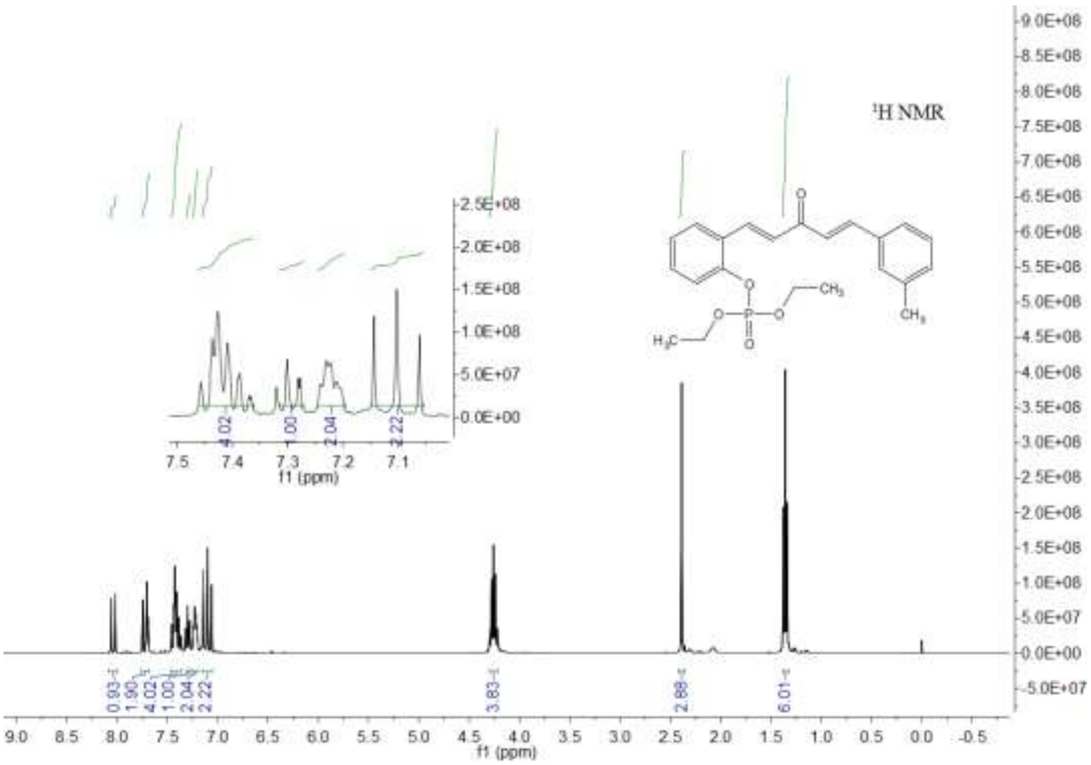

^{1}H NMR of compound **3s**

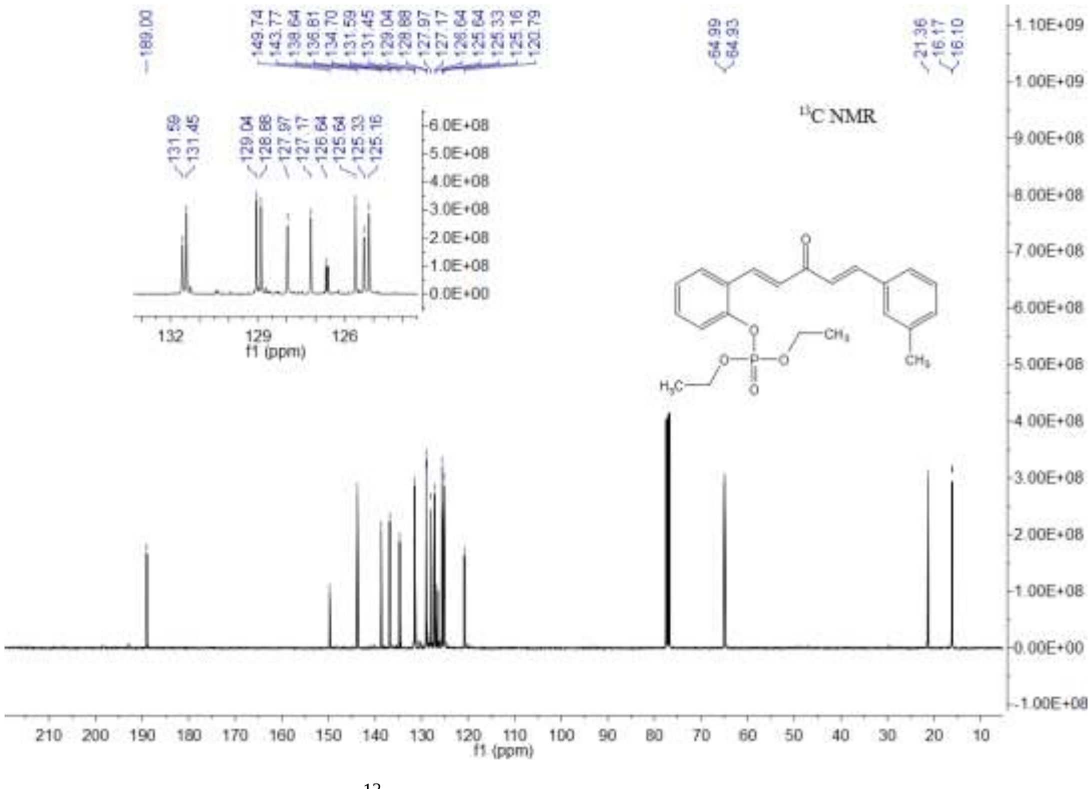

^{13}C NMR of compound **3s**

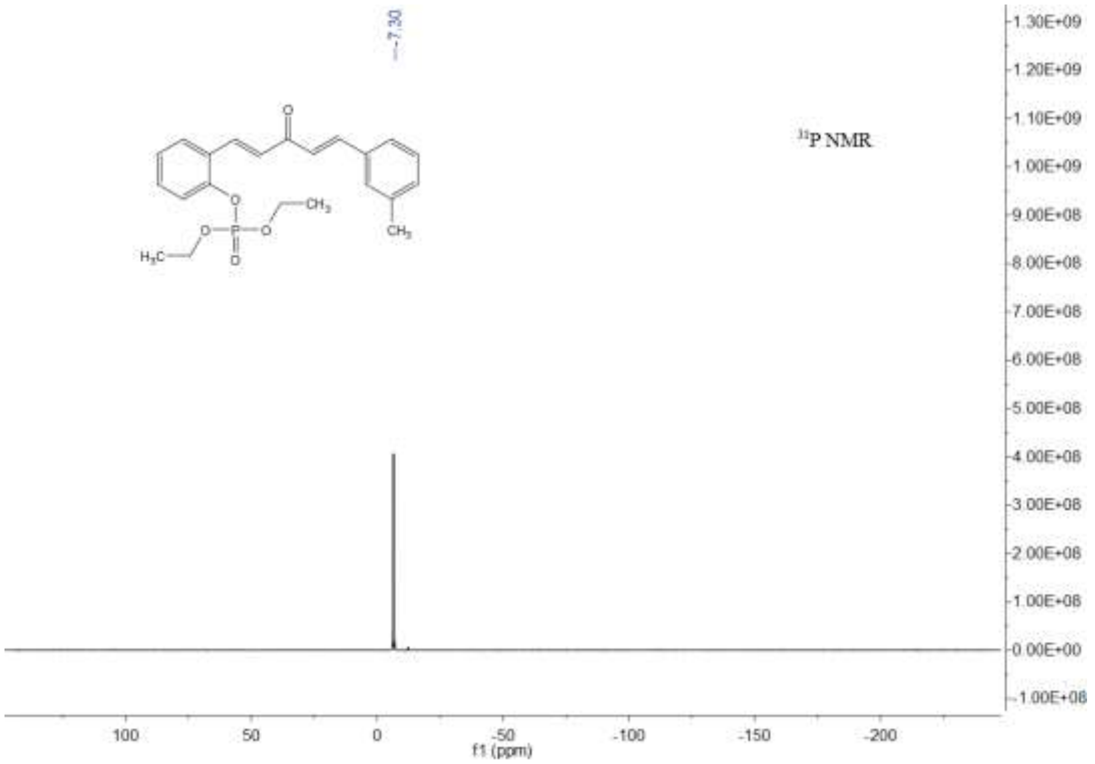

^{31}P NMR of compound **3s**

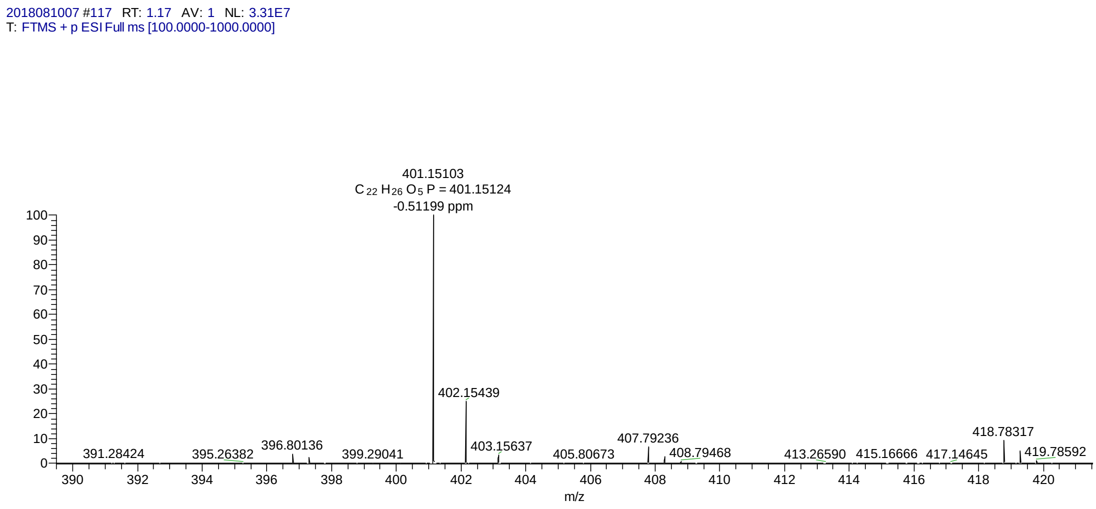

HRMS of compound **3s**
